# Changes in Data Sharing and Data Reuse Practices and Perceptions among Scientists Worldwide

**DOI:** 10.1371/journal.pone.0134826

**Published:** 2015-08-26

**Authors:** Carol Tenopir, Elizabeth D. Dalton, Suzie Allard, Mike Frame, Ivanka Pjesivac, Ben Birch, Danielle Pollock, Kristina Dorsett

**Affiliations:** 1 School of Information Sciences, University of Tennessee, Knoxville, Tennessee, United States of America; 2 Center for Information & Communication Studies, University of Tennessee, Knoxville, Tennessee, United States of America; 3 School of Information Sciences, University of Tennessee, Knoxville, Tennessee, United States of America; 4 US Geological Survey, Oak Ridge, Tennessee, United States of America; 5 Grady College of Journalism and Mass Communication, University of Georgia, Athens, Georgia, United States of America; 6 School of Information Sciences, University of Tennessee, Knoxville, Tennessee, United States of America; 7 School of Information Sciences, University of Tennessee, Knoxville, Tennessee, United States of America; 8 School of Information Sciences, University of Tennessee, Knoxville, Tennessee, United States of America; VU University Amsterdam, NETHERLANDS

## Abstract

The incorporation of data sharing into the research lifecycle is an important part of modern scholarly debate. In this study, the DataONE Usability and Assessment working group addresses two primary goals: To examine the current state of data sharing and reuse perceptions and practices among research scientists as they compare to the 2009/2010 baseline study, and to examine differences in practices and perceptions across age groups, geographic regions, and subject disciplines. We distributed surveys to a multinational sample of scientific researchers at two different time periods (October 2009 to July 2010 and October 2013 to March 2014) to observe current states of data sharing and to see what, if any, changes have occurred in the past 3–4 years. We also looked at differences across age, geographic, and discipline-based groups as they currently exist in the 2013/2014 survey. Results point to increased acceptance of and willingness to engage in data sharing, as well as an increase in actual data sharing behaviors. However, there is also increased perceived risk associated with data sharing, and specific barriers to data sharing persist. There are also differences across age groups, with younger respondents feeling more favorably toward data sharing and reuse, yet making less of their data available than older respondents. Geographic differences exist as well, which can in part be understood in terms of collectivist and individualist cultural differences. An examination of subject disciplines shows that the constraints and enablers of data sharing and reuse manifest differently across disciplines. Implications of these findings include the continued need to build infrastructure that promotes data sharing while recognizing the needs of different research communities. Moving into the future, organizations such as DataONE will continue to assess, monitor, educate, and provide the infrastructure necessary to support such complex grand science challenges.

## Introduction

The topic of data sharing is an important part of modern scholarly debate. The open access movement, focused on making published research articles freely available, has grown to encompass the data associated with the research [1]. Discussion topics include how to share data, who shares their data, and the benefits and pitfalls of data sharing. The prospect of widely available data for all has garnered the attention of such publications as *Nature* [2,3] and *Science* [4], in which whole feature sections have been devoted to the topic of "big data," as well as *The Economist* [5] [6] and *The Atlantic* [7]. The idea of the "fourth paradigm" of scientific research [8] posits that data-intensive, cross-domain research is the way of the future, and collaborative, distributed networks of researchers will work together to tackle scientific research problems. Data sharing is an essential component of the direction in which science is moving.

Beginning in 2009, the members of the Usability and Assessment Working Group of the Data Observation Network for Earth (DataONE) project conducted a survey of scientists’ data sharing practices and perceptions of the barriers to data sharing. Funded by the National Science Foundation, DataONE is one of the original DataNet partners working to provide sustainable science data preservation and access. DataONE, a multi-organization collaboration focusing on the preservation and curation of environmental and Earth science data, works to “ensure the preservation, access, use and reuse of multi-scale, multi-discipline, and multi-national science data via three primary cyberinfrastucture elements and a broad education and outreach program” [9]. The DataONE Usability & Assessment Working Group focuses on the research development and implementation of processes, systems, and methods to ensure DataONE products and services meet network goals, including appropriate community involvement [10]. These surveys were conducted as part of that mission.

Results from the original study, hereafter referred to as the baseline, were published in the *PLOS ONE* article, "Data Sharing by Scientists: Practices and Perceptions" [11]. The article has been widely cited in the years since, with 81 citations indexed in the Elsevier abstract and citation database Scopus as of the time of this writing, and 24,531 *PLOS* and PubMed Central page views and downloads, according to data on the *PLOS* website. The original survey pre-dated the National Science Foundation’s requirement that all proposals include a plan for data management and sharing of the products of research [12].

The current article is the result of a planned follow-up study, scheduled to take place 3–4 years after the baseline survey, to examine how these practices and perceptions have changed, or not changed, in the meantime. The follow-up survey was taken after the 2011 NSF requirements were in effect, but prior to the implementation of new government mandates surrounding data management and sharing [13]. Given the quickly changing environment of scientific research, we sought to measure and report any changes in scientists’ data sharing perceptions and practices that have occurred in the intervening years.

Broader participation in data sharing and data reuse raises important questions about what influences data sharing within scientific communities, how those outside of a specific scientific community use and make sense of shared data from another community, and how scientists feel about using data collected by others [14]. The current study addresses the questions of how these practices and perceptions are changing as the conversations surrounding the issue increase. Specifically, this study seeks to answer the following research questions:

RQ1a: How have researchers’ perceptions about data sharing and reuse changed between the 2009/2010 (baseline) study and the 2013/2014 (follow-up) study?
RQ1b: How have researchers’ data sharing and reuse behaviors changed between the 2009/2010 (baseline) study and the 2013/2014 (follow-up) study?
RQ1c: How have researchers’ satisfaction with processes within the research lifecycle changed between the 2009/2010 (baseline) study and the 2013/2014 (follow-up) study?
RQ1d: How have researchers’ perceptions of organizational support changed between the 2009/2010 (baseline) study and the 2013/2014 (follow-up) study?
RQ2a: What differences across age groups currently exist in terms of data sharing and reuse perceptions, behaviors, and perceptions of organizational support?
RQ2b: What differences across geographic region currently exist in terms of data sharing and reuse perceptions, behaviors, and perceptions of organizational support?
RQ2c: What differences across subject disciplines currently exist in terms of data sharing and reuse perceptions, behaviors, and perceptions of organizational support?

For researchers, the inclination toward data sharing is context-dependent. Variations in institutional support, the available technological infrastructure, and interactions with other researchers are all factors that affect researchers’ desire and ability to make their data available to others [15,11]. In order to normalize the sharing and reuse of digital data, the research community must first understand the benefits of—and barriers to—data sharing for individual scientists. From there, the scientific community can derive a model of data-sharing that is based on the drivers and barriers perceived by stakeholders, and effectively put practices into place that will encourage beneficial data sharing and reuse [16].

### Data Sharing and Withholding

"Data sharing" occurs when scientists intentionally make their own data available to other people for their use in research or other related scientific endeavors. Scientists share data by including their datasets with published articles, posting data on institutional or personal websites, depositing datasets into repositories, or sending data in response to personal requests from fellow researchers [17]. The largest discrepancy in current practices of data sharing is between what people believe should be done with data and what is actually being done [18]. Despite an overall belief that scientific data should be available for use beyond their original purpose [14], scientists are often protective of their data and may not readily engage in sharing practices [19,11]. Although there is some evidence that attitudes toward data access predict increased willingness to share data [20], the current examination of both perceptions and practices regarding data sharing is warranted.

### Individual Factors

Scientists do not seem to mind sharing data that are directly requested by a peer, as this exchange provides the original researcher assurance that they will receive credit for their work and that their data will not be misused [21]. Yet even with these assurances, such direct requests for data are rare [17].

In terms of more widespread data sharing, researchers may hesitate to make data available due to possible scrutiny that could arise from mistakes that others find in their data [22]. In addition, scientists may not feel equipped to navigate required data management systems, and may be unfamiliar with the appropriate metadata standards to ensure that their data is easily found and utilized [23,24]. In fact, the 2009/2010 baseline study showed that over half of respondents used no metadata at all to archive their data [11]. A lack of established, shared standards for descriptive metadata and for data formatting can be a particular concern for researchers doing interdisciplinary science or working with large, complex datasets [16,21,24,25]. The variety of available research data repositories, which differ not only in content but also in attributes, and the lack of integration between them may likewise present a challenge to researchers both in terms of data discovery and the effort required to share their own data [26,27]. These technological challenges may prevent scientists from sharing their data; however, a larger issue may simply be that scientists are unaware of the value their data may have for other researchers [23] and see no need to make their data public.

In spite of the barriers mentioned above, there are many advantages for individual scientists to share data. For researchers who are looking to improve their professional stature, data sharing offers opportunities to be listed as a co-author, be cited by more prominent members of their field, and receive formal acknowledgement [15]. When individuals store their data in repositories, their work gains the potential to be shared across regional, national, and international boarders. Research, therefore, can move forward to benefit the global scientific community [23,24]. The establishment of cooperative data sharing consortia and the use of citable data publication formats may also serve to incentivize data sharing for individual scientists [22,28].

### Institutional and Policy Factors

In the past, few disciplines had funding or publication requirements to share the data on which their research is built [17]. This is changing as a number of recent policies and recommendations related to data sharing call for open access to data resulting from publicly funded research. These include those of the National Science Foundation [12], the U.S. Office of Science and Technology Policy [13], the Research Councils U.K. [29], the Australian Research Council [30], and the European Commission [31].

Open access to data has been promoted as a public good, a cost-saving measure, and a way to increase transparency and enable verification of prior research results [1,32,33]. However, past research has found that despite widespread support for data preservation, sharing, and reuse in principle, in practice data sharing policies have had little impact [1]. Policies for data management and sharing have tended to vary by institution and by research sponsor, and compliance may be inconsistently enforced [1,11]. This may be changing, however, as conducting federally-funded versus corporate, private, or institutionally funded research does predict increased willingness to share data [20].

Further, policy-level requirements for openly available data raise a number of legitimate concerns. Depending on the nature of the data, issues of security, intellectual property, confidentiality, potential data misuse or misinterpretation, informed consent when future uses of the data are unknown, and inability to ensure human subject privacy via anonymization may come into play [1,11,21,33,34,35]. The possible limited value of raw data outside of its original context [1] and the effort required to make data intelligible and usable for an audience including non-specialists [33] are also potential barriers. Given the factors that are shaping the landscape of data sharing, it is critical to understand the perceptions and behaviors of the researchers who collect, analyze, and curate the data.

## Methods

### Participants and Sampling

Both the baseline and follow-up studies relied on snowball and volunteer sampling to recruit participants. To recruit for both the baseline and the follow-up studies, an email was distributed by DataONE team members to contacts including deans, department chairpersons, and program managers at various federal science agencies, major universities, and research institutions around the world. The email to the agency contacts contained a link to the survey, which they were asked to forward and distribute to faculty, lecturers, post- doctoral research associates, graduate students, undergraduate students, or researchers within the research or academic institution. In addition, the survey was distributed via a variety of environmental science listservs and blogs.

Demographic and work-related descriptions of the participants from both the baseline and the follow-up surveys are contained in S1 Appendix, Table A (Age group), Table B (Continent where employed), Table C (Primary Subject Discipline). Participants were primarily from the United States (baseline: *n* = 899, 68.9%; follow-up: *n* = 482, 49.6%; total: *n* = 1381, 60.7%), were employed in the academic work sector (baseline: *n* = 1058, 80.5%; follow-up: *n* = 746, 74.3%; total: *n* = 1804, 77.8%) as Assistant, Associate, or full Professors (baseline: *n* = 615, 46.9%; follow-up: *n* = 320, 32.7%; total: *n* = 935, 40.9%), and conducted research that was primarily funded through the federal/national government (baseline: *n* = 870, 67.0%; follow-up: *n* = 568, 62.5%; total: *n* = 1438, 65.1%).

### Procedure

The baseline survey [10] was open from October 27, 2009 to July 31, 2010, and the follow-up survey was open for responses from October 17, 2013 to March 19, 2014. Distribution of the 2009/2010 baseline survey was staggered, and the link was sent out at different times to different institutions and distribution lists. Because the follow-up survey link was sent to all institutions and distribution lists at once, it was open for a shorter duration of time than the baseline survey. Both were administered to multinational samples of scientific researchers. After cleaning, which involved eliminating respondents who didn’t answer any question or answered only one question, we had a total of 1,329 responses for the baseline and 1,015 responses for the follow-up. Both studies were approved as anonymous online surveys by the University of Tennessee Human Subjects Institutional Review Board. Findings are reported aggregately, and no identifying information was collected. Respondents were free to withdraw from the surveys at any time, and were not forced to answer any questions in order to progress through the surveys. An informed consent statement preceded the survey (S2 Appendix), which concluded with the statement: “By proceeding to the survey I acknowledge that I have read the above statements, I am 18 years old or older, and I agree to participate.”

### Survey Instrument

The questions in the follow-up survey assessment are largely the same as in the original baseline survey [10]; however, a few questions and answer options were changed, omitted, or added to the current survey to refine and expand the overall instrument. These are pointed out in the results section as they a reported. The questionnaire (S2 Appendix) was designed to capture scientists’ perceptions about data sharing, the data sharing practices in which they engage, satisfaction with different stages of the research lifecycle, and perceptions of organizational support for research processes. Demographic and background questions that are analyzed in this paper include year of birth, work sector (e.g., government, nonprofit, academic, etc.), primary subject discipline (e.g., environmental science, medicine, social science, etc.), professional position, primary funding agency, and country of employment. Year of birth was collapsed into a new variable of three age categories based on the categories used in the baseline study. Country of employment was also collapsed into a new continents variable to enable more accurately operationalize geographic region as a variable.

The first half of the results (RQ1a, 1b, 1c, and 1d) reports changes from the baseline to the follow-up. Direct comparisons were made where questions were the same from baseline to follow-up, and descriptions will be provided for those questions that differed from the baseline or were added to the current survey. RQ1a asks how researchers’ perceptions about data sharing and reuse have changed over the last three years. Questions about perceptions of data sharing capture opinions about the value of shared data, willingness to share and reuse others’ data, and the perceived risks associated with reusing others’ datasets. Examples of Likert-type scale items include *Lack of access to data generated by other researchers or institutions is a major impediment to progress in science; I would be willing to place at least some of my data into a central data repository with no restrictions;* and *Data may be used in other ways than intended*.

RQ1b examines how researchers’ data sharing and reuse behaviors have changed since the baseline study. Questions about data sharing practices capture scientists’ self-reported behaviors, including how accessible their data is (Likert-type scale including *Others can access my data easily*), how much data they store in various locations (None, Some, Most, All options for locations such as *On the PI’s server*, *On paper in my office*, and *In an institution-based repository*), and what metadata standards they use (“Check all that apply,” including *Dublin Core*, *metadata standardized within my lab*, and *none*). RQ1c examines how scientists’ satisfaction with different stages of research has changed since the baseline study. These items measured satisfaction with individual processes within the research and data lifecycle. Examples of Likert-type scale items include *I am satisfied with the processes for collecting my data* and *I am satisfied with the tools for preparing metadata*. RQ1d examines how perceptions of organizational involvement with data management support and policies have changed since the baseline study. Likert-type scale items include *My organization has a formal process for storing data beyond the life of the project (long-term)* and *My organization or project provides training or assistance on best practices for data management*.

When examining changes from the 2009/2010 baseline study to the current follow-up study, it is important to note that the follow-up study contained data from a significantly higher proportion of international respondents than the baseline. A Chi-square test for independence comparing the proportion of North American vs. non-North American respondents from both surveys was statistically significant [*X* ^*2*^(1, N = 2277) = 39.26, *p* < .001]. Therefore, in further analysis that included continuous variables, we controlled for this effect to avoid type I error in our conclusions about baseline to follow-up differences.

In the second half of the results, we examine how different demographic factors, including age, geographic region, and subject discipline impact data sharing perceptions and behaviors, as well as perceptions of organizational involvement in the current study. RQ2a examines differences across age groups in terms of data sharing and reuse perceptions and behaviors, as well as differences in perceptions of organizational support. RQ2b examines these same differences across geographic regions (measured by continent), and RQ2c examines these differences across subject disciplines.

For Likert-type scale questions measuring agreement (agree strongly, agree somewhat, neither agree nor disagree, disagree somewhat, and disagree strongly) with specific items, the original questionnaire also provided a sixth option of “not sure” or “don’t know.” This was intended to provide respondents with an opportunity to skip the question if they either did not know how they felt about the item or did not understand the question. Because the “don’t know/not sure” option does not provide any information about level of agreement, these answers were treated as missing data in the analysis (except where frequencies are reported). For questions that were either missing from or asked differently in the baseline study, only descriptive analysis of the current study is included as comparisons cannot be made.

Results were analyzed using the SPSS 22 statistical software package. To compare the baseline to follow-up survey results, data from both surveys were merged into a new SPSS file with a separate variable created to indicate either baseline or follow-up responses for each question that was examined.

## Results

### Changes in Data-Related Perceptions and Practices, 2009/2010 to 2013/2014

#### Data sharing and reuse: Perceptions

Respondents answered a series of questions designed to capture their perceptions about data sharing and reuse. These responses suggest that researchers have more positive perceptions about the value of shared data and willingness to share and reuse others’ data. There is also increased concern with the risks associated with reusing others’ datasets.

RQ1a: How have researchers’ perceptions about data sharing and reuse changed between the 2009/2010 (baseline) study and the 2013/2014 (follow-up) study? Since the baseline, researchers are more likely to perceive that the lack of access to others’ data is a major impediment to scientific progress. However, researcher perception about how the lack of access to data affects their own work is unchanged. When it comes to the value of shared data (questions 20.1 and 20.2), a MANOVA indicates that there has been a significant change in perceptions since the baseline study. Specifically, univariate ANOVAs show that agreement with the statement that *lack of access to data generated by other researchers or institutions is a major impediment to progress in science* (question 20.1) has increased from the baseline to the follow-up. There has been no significant change in agreement with the statement that *lack of access to data generated by other researchers or institutions has restricted my ability to answer scientific questions* (question 20.2; see Table D in S1 Appendix).

Researchers indicate significantly more willingness to share and reuse data than in the baseline survey. Respondents were asked about willingness to engage in scientific data sharing and reuse (questions 21.1–21.8). A MANOVA reveals that there has been a significant shift in agreement about these topics since the baseline study (see Table E in S1 Appendix).). Univariate ANOVAs within the omnibus MANOVA show that there has been an increase in agreement since the baseline study with the statements *I would use other researchers’ datasets if their datasets were easily accessible* (question 21.1), *I would be willing to place at least some of my data into a central repository with no restrictions* (question 21.2), *I would be willing to place all of my data into a central repository with no restrictions* (question 21.3), *I would be willing to share data across a broad group of researchers* (question 21.6), and *it is appropriate to create new datasets from shared data* (question 21.8). For a few statements that addressed restrictions or conditions on sharing, respondents indicated significantly less agreement than the baseline, including *I would be more likely to make my data available if I could place restrictions on access* (question 21.4) and *it is important that my data are cited when used by other researchers* (question 21.7).

Researchers are more concerned about the possible risks associated with sharing data, and the potential for misuse and misinterpretation (questions 20.3–20.5). A MANOVA was run to examine differences in these items from the baseline to the follow-up study, with the omnibus test showing a significant difference (see Table F in S1 Appendix).). When reusing data, univariate ANOVAs show that respondents’ agreement with the idea that *data may be misinterpreted due to complexity of the data* (question 20.3) has seen a significant increase over time. Respondents’ perceptions that data may also be *misinterpreted due to poor quality of the data* (question 20.4), have also increased from the baseline to the current study. Finally, respondents expressed significantly higher agreement in the current study that *data may be used in ways other than intended* (question 20.5) than in the baseline.

Reflecting the increased focus on data in the scientific community, this study explored the barriers to data sharing in more depth than the baseline (question 13b). Respondents who do not make all of their data available (question 13) were asked, “If all or part of your data are not available to others, why or why not?” and then given a series of possible reasons. For these items, chi-square tests for independence were run to assess changes in the prevalence of each barrier from the baseline to the follow-up surveys. Where comparisons can be made, it is clear that perceptions of barriers have changed. There is less concern about lack of funding (question 13b.1) and insufficient time (13b.4). The concern about the need to publish before making the data available is now the top-ranked barrier. There is also greater concern about the barriers related to people not needing the data and to researchers not having the rights to make data public. Barriers to data sharing that have significantly increased since the baseline include the idea that people don’t need them (question 13b.3) and that respondents don’t have the rights to make data public (question 13b.8) (see Table G in S1 Appendix).

There are some conditions that scientists believe to be important in their decision to share data. Receiving acknowledgement or a formal citation for the data is essential to researchers who share data. In order to understand more about these conditions, participants were asked to respond to a series of statements indicating whether certain stipulations are fair conditions for others to use their data (question 16). In the follow-up survey, respondents were given the option of “not sure,” which was not included in the baseline study, in addition to “yes” and “no.” This allowed us to gain perspective on any ambivalence respondents may have toward fair use of their data. In addition, the follow-up study does not ask about conditions for respondents to use *others’* data, which was asked in the baseline. Respondents in the baseline study, therefore, may have been primed to think differently about fair use of their data, so comparisons to the baseline were not made. Results for these items will only be reported as they exist in the current study (see Table H in S1 Appendix).

The items described different conditions related to receiving credit, control, information, and funding. In the follow-up study, it appears that there is little controversy over whether a data provider or funding agency should receive acknowledgement (question 16.2; 87.7% yes) or citation (question 16.3; 85.1% yes) in published work that uses shared data, but perceptions about extending co-authorship credit on publications (question 16.1) were mixed (see Table H in S1 Appendix). On the other hand, contributing to the cost of data acquisition, cost, or retrieval (question 16.6) was only thought to be a fair condition for use of data by 14.2% of respondents. Other items are reported in table 1.6.

#### Data sharing and reuse: Practices

RQ1b: How have researchers’ data sharing and reuse behaviors changed between the 2009/2010 (baseline) study and the 2013/2014 (follow-up) study? Nearly three-quarters of the respondents in the follow-up study indicate that they make at least some of their data available to others (question 13; see Table I in S1 Appendix). This is reflected in the growth of sharing and access patterns since the baseline survey. But it is also of fundamental importance that we understand how data sharing behaviors have changed since the original baseline study. In both the baseline and follow-up surveys, participants were asked to indicate their level of agreement with the statements *I share my data with others* and *others can access my data easily* (questions 15.1 and 15.3) in order to understand how accessible participants perceive their data to be (see Table J in S1 Appendix) (O*thers need permission to access my data* [question 15.2] was not presented in the baseline). A MANOVA was run to assess the baseline to follow-up differences for *I share my data with others* and *others can access my data easily*, the results of which indicate significant increases in data sharing practices since the baseline. Specifically, univariate ANOVAs show that response to *I share my data with others* has increased in agreement from the baseline to the current survey, as has *others can access my data*.

Researchers still depend primarily on personal storage options although cloud storage options such as Dropbox have emerged (question 12). For each possible given location, respondents were given the options of “none,” “some,” “most,” or “all,” and we were able to derive a mean amount of data stored in each location; however, due to a different set of location options being provided in the baseline questionnaire, the results only describe the follow-up results. It appears that respondents store the highest amount of data *on my personal computer*, *on my institution’s server*, *on the principal investigator’s server*, and *on paper in my office* (see Table K in S1 Appendix).

Researchers can facilitate accessibility to their datasets by adding descriptive metadata (question 8), which can be used to help others locate and utilize the data. In the baseline study, when respondents were asked which metadata standards they use to describe their data, over half of the respondents (50.9%) chose “No metadata standard” [11]. The percentage of respondents who indicated that they do not use metadata has fallen slightly (47.9%). While the change is not statistically significant, continuing to monitor these changes will be useful to determine whether this is an early indicator of changes in practice. It is particularly interesting given that chi-square tests for independence show that there have been significant increases in the proportion of participants using Dublin Core, as well as in those who indicate using International Standards Organization and “other” metadata standards (see Table L in S1 Appendix).

#### Satisfaction with data practices

RQ1c: How have researchers’ satisfaction with processes within the research lifecycle changed between the 2009/2010 (baseline) study and the 2013/2014 (follow-up) study? Comparing results from the two studies demonstrates that there are some significant changes in the level of satisfaction with different processes within the research and data lifecycle. There is less satisfaction with long-term data storage processes and tools for preparing documentation, and processes for searching for respondents’ own data; however, respondents continue to be the least satisfied with *processes for storing data beyond the life of the project (long-term)* (question 14.4) and *tools for preparing metadata* (14.7). A MANOVA was run (questions 14.1–14.11) to test changes in satisfaction from baseline to follow-up for each given aspect of research and data practices, and the omnibus test yielded significant results. Individual univariate ANOVAs showed that satisfaction with *long-term* data storage processes (question 14.7) has decreased significantly, as well as satisfaction with *tools for preparing documentation* (question 14.8). There has also been a decrease in satisfaction with *the processes for searching for my own data* (question 14.5) (see Table M in S1 Appendix).

#### Perceptions of organizational support

RQ1d: How have researchers’ perceptions of organizational support changed between the 2009/2010 (baseline) study and the 2013/2014 (follow-up) study? There is greater agreement about the need for training on data management best practices, and the need for research organizations to provide the funding for data management. There were several questions intended to explore scientists’ perceptions of institutional support and policies surrounding data management (questions 17, 18, 19–1, and 19–2). A MANOVA was run to test changes from the baseline to the follow-up survey in mean agreement with each statement pertaining to data management support and policies. There was a statistically significant difference in agreement about level of organizational involvement from the baseline to the current survey, (see Table N in S1 Appendix). Univariate ANOVAs reveal that specifically, when it comes to providing *training on best practices for data management* (question 18.1), respondents expressed higher agreement in the follow-up study than in the baseline that this training is provided by their organization. In addition, there has been a significant change in agreement that organizations *provide the necessary funds to support data management during the life of the research project* (question 19–1.1), with an increase in mean agreement from the baseline to the follow-up study.

### Demographic Groups in Relation to Data Reuse and Sharing: Follow-Up Survey

Scientists’ age, geographic region, and subject discipline may play a role in determining perceptions and practices toward data sharing and reuse. In the second half of the results, we examine differences in data sharing practices and perceptions, as well as perceptions of organizational support, across these different groups within the follow-up study.

#### Age

RQ2a asks: What differences across age groups currently exist in terms of data sharing and reuse perceptions, behaviors, and perceptions of organizational support? Using three age categories (22–39; 40–49; 50 and older), we examined the possible impact of age on data sharing behaviors, perceptions about sharing and reusing data, satisfaction with data lifecycle processes, and perceptions of data management support and policies at the organizational level.

Perceptions about data sharing and reuse do vary between younger and older respondents. Younger researchers are more concerned than older researchers are about the lack of access to data. Younger researchers also express more interest in using the datasets of others if access were easy and in sharing their own data if they could place restrictions. These conditions include gaining co-authorship, having the opportunity to collaborate, having the opportunity to review the other researcher’s results and make suggestions and to grant legal permission. It is notable that data-sharing behavior also varies across age groups, but it seems to be inverse to the perceptions. While younger researchers are more concerned about lack of access, they are the least involved in data sharing. In fact, data-sharing behavior actually increases significantly with each older age group. So while younger researchers express a higher interest in sharing their own data, they are also more interested in requiring others to get permission to access their data. While the older researchers are sharing their data, they are far less more likely to provide no metadata for their data. Perceptions of barriers to data sharing also vary by age group. Younger researchers are more concerned about being able to publish their own results first, while older researchers are more concerned about lack of funding.

Respondents were asked about the value of data sharing and reuse in terms of the impact of a lack of access to others’ data (questions 20.1–20.2). With age category as the independent variable, a MANOVA indicated a significant difference across the three age groups (see Table O in S1 Appendix). Specifically, univariate ANOVAs with Tukey’s post hoc analyses indicated that for *lack of access to data generated by other researchers or institutions is a major impediment to progress in science* (question 20.1) those ages 22–39 expressed significantly higher agreement than those who were 50 or older (*p* = .007). In addition, the same tests showed that those ages 22–39 expressed significantly higher agreement than those 50 or older (*p* = .001) with the statement *lack of access to data generated by other researchers or institutions has restricted my ability to answer scientific questions* (question 20.2).

Willingness to engage in scientific data sharing and reuse (question 21) also varied significantly across the three age groups (see Table P in S1 Appendix). Among the eight items grouped within the omnibus MANOVA, univariate ANOVAs with Tukey’s post hoc analyses indicated significant pairwise differences for two of the items. For the statement *I would use other researchers’ datasets if those datasets were easily accessible* (question 21.1), those 22–39 agreed more strongly than those 50 and older (*p* = .010). Also, for *I would be more likely to make my data available if I could place restrictions on access* (question 21.4), those 22–39 agreed more strongly than those 50 and older (*p* = .004). Respondents also were asked about perceived risks of data reuse (questions 20.3, 20.4, and 20.5), for which a MANOVA indicated no significant difference across age groups (see table Table Q in S1 Appendix).

In terms of barriers to data sharing (question 13b), chi-square tests for independence were run to examine age group as a predictor of each given possible barrier. For both *lack of funding* (question 13.1) and *I need to publish first* (question 13.10), there were statistically significant differences across the three age groups in those who selected these as barriers. Adjusted standardized residuals showed that a significantly smaller proportion of the youngest group (ages 22–39) thought *lack of funding* was a barrier, while a significantly higher proportion of the oldest group (ages 50 and older) saw this as a barrier. A significantly higher proportion of the youngest group saw the *need to publish first* as a barrier, while this was significantly less of a barrier for those 50 and older (see Table R in S1 Appendix for percentages and adjusted standardized residuals).

Respondents were asked about the specific conditions under which they would make their data available to others (question 16; see Table S in S1 Appendix). Chi-square tests for independence were used, along with adjusted standardized residuals, to examine differences in response across the three age groups. All percentages of “yes” and corresponding adjusted standardized residuals are reported in table 2.5. The percentage of those who selected “yes,” “no,” or “not sure” for four of the 12 conditions differed significantly by age group. Requiring *co-authorship on publications resulting from the data* (question 16.1) was selected by significantly more of those ages 22–39, and by a significantly smaller proportion of those age 50 and older. Having the *opportunity to collaborate on a project using the data* (question 16.4) also differed significantly by age group, with a significantly higher proportion of those ages 22–39 selecting “yes” for this condition, and a lower than expected proportion of those ages 50 and older selecting “yes.” The condition that *results based (at least in part) on the data could not be disseminated without the data provider having the opportunity to review the results and make suggestions or comments*, *but approval not required* (question 16.5) was again selected (“yes”) by a higher than expected proportion of those in the youngest age group (22–39), and by a lower than expected proportion of those 50 and older. Finally, the condition that *legal permission for use of data is obtained* (question 16.10) was selected by a significantly higher proportion of those ages 22–39, and a significantly lower proportion of those ages 50 and older.

Age was also examined in terms of data sharing practices. When asked how much of their data they make available to others (question 13; 1 = “none,” 2 = “some,” 3 = “most,” 4 = “all”), a one-way analysis of variance (ANOVA) with age category as the independent variable revealed a significant difference across age groups (see Table T in S1 Appendix). Tukey’s post-hoc analyses showed significant differences between each pairing of the three age groups, with those 50 and over sharing the most data, and those 22–39 sharing the least amount.

In addition, the level of agreement expressed in response to questions about data sharing behaviors (question 15) and access varied significantly according to age (see Table U in S1 Appendix). In response to *I share my data with others* (question 15.1), Tukey’s post-hoc analyses showed that those 50 and over expressed significantly higher agreement than those ages 22–39 (*p* = .001). For *others need permission to access my data* (question 15.2), a Tukey’s post hoc test showed that agreement was significantly higher among those age 22–39 than those ages 50 and older (*p* = .002). Finally, for *others can access my data easily* (question 15.3), Tukey’s post hoc analyses showed significantly higher agreement among those 50 and older than those ages 22–39 (*p* = .028; see Table U in S1 Appendix).

The amount of data that scientists store in different locations (question 12) is also indicative of how much of their data they share. For instance, storing data on servers or in data repositories may allow other researchers to access and utilize their data. From the “none,” “some,” “most,” and “all” options, we also created a new variable by dichotomizing answers into “yes” or “no” to determine whether these storage locations were used at all. Chi-square tests for independence were run to test the differences in age groups in terms of whether or not they store data in each given location; however, none of the tests was significant (see Table V in S1 Appendix).

For the most part, the use (or lack) of metadata standards (question 8) did not vary greatly by age. Of interest within this study is whether scientists are using lab-specific metadata standards, institution-specific metadata standards, or no metadata at all, as these options may preclude accessibility to their data by others. Chi-square tests for independence were run for each of these options to compare responses across the three age groups. There was significant variation across age groups for “none.” Adjusted standardized residuals revealed that a significantly lower proportion of those ages 22–39 selected “none” than expected, compared to a significantly higher proportion of those 50 and older (see Table W in S1 Appendix for percentages and adjusted standardized residuals).

Finally, age was examined in terms of perceived organizational support. The effect of age on perceptions of organizational support for managing and storing data, data management assistance and training, data management funding, and software tools and technical support is negligible (questions 17, 18, 19–1, and 19–2; see table 2.10). A MANOVA indicated no significant difference across age groups (see Table X in S1 Appendix).

#### Geographic Location

RQ2b asks: What differences across geographic region currently exist in terms of data sharing and reuse perceptions, behaviors, and perceptions of organizational support? Geographic location was measured by the continent on which the respondents resided and worked. Because respondents were asked only for their country, the country variable was recoded into a separate “continent” variable, revealing some level of participation from all continents except for Antarctica (Note: Russian respondents were categorized as European). Geographic location, measured according to continents, was examined as a possible predictor of data sharing and reuse perceptions, data sharing and reuse practices, satisfaction with data practices, and perceptions of organizational support for data management.

Results show that researchers in different regions have different perceptions about data and different data behaviors. Researchers in Asia are more concerned than those in North America and Europe about lack of access to data restricting their ability to answer scientific questions. However Asian researchers also feel satisfied with their ability to integrate data from disparate sources to address research questions. North American researchers were more concerned about the conditions for data use; however, Asian and African researchers were more interested than North American researchers in requiring permission for others to access their data. Data storage behaviors also differ. African researchers are more likely to store data in paper formats and North American researchers are less likely to do this. Storing data in institutional repositories is more common among Asian researchers and least common among North American researchers. Barriers to data sharing also differ by region. Researchers in Africa and South America are more concerned about lacking the skills to make their data available. Finally, Asian participants perceive more organizational support than Europeans do for creating data management plans and assigning in creating metadata.

In terms of perceptions about data sharing and reuse, when asked about the impact of a lack of access to others’ data (questions 20.1–20.2), a MANOVA indicated a significant difference across geographic regions. Specifically, univariate ANOVAs within the omnibus MANOVA show that participants from different continents expressed different levels of agreement with *Lack of access to data generated by other researchers or institutions is a major impediment to progress in science* (question 20.1) and *Lack of access to data generated by other researchers or institutions has restricted my ability to answer scientific questions* (question 20.2; see Table Y in S1 Appendix). Tukey’s post hoc analyses indicate that for the latter item, those from Asia express significantly higher agreement with this statement than do respondents from both North America (*p* = .002) and Europe (*p* = .009).

Willingness to engage in scientific data sharing and reuse (question 21) also varied significantly across the different geographic region (see Table Z in S1 Appendix), as indicated by a MANOVA. Univariate ANOVAs within the omnibus MANOVA show significant differences in agreement with the statements *I would be more likely to make my data available if I could place conditions on access* (question 21.4), and *I am satisfied with my ability to integrate data from disparate sources to address research questions* (question 21.5). Tukey’s post-hoc analyses show no significant pairwise differences for the statement *I would be more likely to make my data available if I could place conditions on access;* however, for *I am satisfied with my ability to integrate data from disparate sources to address research questions*, Asian respondents indicate higher levels of agreement than those from North America (*p* = .022; see table 3.2).

Agreement about the perceived risks associated with data sharing (questions 20.3, 20.4, and 20.5) also varied significantly by geographic region. These differences were tested with a MANOVA, and univariate ANOVAs showed that only the statement *data may be used in ways other than intended* (question 20.5) differed significantly across geographic regions. Tukey’s post hoc analyses showed that North Americans agreed significantly more strongly with this statement than European respondents (*p* = .018; see Table AA in S1 Appendix).

Chi-square tests using the Monte Carlo test of significance were run to examine differences in barriers to data sharing (question 13b) across the different geographic regions (see Table BB in S1 Appendix). Only one barrier showed significant differences across geographic regions, *I have insufficient skills to make my data available* (question 13b.11). In particular, African (23.1%) and South American respondents (23.8%) selected this barrier at a significantly higher rate than the total (13.3%).

Respondents were also asked about the specific conditions under which they would make their data available to others, and were given the answer options of “yes,” “no,” or “not sure” to indicate the fairness of each condition (question 16). Geographic location had a significant impact on which conditions were thought to be fair exchanges for use of participants’ data. Utilizing chi-square tests for independence with Monte Carlo test of significance, most of the conditions varied significantly according to continent. Requiring *co-authorship* (question 16.1), *the opportunity to collaborate* (question 16.4), *results could not be disseminated without the data provider’s approval* (question 16.5), *covering part of the costs*, *providing reprints of articles* (question 16.8), *provider is given a complete list of all products that make use of the data* (question 16.9), *obtaining legal permission for use of data* (question 16.10), *mutual agreement on reciprocal sharing of data* (question 16.11), and *data provider is given and agrees to a statement of uses* (question 16.12) were all conditions that varied significantly across the different continents. Overall, North American respondents were less likely to agree that given conditions are fair, whereas Asian respondents were more likely to agree that conditions were fair. African respondents were also more likely to select “yes” for many of the proposed conditions. Exact percentages and adjusted standardized residuals for individual continents are shown in Table CC in S1 Appendix.

When it comes to actual data-related practices, differences among geographic groups in terms of sharing behaviors were negligible. When asked how much of their data they make available to others (question 13), a one-way ANOVA indicated that there was no significant difference across the different continents (see Table DD in S1 Appendix); however, the level of agreement expressed in response to questions about data sharing behaviors and access (question 15) did vary significantly by continent (see Table EE in S1 Appendix). In particular, a univariate ANOVA shows that when asked how much they agree with the statement *Others need permission to access my data* (question 15.2), Tukey’s post hoc analyses indicated some significant pairwise differences in mean agreement. Asian respondents expressed significantly higher agreement with this statement than those from North America (*p* = .000), South America (*p* = .044), and Australia/New Zealand (*p* = .014). In addition, those from North America expressed significantly lower agreement that those from Africa (*p* = .007).

The amount of data that scientists store in different locations (question 12) is also indicative of how much of their data they share. From the “none,” “some,” “most,” and “all” options, we created a new variable by dichotomizing answers into “yes” or “no” to determine whether these storage locations were used at all. Chi-square tests for independence were run to test the differences in geographic location in terms of whether or not they store data in each given location. When it comes to storing data *on paper in my office* (question 12.5), respondents from North America do this significantly less than the overall total, and respondents from Africa do this significantly more than the overall total. In addition, there was a significant difference in those who store data *on my institution’s repository* (question 12.9). Specifically, those from North America store data in this location significantly less than expected, and those from Asia store data here significantly more (see Table FF in S1 Appendix).

In terms of metadata standards (question 8), the data indicate that specific standards are more commonly used in certain geographic regions than others (see Table GG in S1 Appendix). Of interest within this study is whether scientists are using lab-specific metadata standards, institution-specific metadata standards, or no metadata at all, as these options may preclude accessibility to their data by others. Chi-square tests for independence using Monte Carlo test of significance were run for each of these options to compare responses across the three age groups, utilizing Monte Carlo’s test of significance to account for the possibility of small cell sizes. Adjusted standardized residuals were examined to identify which geographic regions differed significantly from the total (or expected) cell value. There are no significant differences across continents in terms of the proportion of researchers who use institutionally standardized metadata, lab-specific metadata standards, or no metadata at all.

Finally, in terms of perceived organizational support, A MANOVA including questions 17, 18, 19–1, and 19–2 and was run to examine the role of geographic regions in perceptions of organizational involvement and support in data management. Overall, results of the omnibus MANOVA showed that perceptions of organizational support for managing and storing data, data management assistance and training, data management funding, and software tools and technical support did vary significantly by geographic region. Specifically, univariate ANOVAs showed that for *assistance on creating data management plans* (question 18.2), there is a significant difference in agreement about the levels of support coming from respondents’ organizations. Tukey’s post hoc analyses showed a pairwise difference between European and Asian respondents, with Asian respondents expressing significantly higher agreement with this statement (*p* = .028). In addition, univariate ANOVAs show a significant difference in agreement across geographic regions with the level of organizational *assistance on creating metadata to describe my data or datasets* (question 18.3. Pairwise comparisons showed that again, Asian respondents expressed significantly higher agreement than those from Europe (*p* = .010; see Table HH in S1 Appendix).

#### Subject discipline

A final demographic variable we examined was based on the subject discipline within which respondents conduct research. RQ2c asks: What differences across subject disciplines currently exist in terms of data sharing and reuse perceptions, behaviors, and perceptions of organizational support? After cleaning and merging answers included in the open-ended “other” option, respondents fell into one of 19 different disciplines (including “other”). The most distinct pattern reveals a division between those who work with human subjects data–including Medicine/Health Science, Business, Psychology, Social Sciences, and Psychology–and those who do not. In terms of perceptions about data sharing, some human-subjects disciplines felt less strongly that lack of access to others’ data is an impediment to science. They also expressed less willingness to engage in data sharing and reuse. They were more likely to think that their data shouldn’t be made available to others, and that they don’t have the rights to make it available anyway. When it comes to data sharing behaviors, those in Psychology were significantly less likely to share their data than those in some hard sciences. Finally, those who work with human subjects data were more likely to use no metadata to describe their datasets.

Perceptions about data sharing do vary significantly across the subject disciplines within which respondents conduct research. A MANOVA was run to test these items (questions 20.1 and 20.2) with subject discipline as the independent variable, and results were significant. Individual univariate ANOVAs for each item showed that for *lack of access to data generated by other researchers or institutions is a major impediment to progress in science* (question 20.1), there were some significant pairwise differences in levels of agreement. Those in Environmental Science expressed significantly higher agreement with this statement than those in both Business (*p* = .028) and Medicine/Health Sciences (*p* = .029). There were no significant pairwise differences for *lack of access to data generated by other researchers or institutions has restricted my ability to answer scientific questions* (question 20.2), although the univariate ANOVA was significant (see Table II in S1 Appendix).

Willingness to engage in scientific data sharing and reuse was also tested using MANOVA (question 21), and varied significantly across the different subject disciplines (see Table JJ in S1 Appendix). The first item showed differences across subject disciplines with the statement that they *would use other researchers’ datasets if their datasets were easily accessible* (question 21.1), although there were no significant pairwise comparisons. For the second item, *I would be willing to place at least some of my data into a central repository with no restrictions* (question 21.2), those from Medicine/Health Sciences expressed significantly lower agreement with the statements than those in almost every other category, including Agriculture and Natural Resources (*p* = .002), Atmospheric Science (*p* < .001), Biology (*p <* .001), Computer Science (*p* < .001), Ecology (*p* < .001), Education (*p* = .047) Engineering (*p* < .001), Environmental Science (*p* < .001), Geology (*p* = .003), Hydrology (*p* < .001), and Information Science (*p* < .001).

Agreement also varied significantly across subject disciplines with the statement *I would be willing to place all of my data into a central repository with no restrictions* (question 21.3). Once again, those in Medicine/Health Sciences expressed significantly less agreement than those in Biology (*p* = .007), Computer Science (*p* = .010), Ecology (*p* = .013), Engineering (*p* = .031), Environmental Science (*p* = .004), Hydrology (*p* = .028), and Information Science (*p* = .001). For the item *I would be more likely to make my data available if I could place conditions on access* (question 21.4), there were no significant pairwise differences. For *I am satisfied with my ability to integrate data from disparate sources to address research questions* (question 21.5), overall differences were also significant, with Social Scientists being significantly less satisfied than both Atmospheric Scientists (*p* = .017) and Hydrologists (*p* = .027).

Those in Medicine/Health Science and Business also expressed significantly lower agreement than other disciplines with the statement *I would be willing to share data across a broad group of researchers* (question 21.6). Medicine/Health Science researchers agreed with this statement significantly less than those in Atmospheric Science (*p* = .047), Biology (*p* = .043), Ecology (*p* = .001), Environmental Science (*p* = .001), Hydrology (*p* .008), Information Science (*p* = .005), and Physical Sciences (*p* = .047). Those in Business expressed significantly lower agreement than those in Ecology (*p* = .025), Environmental Science (*p* = .023), Hydrology (*p* = .026), and Information Science (*p* = .033). Those in Law also expressed significantly lower agreement than those in Hydrology (*p* = .045).

The importance of being cited also varied according to subject discipline. In response to the statement *it is important that my data are cited when used by other researchers* (question 21.7), those in Education expressed significantly less agreement than those in Ecology (*p* = .032), and those in Law expressed significantly less agreement than those in Biology (*p* = .045), Ecology (*p* = .039), and Hydrology (*p* = .032). Finally, when it comes to reuse of data, the statement *it is appropriate to create new datasets from shared data* (question 21.8) saw significant variation across subject disciplines with no significant pairwise differences.

The perceived risks associated with data sharing (question 20.3, 20.4, and 20.5), including the possibility that data may be *misinterpreted due to complexity of the data*, *misinterpreted due to poor quality of the data*, and *used in ways other than intended* did not vary significantly in agreement across the subject disciplines (see Table KK in S1 Appendix).

Among the barriers to making data available (question 13b), chi-square tests with adjusted standardized residuals indicated differences across subject discipline for four of the given barriers (using Monte Carlo test of significance). The proportion of respondents who selected *there is insufficient time to make them available* (question 13b.4) varied significantly, as did the idea that data *shouldn’t be available* (question 13b.6) to be shared, with Education (46.7%) Medicine/Health Science (29.6%), and Psychology (31.3%) all having significantly higher proportions of “yes” responses than the overall total. Specific results (see Table LL in S1 Appendix) are not surprising, given the private nature of human data collected in these fields.

Some disciplines were significantly more inclined to agree that they *don’t have the rights to make data public* (question 13b.8) including Education (73.3%) and Medicine/Health Sciences (59.3%). Biologists (65.2%) and Physical Scientists (61.3%) expressed more agreement than the overall total that they *need to publish first* (question 13b.10). This was significantly less of a barrier than expected for Computer Scientists (18.2%), Education researchers (13.3%), and Information Science researchers (23.5%).

Of the 12 conditions given for fair use of the respondents’ data (question 16), chi-square tests with adjusted standardized residuals demonstrated that all but two (*the data provider is given a complete list of all products that make use of the data*, *including articles*, *presentations*, *educational materials*, *etc*. and *the data provider is given and agrees to a statement of uses to which the data will be put*) varied significantly across subject disciplines in terms of whether respondents answered “yes,” “no,” or “not sure.” Requiring *co-authorship* (question 16.1), *acknowledgment in all disseminated work* (question 16.2), *citation of data providers on all disseminated work* (question 16.3), the opportunity to *collaborate on a project using the data* (question 16.4), *results could not be disseminated without the data provider’s approval* (question 16.5), *covering part of the costs* (question 16.6), the opportunity to *review the results and make suggestions or comments* (question 16.7), *providing reprints of articles* (question 16.8), obtaining *legal permission for data use* (question 16.10), and requiring *mutual agreement on reciprocal sharing of data* (question 16.11) are all conditions whose importance vary by subject disciplines (specific differences reported in Table MM in S1 Appendix).

We also examined differences in data sharing practices across subject disciplines. It is clear that perceptions about data sharing and reuse differ across the subject disciplines, and an ANOVA examining differences in the amount of data shared (question 13) across subject disciplines indicates that sharing practices differ significantly as well (see Table NN in S1 Appendix). No pairwise differences were significant. Differences in data sharing (question 15) are further confirmed by significant variation in response to questions about data sharing and access; specifically, *I share my data with others* (question 15.1), *others need permission to access my data* (question 15.2), and *others can access my data easily* (question 15.3). Tukey’s post hoc analyses reveal Psychology as a point of variation for *I share my data with others*, with it being was significantly lower than both Atmospheric Science and Ecology (see Table OO in S1 Appendix).

In terms of data storage (question 12), chi-square tests of independence using Monte Carlo test of significance showed significant differences across subject disciplines in five of the given deposit locations (see Table PP in S1 Appendix). Adjusted standardized residuals show those disciplines that differ significantly from the expected, or overall, total. For storage on a *departmental server* (question 12.3), Education (83.3%) and Engineering (70.4%) disciplines are significantly more likely to be storing data in these locations. Those in Computer Science (76.2%) and Education (78.6%) were significantly less likely to be storing data on their *personal computers* (question 12.4). A total of 92.8% of respondents to this question store at least some of their data on their own personal computer, making it the most common place to store data. A total of 65.5% of respondents store data *on paper* in their offices (question 12.5), a practice that also differed significantly by subject discipline. Those in Biology (81.1%) were more likely to store data on paper in their offices, while those in Atmospheric Science (41.7%), Computer Science (42.9%), and Information Science (50.0%) were less likely to do so.

It is perhaps not surprising that there are significant differences across the subject disciplines in terms of whether respondents store data in a *discipline-based repository* (question 12.6). A chi-square test using Monte Carlo test of significance showed that those from Ecology are more likely to store data in such a repository (44.6%), while those in Physical Sciences (7.1%) and Social Sciences (10.8%) were less likely to do so. Data storage in some *other data repository or archive* (question 12.8) also differs significantly by subject discipline, with a significantly higher proportion of those in Atmospheric Science (59.0%) responding “yes” than the total (31.9%).

The use of different metadata standards (question 8) is an important part of enhancing usability of shared data, and chi-square tests reveal that there are clear differences across subject disciplines in terms of how commonly each is used (see Table QQ in S1 Appendix). Perhaps most telling are the differences in actual versus expected proportions of those who selected “none” (question 8.12) from different subject disciplines. Lower percentages came from Atmospheric Science (28.6%), Ecology (40.6%), Environmental Science (36.7%), and Information Science (37.0%), implying more use of some metadata standard within these fields. Higher percentages of those who do not use any kind of metadata to describe their datasets came from those in Medicine/Health Science (64.9%), Physical Science (64.3%), Psychology (76.2%), Social Science (65.9%), and Humanities (87.5%), indicating less use of metadata standards in these disciplines.

Finally, there are no significant differences across subject disciplines in terms of how respondents perceive their organizations’ involvement in and support for data management (questions 17, 18, 19–1, and 19–2; see Table RR in S1 Appendix).

## Discussion

The approach by the NSF-sponsored DataONE project to re-assess the scientific community’s data sharing perceptions, practices, and related changes over the past 3 to 4 years has been illuminating, producing a number of both statistically and practically significant findings. Overall, favorable perceptions of and practices surrounding data sharing are increasing, albeit gradually. This shift varies according to researchers’ ages, geographic regions, and subject disciplines, indicating that perhaps data sharing is more normalized among some groups than others. This points to important implications for the promotion of data sharing and reuse, as well as a roadmap for which groups need to be targeted in these efforts.

### Baseline to Follow-Up Changes

Those in the follow-up study have responded more favorably to the idea of data sharing, and the level of actual engagement in data sharing and reuse has increased since the baseline. Yet equally important are findings that demonstrate where change has not occurred, or that results similar to those identified 4 years ago still exist today. Such findings tell us that there are ongoing, persistent data management challenges facing the scientific community.

Approaches to best practices in data management across disciplines and organizations are complex and often in contrast to one another, and may require years to change; therefore, the incongruence of these approaches continues to be an impediment to the complex science of today. Where the follow-up results remain unchanged, and if certain facets of data sharing continue to stagnate in the future, it is worth questioning the overall effectiveness and impact of current data management policies, plans, infrastructure, and related activities. It is possible that we have reached a tipping point where attitudes toward data sharing will remain the same in spite of whatever policies, plans, infrastructure, or practices are implemented. In addition, institutionalized practices related to tenure, promotion, and small-scale scientific collaboration may still be too dominant to change these perceptions and practices [11].

In the follow-up study, we found more agreement and willingness among scientists to share at least some or all of their data across broader groups with no limitations. At the same time, the importance of data citation is an ongoing issue, and is one that will likely accompany the gradual institutionalization of emerging scientific practices over the next few years. In contrast to scientists’ willingness to share data, results also show increases in scientists’ concern over data being misinterpreted due to the complexity of the data, poor quality of data, or data being used in ways other than its intended purpose. These concerns could potentially be alleviated by proper documentation or metadata; however, as with the baseline study, respondents in the follow-up study express a high-level of dissatisfaction with tools for preparing metadata. Clearly, further development of easier-to-use data management tools is needed in this area.

When it comes to making data available for others to use, a lack of both time and funding continue to be issues among researchers; however, these issues are not as prevalent they were 3 to 4 years ago. This change could be due to greater financial investment in data management by funding organizations, a better understanding of the value of data management and sharing by scientists and their organizations, or perhaps an acknowledgement that sharing data is a necessary part of the research lifecycle if we are going to be able to advance scientific research.

### Follow-Up Survey: Demographic Factors

#### Age

Overall, differences between the youngest and oldest age groups indicate that younger researchers think more favorably about data sharing and reuse, yet they prioritize control over and credit for their work more than older researchers do. Clearly, the pressure to publish results first is influencing perceptions. Those in the youngest age group find co-authorship and opportunities for collaboration to be a more important condition for use of their data than those 50 and older, and cite the need to publish their research first as a barrier to data sharing. They are more inclined to perceive that legal permission for use of their data is a reasonable condition, as well as the opportunity to review what others have produced with their data and make suggestions.

Those in the middle age cohort (40–49) fall fairly consistently between their older and younger colleagues when it comes to data sharing perceptions and practices, as well as in their perceptions of organizational support for data management. Although there are no items for which they express significantly highest or lowest mean agreement, there are a few non-significant items that may provide direction for understanding this important group of researchers. For instance, when it comes to willingness to engage in data sharing, those in the middle age cohort expressed slightly higher agreement that they would be willing to share data across a broad group of researchers, that it is important that their data are cited when used by others, and that it is appropriate to create new datasets from shared data than both the youngest and oldest cohorts, although these differences are all non-significant (see table 2.2). In addition, the middle cohort expresses slightly lower agreement for some items pertaining to perceived organizational support for data management, including support for long-term data management and storage, training or assistance on creating data management plans, and assistance on creating metadata, although results are non-significant (see table 2.10). In the future, research may benefit from examining age in a continuous, linear manner to better understand the nuances of this important age group.

Results related to age groups can in part be explained with a lifespan psychology approach. The need for recognition in the workplace (e.g., authorship) is negatively related to age, as are the needs for development, challenge, and advancement or promotion. On the other hand, the need for autonomy increases significantly with age [36]. This helps shed light on why older respondents felt less need or use for others’ data, while younger respondents leaned more toward collaboration, data sharing, receiving credit for and having control over their work.

Findings about age and perceptions about data sharing and reuse are especially interesting given the results about actual data sharing and reuse practices. Those 50 and older claim to share significantly more of their data than both the 40–49 and 22–39 age groups. In contrast, younger respondents have more restrictions on access to their data, and agreed significantly less than older respondents that their data is easy to access. Therefore, our examination of age reveals that there is a possible disconnect between perceptions data sharing/reuse and their actions when it comes to making their own data available.

Although the oldest respondents purport to be sharing significantly more of their data than the younger age groups, younger respondents profess more positive perceptions about the idea of data sharing. This could be due to the fact that as scientists are aging and leaving the workforce, they possess a strong desire to have their research “live on” beyond them. Effective ways to ensure the longevity of an individual’s research include proper documentation, broad data sharing, and advocating for a culture of open data. Such barriers as the need to publish and the desire for more credit and control over their work may prevent younger respondents from sharing their data. Unfortunately, these needs stem from a system of “publish or perish” that is unlikely to change within the current academic system. Perhaps young researchers can be encouraged to look back at older data sets from which they have already published, and be given the time and support to clean and deposit these for others to use. Data sharing incentives that provide the researcher with authorship or credit for use of older data may mitigate the problems associated with the pressure to publish novel findings.

#### Geographic location

When examining geographic location as a predictor of data sharing perceptions and behaviors, differences may be explained by cultural factors. Respondents from Asian countries, for example, felt more strongly about data access as an important part of their own scientific pursuits; however, they also agreed more strongly than those from other geographic regions that permission was needed to access their data. North American respondents, on the other hand, were more wary of possible misuse of shared data, and were also less likely than Asian participants to agree that conditions for use of their data were fair. When it comes to perceived organizational support, Asian participants were also more likely to agree that support for data management and the creation of metadata was present, while European respondents agreed significantly less.

Differences between and across continents can be examined through the lens of the individualism—collectivism spectrum. Because the individualist derives a sense of identity that is based on personal goals and values [37] and functions in a state of emotional independence from any group, organization, or collective [38], he or she many be less inclined to act in a way that puts the interests of a group ahead of self-interest. In collectivist cultures, a person’s sense of identity is closely tied to social systems instead of individual attributes [39].

Data sharing may be classified as a behavior that puts the self-interest of the individual scientist aside, instead capitalizing on a person’s effort to promote the advancement of a larger institution, discipline, field, or science in general [6,7,11]. It is possible, therefore, that results are reflecting cultural differences in perceptions and behaviors related to data sharing. For example, by agreeing more strongly that organizational support for data management activities is present, Asian participants may be “collectively” demonstrating loyalty to the organizations for which they work. North American participants, on the other hand, may be more risk-averse when it comes to data sharing, because they want to see a personal payoff for their individual efforts.

#### Subject discipline

Subject discipline played an interesting–yet perhaps unsurprising–role in respondents’ data sharing perceptions and behaviors. In particular, those in Medicine/Health Sciences and others who work with human subjects were significantly less willing to share their data than respondents other disciplines. This may be attributable to the sensitive nature of protected health information with which they work. Yet interestingly, there were no significant differences across subject disciplines when it came to perceived risks associated with data sharing. This could be because no risks related to violating the confidentiality of human subjects were posited. Social scientists expressed significantly less satisfaction with their ability to use others’ data to address their own research questions. Again, this may be because of the difficulty and risk associated with making human subjects’ data available.

In terms of the specific barriers to data sharing, it is not surprising that those from Education, Medicine/Health Science, and Psychology were more inclined than the overall total to agree that their data shouldn’t be available for others to use in the first place. Those in Education and Medicine/Health Science were also more inclined to agree that they don’t have the right to make their data available in the first place. Again, could be likely attributable to the fact that they, unlike those in Atmospheric Science, Ecology, or Physical Sciences, are working with human subjects’ data, and risk violating the confidentiality of those subjects.

## Conclusions and Recommendations

Overall, these studies provide a glimpse of the shifting nature of data sharing among research scientists. The data show that this is a complex shift, with varying cultures among scientists that dictate the norms surrounding data sharing and reuse. Changes from the baseline to the follow-up study indicate that not only is data sharing behavior increasing, but that researchers are also viewing the practice and the overall movement more favorably. There are still perceived risks and barriers that may be slowing the data sharing movement, however. One way these continued barriers can perhaps be mitigated is by further development of user-friendly data management tools incorporated into standard data collection and analysis tools and that help resolve the perceived barrier of lack of time for data sharing. Improving tools are not the only steps necessary to overcome barriers. The next steps will likely involve training for researchers, or the ready availability of well-trained data managers to assist with the extra tasks required to describe and share data.

Data sharing practices will be increased not only by making it easier to share data, but also by providing incentives to authors. Citation, co-authorship, and other means of bestowing credit for their work is clearly important to the researchers who collect, quantify, clean, and manage their original data. Building incentives into the data sharing process, particularly at the point of data reuse, must be adopted within corresponding data management policies. For instance, data repositories could have set rules about following citation standards to properly credit authors whose data is being utilized, as well as the data repository for where the data resides. At the same time, those in the academic sector who are being considered for tenure and promotion could be credited when they produce and share particularly useful and cited datasets. Citation and academic reward systems, in other words, could be rethought to make data sharing worthwhile.

In terms of the current state of data-sharing, demographic differences related to age, geographic region, and subject discipline provide important opportunities to strategically improve different groups’ data sharing practices and perceptions. Issues related to cultural and discipline-based differences tend to be more entrenched than those related to age. If it is an individualistic fear of the loss of research credit that makes certain cultures less inclined to share data, then the focus might be not on how to make these cultures adopt more collectivist attitudes, but on how to reward researchers from places like Australia, Western Europe, South America, and the United States for data sharing in ways that satisfy both individual and broader community needs. Overcoming data sharing issues related to human health and behavioral data may also be tricky. It is neither surprising nor blameworthy that scientists from disciplines that work primarily with human subjects are concerned about protecting those who are willing to share their private information with them. For those working with human subjects, perhaps the answer lies in publishing a more generalized or buffered dataset (broader scale), thus reducing the risk of large amounts of aggregate finer scale data rendering otherwise anonymous information identifiable.

Future research could examine the varying individual cultures within the scientific community more closely, using focused research questions to understand, for instance, how we can continue to build infrastructure that promotes data sharing given the needs of different research communities. We can also continue to develop data sharing models that reward these different research cultures according to their different needs. Another follow-up study is planned in the coming years by the DataONE organization to see how continued data sharing conversations, growth in funder and publisher mandates, and an increase in availability of data repositories will change practices and perceptions of data sharing and reuse.

Moving forward, it is worth questioning the value of open data and data sharing. Given the possibility of unanticipated challenges, concerns, and potential impacts to the time available for scholars to conduct actual scientific research, is open data always a good thing? The current results indicate, for instance, concerns among those who use human subjects data, as they express less willingness to engage in data sharing and sense greater barriers to making their data available. In addition, data quality issues can be difficult to monitor, as the process by which data were originally collected and managed may be uncertain. Therefore, in the interest of rigor, is it not sometimes better to collect new or additional data? These and other potential downsides of data sharing are important to consider as the open access movement progresses.

But ultimately, we agree with the baseline and follow-up respondents: scientists need access to data, and lack of data sharing can be a major impediment to progress in science. Over time, improved data sharing practices and perceptions will hopefully continue to increase until a data sharing culture is the norm across most sciences. This is why it is critical to continue to assess, monitor, educate, and provide the infrastructure necessary to support the complex grand science challenges facing the world today, which cannot be solved without effective, long-term, sustainable data management practices.

## Limitations

The limitations of this study stem from the fact that we are relying on a volunteer sample, from which self-selection bias may have occurred based on interest, familiarity, personal relevance, or favorable feelings toward the topic. Also, as a comparative study, the survey replies are anonymous, and the respondents in the baseline and follow-up are likely different people; therefore, we are comparing overall patterns rather than the responses of specific individuals. Although a control variable was used to account for differences in internationality between the two surveys, other differences remain between the two samples that make them difficult to compare. Web surveys tend to have low response rates compared to other modes of distribution [40] but due to reliance on others to distribute the survey invitation and link to their institutions, colleagues, and listservs, we do not know how many potential respondents received the invitation and cannot estimate a response rate.

## Supporting Information

S1 Appendix Tables. (DOCX)

S2 Appendix Survey Questionnaire. (DOCX)
