# Supplementary material for: Changes in Data Sharing and Data Reuse Practices and Perceptions among Scientists Worldwide
Source: PLoS One. 2015 Aug 26;10(8):e0134826. doi: 10.1371/journal.pone.0134826 (PMC4550246; doi:10.1371/journal.pone.0134826)
Supplement: S1 Appendix — (DOCX) [file pone.0134826.s001.docx]

**S1 Appendix: Tables**

Table A. Age Categories

|  | **Baseline** | **Follow-Up** | **Total** |
| --- | --- | --- | --- |
| 20 – 39 | 453  37.6% | 380  40.9% | 833  39.1% |
| 40 – 49 | 310  25.7% | 196  21.1% | 506  23.7% |
| 50 + | 442  36.7% | 352  37.9% | 794  37.2% |

Table shows frequencies and valid percentages for each age category within the baseline, follow-up, and combined datasets.

Table B. Continent where employed

|  | **Baseline** | **Follow-Up** | **Total** |
| --- | --- | --- | --- |
| North America | 959 (73.4%) | 592 (61.0%) | 1551 (68.1%) |
| Asia | 78 (6.0%) | 91 (9.4%) | 169 (7.4%) |
| Europe | 192 (14.7%) | 141 (14.5%) | 333 (14.6%) |
| Africa | 23 (1.8%) | 72 (7.4%) | 95 (4.2%) |
| South America | 32 (2.5%) | 55 (5.7%) | 87 (3.8%) |
| Australia/New Zealand | 22 (1.7%) | 20 (2.1%) | 42 (1.8%) |

Table shows frequencies and valid percentages for each continent within the baseline, follow-up, and combined datasets.

Table C. Primary Subject Discipline

|  | **Baseline** | **Follow-Up** | **Total** |
| --- | --- | --- | --- |
| Agriculture and Natural Resources | 15  1.1% | 83  8.4% | 98  4.3% |
| Atmospheric Science | 52  3.9% | 59  6.0% | 111  4.8% |
| Biology | 181  13.7% | 70  7.1% | 251  10.9% |
| Business | 8  0.6% | 14  1.4% | 22  1.0% |
| Computer Science | 24  1.8% | 33  3.4% | 57  2.5% |
| Ecology | 237  18.0% | 163  16.5% | 400  17.4% |
| Education | 39  3.0% | 22  2.2% | 61  2.6% |
| Engineering | 94  7.1% | 45  4.6% | 139  6.0% |
| Environmental Science | 198  15.0% | 133  13.5% | 331  14.4% |
| Forestry | 10  0.8% | N/A | 10  0.4% |
| Geography | 15  1.1% | N/A | 15  0.7% |
| Geology | 49  3.7% | 21  2.1% | 70  3.0% |
| Humanities | N/A | 12  1.2% | 12  0.5% |
| Hydrology | 38  2.9% | 40  4.1% | 78  3.4% |
| Information Science | 26  2.0% | 84  8.5% | 110  4.8% |
| Law | 3  0.2% | 4  0.4% | 7  0.3% |
| Mathematics | 9  0.7% | N/A | 9  0.4% |
| Medicine/Health Science | 31  2.4% | 37  3.8% | 68  3.0% |
| Physical Sciences | 71  5.4% | 47  4.8% | 118  5.1% |
| Psychology | 23  1.7% | 21  2.1% | 44  1.9% |
| Social Sciences | 105  8.0% | 44  4.5% | 149  6.5% |
| Other | 89  6.8% | 53  5.4% | 142  6.2% |

Table shows frequencies and valid percentages for each subject discipline within the baseline, follow-up, and combined datasets.

Table D. Value of data sharing and reuse

|  | **Baseline** | | **Follow-Up** | | **ANOVA** |
| --- | --- | --- | --- | --- | --- |
|  | *M* | SD | *M* | SD | **F; *p*** |
| Lack of access to data generated by other researchers is a major impediment to progress in science. | 3.75 | 1.09 | 3.99 | 1.03 | F= 17.40  *p <* .001 |
| Lack of access to data generated by other researchers has restricted my ability to answer scientific questions. | 3.32 | 1.21 | 3.36 | 1.27 | F= .825  *p* = .362 |

Table shows mean agreement (1= Disagree strongly, 2= disagree somewhat, 3= neither agree nor disagree, 4= agree somewhat, 5= agree strongly) and standard deviation for each item. MANOVA: F(2, 1875) = 12.82, *p* < .001; Wilks’ Lambda= .987; partial eta squared= .013. Univariate ANOVAs for each item within omnibus MANOVA, controlling for North American vs. non-North American work locale (“NAvsNonNA”).

Table E. Willingness to engage in data sharing and reuse

|  | **Baseline** | | **Follow-Up** | |  |
| --- | --- | --- | --- | --- | --- |
|  | ***M*** | **SD** | ***M*** | **SD** | **F*; p*** |
| I would use other researchers’ datasets if their datasets were easily accessible. | 4.19 | 0.89 | 4.33 | 0.79 | F= 8.90  p = .003 |
| I would be willing to place at least some of my data into a central repository with no restrictions. | 4.06 | 1.06 | 4.29 | 1.00 | F= 17.51  p < .001 |
| I would be willing to place all of my data into a central repository with no restrictions. | 2.98 | 1.32 | 3.23 | 1.41 | F= 11.89  p = .001 |
| I would be more likely to make my data available if I could place conditions on access. | 3.71 | 1.09 | 3.54 | 1.21 | F= 9.21  p= .002 |
| I am satisfied with my ability to integrate data from disparate sources to address research questions. | 3.23 | 1.08 | 3.19 | 1.22 | F= 0.86  p= .355 |
| I would be willing to share data across a broad group of researchers. | 4.12 | 0.86 | 4.39 | 0.80 | F= 38.78  p < .001 |
| It is important that my data are cited when used by other researchers. | 4.58 | 0.72 | 4.49 | 0.80 | F= 6.01  p= .014 |
| It is appropriate to create new datasets from shared data. | 4.09 | 0.91 | 4.23 | 0.91 | F= 9.10  p = .003 |

Table shows mean agreement (1= Disagree strongly, 2= disagree somewhat, 3= neither agree nor disagree, 4= agree somewhat, 5= agree strongly) and standard deviation for each item. MANOVA: F(8, 1723) = 8.19, *p* < .001; Wilks’ Lambda= .963; partial eta squared= .037. Univariate ANOVAs for each item within omnibus MANOVA, controlling for North American vs. non-North American work locale (“NAvsNonNA”).

Table F. Perceived risks of data sharing and reuse

|  | **Baseline** | | | **Follow-Up** | |  |
| --- | --- | --- | --- | --- | --- | --- |
|  | ***M*** | **SD** | | ***M*** | **SD** | **F*; p*** |
| Data may be misinterpreted due to complexity of the data. | 3.96 | | 0.94 | 4.12 | 0.93 | F= 14.09  p < .001 |
| Data may be misinterpreted due to poor quality of the data. | 3.87 | | 1.01 | 4.19 | 0.88 | F= 44.18  p < .001 |
| Data may be used in ways other than intended. | 3.97 | | 0.94 | 4.21 | 0.87 | F= 30.02  p < .001 |

Table shows mean agreement (1= Disagree strongly, 2= disagree somewhat, 3= neither agree nor disagree, 4= agree somewhat, 5= agree strongly) and standard deviation for each item. MANOVA: F(3, 1846) = 17.41, *p* < .001, Wilks’ Lambda= .972, partial eta squared= .028. Univariate ANOVAs for each item within omnibus MANOVA, controlling for North American vs. non-North American work locale (“NAvsNonNA”).

Table G. Barriers to sharing data

|  | **Baseline** | **Follow-Up** | **Total** | ***X^2^; p*** |
| --- | --- | --- | --- | --- |
| Lack of funding | 33.5% | 24.6% | 30.5% | *X^2^* = 16.46  p < .001 |
| Lack of standards | 16.7% | 17.9% | 17.1% | *X^2^* = .379  p = .538 |
| People don’t need them | 12.7% | 24.7% | 16.8% | *X^2^* = 45.99  p < .000 |
| There is insufficient time to make them available | 45.4% | 38.6% | 43.1% | *X^2^* = 8.26  p = .004 |
| There is no place to put them | 19.9% | 18.2% | 19.3% | *X^2^* = .707  p = .400 |
| They shouldn't be available | 12.2% | 13.1% | 12.5% | *X^2^* = .265  p = .607 |
| Sponsor doesn't require it | 14.7% | 15.1% | 14.9% | *X^2^* = .028  p = .867 |
| Don't have the rights to make the data public | 20.4% | 26.1% | 22.3% | *X^2^* = 8.06  p = .005 |
| I would lose control of the data | N/A | 10.2% | N/A | N/A |
| I need to publish first | N/A | 43.5% | N/A | N/A |
| I have insufficient skills to make my data available | N/A | 13.2% | N/A | N/A |

Table shows percentages for each chosen barrier (“yes”) within baseline, follow-up, and overall combined. Chi-square tests (continuity correction) and probability values for each barrier given in both the baseline and follow-up surveys.

Table H. Conditions for use of subjects’ data (Follow-Up only)

|  | **Yes** | **No** | **Not Sure** |
| --- | --- | --- | --- |
| Co-authorship on publications resulting from use of the data. | 36.2% | 33.8% | 30.0% |
| Acknowledgement of the data providers in all disseminated work making use of the data. | 87.7% | 5.5% | 6.8% |
| Citation of the data providers in all disseminated work making use of the data. | 85.1% | 6.4% | 8.5% |
| The opportunity to collaborate on a project using the data. | 58.7% | 19.7% | 21.7% |
| Results based (at least in part) on the data could not be disseminated in any format without the data provider’s approval. | 29.0% | 50.8% | 20.2% |
| At least part of the costs of data acquisition, retrieval, or provision must be recovered. | 14.2% | 62.7% | 23.2% |
| Results based (at least in part) on the data could not be disseminated without the data provider having the opportunity to review the results and make suggestions or comments, but approval not required. | 36.9% | 42.5% | 20.6% |
| Reprints of articles that make use of the data must be provided to the data provider. | 46.8% | 38.6% | 14.6% |
| The data provider is given a complete list of all products that make use of the data, including articles, presentations, educational materials, etc. | 43.8% | 37.5% | 18.7% |
| Legal permission for data use is obtained. | 33.4% | 45.4% | 21.1% |
| Mutual agreement on reciprocal sharing of data. | 46.2% | 34.2% | 19.7% |
| The data provider is given and agrees to a statement of uses to which the data will be put. | 44.2% | 32.5% | 23.3% |

Table shows frequencies and percentages of those who selected yes, no, or not sure to each proposed condition for the follow-up results only.

Table I. Amount of data made available to others (Follow-Up only)

| **None** | 9.5% |
| --- | --- |
| **Some** | 43.1% |
| **Most** | 31.8% |
| **All** | 15.6% |

Table shows frequencies and percentages of those who selected none, some, most, or all for the follow-up results only.

Table J. Data accessibility

|  | **Baseline** | | | **Follow-Up** | |  |
| --- | --- | --- | --- | --- | --- | --- |
|  | ***M*** | **SD** | | ***M*** | **SD** | **F; *p*** |
| I share my data with others. | 3.96 | | 0.99 | 4.06 | 1.10 | F= 6.76  p = .009 |
| Others can access my data easily. | 2.92 | | 1.26 | 3.15 | 1.43 | F= 13.98  p < .001 |

Table shows mean agreement (1= Disagree strongly, 2= disagree somewhat, 3= neither agree nor disagree, 4= agree somewhat, 5= agree strongly) and standard deviation for each item. MANOVA: F(2, 1977) = 7.14, *p <* .001, Wilks’ Lambda= .993, partial eta squared= .007. Univariate ANOVAs for each item within omnibus MANOVA, controlling for North American vs. non-North American work locale (“NAvsNonNA”).

Table K. Data storage locations for follow-up only

|  | **None** | **Some** | **Most** | **All** | **Mean**  **(1-4)** |
| --- | --- | --- | --- | --- | --- |
| On my institution’s server | 31.9% | 30.4% | 19.4% | 18.3% | 2.24 |
| On the PI’s server | 47.0% | 24.5% | 12.4% | 16.0% | 1.98 |
| On a departmental server | 51.5% | 25.4% | 12.6% | 10.5% | 1.82 |
| On my personal computer | 7.2% | 27.5% | 24.8% | 40.5% | 2.99 |
| On paper in my office | 34.5% | 51.9% | 6.6% | 7.1% | 1.86 |
| In a discipline-based repository | 72.5% | 18.0% | 7.3% | 2.2% | 1.39 |
| In a publisher or publisher-related repository | 80.6% | 16.9% | 1.9% | 0.5% | 1.22 |
| Other data repository or archive | 68.1% | 22.6% | 5.6% | 3.7% | 1.45 |
| In my institution’s repository | 67.2% | 21.5% | 6.6% | 4.7% | 1.49 |
| Other | 78.8% | 10.9% | 6.1% | 4.2% | 1.36 |
| Dropbox/Google/  Figshare/Cloud | 0.0% | 42.9% | 28.6% | 28.6% | 2.86 |
| External Hard Disk/Drive Storage | 0.0% | 16.7% | 33.3% | 50.0% | 3.33 |
| Other Server | 0.0% | 25.0% | 25.0% | 50.0% | 3.25 |

Table includes percentages and means (1= “none,” 2= “some,” 3= “most,” 4 = “all”) for each storage location.

Table L. Metadata standards used

|  | **Baseline** | **Follow-Up** | **Total** | ***X^2^; p*** |
| --- | --- | --- | --- | --- |
| DC (Dublin Core) | 2.0% | 7.1% | 4.1% | *X^2^ =* 35.77  p < .001 |
| DwC (Darwin Core) | 1.6% | 2.0% | 1.8% | *X^2^ =* .448  p = .503 |
| DIF (Directory Interchange Format) | 0.9% | 1.7% | 1.2% | *X^2^ =* 2.38  p = .123 |
| EML (Ecological Metadata Language) | 7.1% | 9.3% | 8.0% | *X^2^ =* 3.07  p = .080 |
| FGDC (Federal Geographic Data Committee) | 7.1% | 8.5% | 7.7% | *X^2^ =* 1.26  p = .261 |
| ISO 19115 (Geographic Information-Metadata) | 7.3% | 10.2% | 8.5% | *X^2^ =* 5.73  p = .017 |
| Other ISO metadata standard | 5.6% | N/A | N/A | N/A |
| OGIS (Open GIS) | 7.2% | 7.2% | 7.2% | *X^2^ =* .000  p = 1.00 |
| ANZLIC metadata profile | 0.2% | N/A | N/A | N/A |
| metadata standardized within my institution | 14.0% | N/A | N/A | N/A |
| metadata standardized within my lab | 20.0% | 16.7% | 18.7% | *X^2^ =* 3.74  p = .053 |
| None | 50.9% | 47.9% | 49.6% | *X^2^ =* 1.79  p = .181 |
| Other | 6.2% | 8.6% | 7.2% | *X^2^ =* 4.60  p = .032 |

Table shows percentages for each chosen metadata standard (“yes”) within baseline, follow-up, and overall combined results. Results of Chi-square tests (continuity correction) for each metadata standard given in both the baseline and follow-up surveys.

Table M. Satisfaction with data practices

|  | **Baseline** | | | **Follow-Up** | | **ANOVA** |
| --- | --- | --- | --- | --- | --- | --- |
|  | ***M*** | **SD** | | ***M*** | **SD** | **F; *p*** |
| Process for collecting my research data | 4.01 | | 0.92 | 4.05 | 0.94 | F = 1.28  p = .258 |
| Process for searching for my own data | 3.81 | | 0.95 | 3.43 | 1.22 | F = 47.47  p < .001 |
| Process for cataloging/describing my data | 3.56 | | 1.05 | 3.52 | 1.14 | F = .403  p = .526 |
| Process for storing data during life of the project (short-term) | 3.88 | | 0.99 | 3.77 | 1.11 | F = 3.59  p = .058 |
| Process for storing data during life of the project (long-term) | 3.16 | | 1.22 | 3.03 | 1.31 | F = 4.63  p = .032 |
| Process for analyzing my data | 3.93 | | 0.94 | 3.94 | 1.05 | F = .052  p = .820 |
| Tools for preparing metadata | 2.90 | | 1.01 | 2.87 | 1.22 | F = .526  p = .469 |
| Tools for preparing documentation | 3.26 | | 1.06 | 3.11 | 1.21 | F = 8.27  p = .044 |

Table shows mean agreement (1= Disagree strongly, 2= disagree somewhat, 3= neither agree nor disagree, 4= agree somewhat, 5= agree strongly) and standard deviation for each item. MANOVA: F(8, 1693) = 12.05, *p* < .001, Wilkes Lambda= .946, partial eta squared= .054. Univariate ANOVAs for each item within omnibus MANOVA, controlling for North American vs. non-North American work locale (“NAvsNonNA”).

Table N. Perceptions of organizational support for data management and policies

|  | **Baseline** | | | **Follow-Up** | | **ANOVA** |
| --- | --- | --- | --- | --- | --- | --- |
|  | ***M*** | **SD** | | ***M*** | **SD** | **F; *p*** |
| Managing data during the life of the project (short term) | 2.92 | | 1.44 | 2.80 | 1.51 | F = 2.72  p = .099 |
| Managing and storing data beyond life of project (long-term) | 2.81 | | 1.44 | 2.76 | 1.52 | F = .419  p = .517 |
| Provides training on best practices for data management | 2.36 | | 1.24 | 2.51 | 1.39 | F = 4.05  p = .044 |
| Provides the necessary funds to support data management during the life of a research project (short-term) | 2.66 | | 1.30 | 2.85 | 1.51 | F = 8.02  p = .005 |
| Provides the necessary funds to support data management beyond the life of a research project (long-term) | 2.35 | | 1.27 | 2.42 | 1.44 | F = .968  p = .325 |
| Provides the necessary tools and technical support for data management during the life of a research project (short-term) | 3.07 | | 1.33 | 3.04 | 1.46 | F = .037  p = .848 |
| Provides the necessary tools and technical support for data management beyond the life of a research project (long-term) | 2.76 | | 1.36 | 2.62 | 1.44 | F = 3.27  p = .071 |

Table shows mean agreement (1= Disagree strongly, 2= disagree somewhat, 3= neither agree nor disagree, 4= agree somewhat, 5= agree strongly) and standard deviation for each item. MANOVA: F(7, 1688) = 6.15, *p* < .001, Wilks’ Lambda= .975, partial eta squared= .025. Univariate ANOVAs for each item within omnibus MANOVA, controlling for North American vs. non-North American work locale (“NAvsNonNA”).

Table O. Value of data sharing and reuse by age group

| **Age** | **22-39** | | **40-49** | | **50 +** | | **ANOVA** |
| --- | --- | --- | --- | --- | --- | --- | --- |
|  | *M* | SD | *M* | SD | *M* | SD | **F; *p*** |
| Lack of access to data generated by other researchers is a major impediment to progress in science. | 4.14_c_ | 0.94 | 4.02 | 1.08 | 3.85_a_ | 1.06 | F = 4.73  p = .009 |
| Lack of access to data generated by other researchers has restricted my ability to answer scientific questions. | 3.60_c_ | 1.21 | 3.40 | 1.21 | 3.18_a_ | 1.27 | F = 6.74  p = .001 |

Table shows mean agreement (1= Disagree strongly, 2= disagree somewhat, 3= neither agree nor disagree, 4= agree somewhat, 5= agree strongly) and standard deviation for each item. MANOVA: F(4, 1184) = 3.52, *p* = .007, Wilks’ Lambda= .977, partial eta squared= .012.

Univariate ANOVAs for each item within omnibus MANOVA.

*_a_ = Tukey’s post-hoc analysis, differs significantly from 22-39*

*_c_ = Tukey’s post-hoc analysis, differs significantly from 50+*

Table P. Willingness to engage in data sharing and reuse by age group

| **Age** | **22-39** | | **40-49** | | **50 +** | | **ANOVA** |
| --- | --- | --- | --- | --- | --- | --- | --- |
|  | *M* | SD | *M* | SD | *M* | SD | **F*; p*** |
| I would use other researchers’ datasets if their datasets were easily accessible. | 4.47_c_ | 0.70 | 4.27 | 0.89 | 4.23_a_ | 0.83 | F = 4.59  p = .011 |
| I would be willing to place at least some of my data into a central repository with no restrictions. | 4.26 | 1.01 | 4.24 | 0.92 | 4.30 | 1.05 | F = .119  p = .888 |
| I would be willing to place all of my data into a central repository with no restrictions. | 3.16 | 1.39 | 3.19 | 1.38 | 3.31 | 1.46 | F = .623  p = .537 |
| I would be more likely to make my data available if I could place conditions on access. | 3.76_c_ | 1.17 | 3.45 | 1.16 | 3.37_a_ | 1.26 | F = 5.45  p = .005 |
| I am satisfied with my ability to integrate data from disparate sources to address research questions. | 3.29 | 1.26 | 3.22 | 1.17 | 3.07 | 1.20 | F = 1.74  p = .176 |
| I would be willing to share data across a broad group of researchers. | 4.40 | 0.76 | 4.42 | 0.70 | 4.36 | 0.87 | F = .224  p = .800 |
| It is important that my data are cited when used by other researchers. | 4.53 | 0.71 | 4.57 | 0.77 | 4.46 | 0.83 | F = .893  p = .410 |
| It is appropriate to create new datasets from shared data. | 4.24 | 0.90 | 4.27 | 0.88 | 4.19 | 0.97 | F = .286  p = .751 |

Table shows mean agreement (1= Disagree strongly, 2= disagree somewhat, 3= neither agree nor disagree, 4= agree somewhat, 5= agree strongly) and standard deviation for each item. MANOVA: F(16, 1006) = 1.67, *p* = .047, Wilks’ Lambda= .949, partial eta squared= .026.

Univariate ANOVAs for each item within omnibus MANOVA.

*_a_ = Tukey’s post-hoc analysis, differs significantly from 22-39*

*_c_ = Tukey’s post-hoc analysis, differs significantly from 50+*

Table Q. Perceived risks of data sharing and reuse by age group

| **Age** | **22-39** | | **40-49** | | **50 +** | | **ANOVA** |
| --- | --- | --- | --- | --- | --- | --- | --- |
|  | *M* | SD | *M* | SD | *M* | SD | **F; *p*** |
| Data may be misinterpreted due to complexity of the data. | 4.25 | 0.87 | 4.01 | 0.89 | 4.05 | 1.00 | F = 3.64  p = .027 |
| Data may be misinterpreted due to poor quality of the data. | 4.25 | 0.90 | 4.17 | 0.85 | 4.13 | 0.92 | F = 1.02  p = .363 |
| Data may be used in ways other than intended. | 4.21 | 0.88 | 4.21 | 0.86 | 4.19 | 0.91 | F = .029  p = .972 |

Table shows mean agreement (1= Disagree strongly, 2= disagree somewhat, 3= neither agree nor disagree, 4= agree somewhat, 5= agree strongly) and standard deviation for each item. MANOVA: F(6, 1156) = 1.44, *p* = .196, Wilks’ Lambda= .985, partial eta squared= .007.

Univariate ANOVAs for each item within omnibus MANOVA.

Table R. Barriers to sharing data by age group

| **Age** | **22-39** | **40-49** | **50 +** | **Total** | ***X^2^; p*** |
| --- | --- | --- | --- | --- | --- |
| Lack of funding | 20.1% (-2.3)* | 23.3%  (-0.4) | 30.7%  (2.7)* | 24.6% | *X^2^* = 7.90  P = .019 |
| Lack of standards | 18.7%  (0.2) | 21.2%  (1.0) | 16.4%  (-1.0) | 18.4% | *X^2^* = 1.44  P= .487 |
| People don’t need them | 21.6%  (-1.7) | 30.1%  (1.7) | 25.6%  (0.3) | 24.9% | *X^2^* = 3.90  P .142 |
| There is insufficient time to make them available | 33.6%  (-2.2) | 41.8%  (1.0) | 42.0%  (1.4) | 38.4% | *X^2^* = 4.82  P = .090 |
| There is no place to put them | 18.7%  (0.4) | 16.4%  (-0.6) | 18.1%  (0.0) | 18.0% | *X^2^* = .344  P = .842 |
| They shouldn't be available | 13.4%  (0.4) | 11.6%  (-0.5) | 13.0%  (0.1) | 12.9% | *X^2^* = .279  P = .870 |
| Sponsor doesn't require it | 15.2%  (-0.1) | 14.4%  (-0.3) | 16.0%  (0.4) | 15.3% | *X^2^* = .179  P= .915 |
| Don't have the rights to make the data public | 27.2%  (0.6) | 23.3%  (-0.9) | 26.5%  (0.2) | 26.1% | *X^2^* = .796  P= .672 |
| I would lose control of the data | 10.2%  (-0.1) | 6.8%  (-1.6) | 12.6%  (1.4) | 10.3% | *X^2^* = 3.24  P = .198 |
| I need to publish first | 50.9%  (3.0)* | 44.5%  (0.1) | 35.7%  (-3.2) | 44.1% | *X^2^* = 12.08  P = .002 |
| I have insufficient skills to make my data available | 13.4%  (0.0) | 15.1%  (0.6) | 12.6%  (-0.5) | 13.5% | *X^2^* = .472  P = .790 |

Table shows percentages for each chosen barrier (“yes”) within each age group. Chi-square tests for each metadata standard are reported. Adjusted standardized residuals are reported beneath each percentage, with those greater than 2.0 and less than -2.0 indicating significant deviation from expected cell values (the total). Chi-square values reported in results.

** Significant difference from expected value*

Table S. Conditions for use of your data by age group

| **Age** | **22-39** | | **40-49** | | **50 +** | | **Total** | | ***X^2^; p*** |
| --- | --- | --- | --- | --- | --- | --- | --- | --- | --- |
| Co-authorship on publications resulting from use of the data. | **yes** | 42.7%  (2.7)* | **yes** | 35.7%  (-0.2) | **yes** | 30.8%  (-2.5)* | **yes** | 36.4% | *X^2^*= 10.66  P=.031 |
|  | **no** | 29.6%  (-1.9) | **no** | 30.7%  (-0.9) | **no** | 39.5%  (2.6)* | **no** | 33.9% |  |
|  | **n/s** | 27.7%  (-0.9) | **n/s** | 33.6%  (1.1) | **n/s** | 29.7%  (0.0) | **n/s** | 29.7% |  |
| Acknowledgement of the data providers in all disseminated work making use of the data. | **yes** | 85.4%  (-1.5) | **yes** | 92.8%  (2.0) | **yes** | 87.6%  (-0.1) | **yes** | 87.8% | *X^2^*= 4.74  P= .316 |
|  | **no** | 6.5%  (1.1) | **no** | 2.9%  (-1.4) | **no** | 5.5%  (0.1) | **no** | 5.3% |  |
|  | **n/s** | 8.1%  (1.0) | **n/s** | 4.3%  (-1.3) | **n/s** | 6.9%  (0.1) | **n/s** | 6.8% |  |
| Citation of the data providers in all disseminated work making use of the data. | **yes** | 87.0%  (0.9) | **yes** | 87.9%  (1.0) | **yes** | 82.6%  (-1.7) | **yes** | 85.4% | *X^2^*= 4.69  P= .321 |
|  | **no** | 5.0%  (-1.2) | **no** | 4.3%  (-1.1) | **no** | 8.7%  (2.1) | **no** | 6.3% |  |
|  | **n/s** | 8.0%  (-0.2) | **n/s** | 7.8%  (-0.2) | **n/s** | 8.7%  (0.3) | **n/s** | 8.3% |  |
| The opportunity to collaborate on a project using the data. | **yes** | 66.8%  (3.3)* | **yes** | 62.7%  (1.0) | **yes** | 49.5%  (-4.1)* | **yes** | 58.9% | *X^2^*= 18.04  P= .001 |
|  | **no** | 15.3%  (-2.3)* | **no** | 17.6%  (-0.7) | **no** | 25.3%  (2.9)* | **no** | 19.8% |  |
|  | **n/s** | 17.9%  (-1.7) | **n/s** | 19.7%  (-0.5) | **n/s** | 25.3%  (2.1)* | **n/s** | 21.3% |  |
| Results based (at least in part) on the data could not be disseminated in any format without the data provider’s approval. | **yes** | 30.5%  (0.5) | **yes** | 29.3%  (0.0) | **yes** | 28.5%  (-0.4) | **yes** | 29.4% | *X^2^*= 7.01  P= .135 |
|  | **no** | 45.9%  (-1.8) | **no** | 47.9%  (-0.6) | **no** | 55.5%  (2.3)* | **no** | 50.2% |  |
|  | **n/s** | 23.6%  (1.6) | **n/s** | 22.9%  (0.8) | **n/s** | 16.1%  (-2.3)* | **n/s** | 20.4% |  |
| At least part of the costs of data acquisition, retrieval, or provision must be recovered. | **yes** | 14.3%  (0.4) | **yes** | 13.7%  (0.0) | **yes** | 13.1%  (-0.4) | **yes** | 13.7% | *X^2^*= 1.69  P= .792 |
|  | **no** | 62.8%  (0.0) | **no** | 59.0%  (-1.0) | **no** | 64.6%  (0.8) | **no** | 62.7% |  |
|  | **n/s** | 22.9%  (-0.3) | **n/s** | 27.3%  (1.2) | **n/s** | 22.3%  (-0.7) | **n/s** | 23.5% |  |
| Results based (at least in part) on the data could not be disseminated without the data provider having the opportunity to review the results and make suggestions or comments, but approval not required. | **yes** | 43.6%  (2.9)* | **yes** | 33.3%  (-0.9) | **yes** | 31.9%  (-2.1)* | **yes** | 36.6% | *X^2^*= 13.51  P= .009 |
|  | **no** | 35.0%  (-3.0) | **no** | 41.8%  (-0.1) | **no** | 49.3%  (3.1)* | **no** | 42.3% |  |
|  | **n/s** | 21.4%  (0.2) | **n/s** | 24.8%  (1.2) | **n/s** | 18.8%  (-1.2) | **n/s** | 21.1% |  |
| Reprints of articles that make use of the data must be provided to the data provider. | **yes** | 46.1%  (-0.4) | **yes** | 48.2%  (0.3) | **yes** | 47.4%  (0.2) | **yes** | 47.1% | *X^2^*=6.47  P= .167 |
|  | **no** | 39.9%  (0.6) | **no** | 31.7%  (-1.8) | **no** | 40.5%  (0.9) | **no** | 38.5% |  |
|  | **n/s** | 14.0%  (-0.3) | **n/s** | 20.1%  (2.1) | **n/s** | 12.0%  (-1.5) | **n/s** | 14.5% |  |
| The data provider is given a complete list of all products that make use of the data, including articles, presentations, educational materials, etc. | **yes** | 48.5%  (1.9) | **yes** | 46.8%  (0.8) | **yes** | 38.2%  (-2.5) | **yes** | 43.9% | *X^2^*= 8.13  P= .087 |
|  | **no** | 34.2%  (-1.2) | **no** | 31.9%  (-1.4) | **no** | 42.5%  (2.4) | **no** | 37.1% |  |
|  | **n/s** | 34.2%  (-1.2) | **n/s** | 31.9%  (-1.4) | **n/s** | 42.5%  (2.4) | **n/s** | 37.1% |  |
| Legal permission for data use is obtained. | **yes** | 38.9%  (2.5)* | **yes** | 39.6%  (1.8) | **yes** | 24.7%  (-3.9)* | **yes** | 33.2% | *X^2^*= 23.34  P <.001 |
|  | **no** | 36.6%  (-3.6)* | **no** | 41.0%  (-1.1) | **no** | 55.6%  (4.5)* | **no** | 45.3% |  |
|  | **n/s** | 24.5%  (1.5) | **n/s** | 19.4%  (-0.7) | **n/s** | 19.6%  (-1.0) | **n/s** | 21.5% |  |
| Mutual agreement on reciprocal sharing of data. | **yes** | 53.1%  (2.6) | **yes** | 45.0%  (-0.4) | **yes** | 41.4%  (-2.3) | **yes** | 46.6% | *X^2^*= 8.67  P= .070 |
|  | **no** | 29.1%  (-2.1) | **no** | 32.9%  (-0.3) | **no** | 38.8%  (2.3) | **no** | 33.8% |  |
|  | **n/s** | 17.8%  (-0.9) | **n/s** | 22.1%  (0.9) | **n/s** | 19.8%  (0.1) | **n/s** | 19.5% |  |
| The data provider is given and agrees to a statement of uses to which the data will be put. | **yes** | 48.0%  (1.6) | **yes** | 47.5%  (0.9) | **yes** | 39.1%  (-2.2) | **yes** | 44.3% | *X^2^*= 9.25  P= .055 |
|  | **no** | 28.1%  (-1.7) | **no** | 26.6%  (-1.5) | **no** | 38.4%  (3.0) | **no** | 32.0% |  |
|  | **n/s** | 23.8%  (0.1) | **n/s** | 25.9%  (0.7) | **n/s** | 22.5%  (-0.6) | **n/s** | 23.7% |  |

Table shows percentages for each condition (“yes”) within each age group. Chi-square tests for each condition are reported. Adjusted standardized residuals are reported beneath each percentage, with those greater than 2.0 and less than -2.0 indicating significant deviation from expected cell values (the total).

** Significant difference from expected value*

Table T. Amount of data made available to others by age group

| **Age** | **22-39** | | **40-49** | | **50 +** | |
| --- | --- | --- | --- | --- | --- | --- |
|  | ***M*** | **SD** | ***M*** | **SD** | ***M*** | **SD** |
| How much of your data do you make available to others? | 2.33 | 0.81 | 2.52 | 0.76 | 2.74 | 0.92 |

Table shows mean agreement and standard deviation for each age group (1= None, 2= Some, 3= Most, 4= All). ANOVA: F(2, 803)= 19.36, *p* < .001

Table U. Data accessibility by age group

| **Age** | **22-39** | | **40-49** | | **50 +** | | **ANOVA** |
| --- | --- | --- | --- | --- | --- | --- | --- |
|  | ***M*** | **SD** | ***M*** | **SD** | ***M*** | **SD** | **F; *p*** |
| I share my data with others. | 3.86_c_ | 1.12 | 4.05 | 1.03 | 4.18_a_ | 1.01 | F= 6.20  P= .002 |
| Others need permission to access my data. | 3.80_c_ | 1.22 | 3.59 | 1.30 | 3.42_a_ | 1.40 | F= 5.70  P= .004 |
| Others can easily access my data. | 2.89_c_ | 1.34 | 3.11 | 1.34 | 3.19_a_ | 1.37 | F= 3.45  P= .032 |

Table shows mean agreement (1= Disagree strongly, 2= disagree somewhat, 3= neither agree nor disagree, 4= agree somewhat, 5= agree strongly) and standard deviation for each item. MANOVA: F(6, 1360) = 3.01, *p* = .006, Wilks’ Lambda= .974, partial eta squared= .013.

Univariate ANOVAs for each item within omnibus MANOVA.

*_a_ = Tukey’s post-hoc analysis, differs significantly from 22-39*

*_c_ = Tukey’s post-hoc analysis, differs significantly from 50+*

Table V. Data storage in different locations by age group (excludes Other, Dropbox/Google/Figshare/Cloud, External Hard Disk/Drive Storage, and Other Server because those answers were derived from text included with “other; please specify”)

| **Age** | **22-39** | **40-49** | **50+** | **Total** | ***X^2^; p*** |
| --- | --- | --- | --- | --- | --- |
| On my institution’s server | 63.8%  (-2.1) | 71.1%  (0.8) | 71.4%  (1.4) | 68.2% | *X^2^* = 4.27  P= .118 |
| On the principal investigator’s server | 50.0%  (-1.3) | 55.6%  (0.7) | 55.1%  (0.8) | 53.0% | *X^2^* = 1.64  P= .441 |
| On a departmental server | 49.6%  (0.3) | 47.1%  (-0.5) | 49.1%  (0.1) | 48.8% | *X^2^* = .232  P= .891 |
| On my personal computer | 94.0%  (0.7) | 92.1%  (-0.6) | 92.9%  (-0.2) | 93.2% | *X^2^* = .588  P= .745 |
| On paper in my office | 62.5%  (-1.2) | 61.4%  (-1.0) | 70.4%  (2.1) | 65.1% | *X^2^* = 4.36  P= .113 |
| In a discipline-based repository (e.g., NEON or LTER) | 25.8%  (-0.8) | 26.2%  (-0.4) | 30.4%  (1.2) | 27.6% | *X^2^* = 1.35  P= .510 |
| In a publisher or publisher-related repository (e.g., specific publisher or Dryad) | 18.4%  (-0.4) | 21.2%  (0.6) | 18.9%  (-0.1) | 19.2% | *X^2^* = .399  P= .819 |
| Other data repository or archive (e.g., national data center) | 26.7%  (-2.3) | 33.3%  (0.4) | 36.9%  (2.0) | 31.9% | *X^2^* = 5.54  P= .063 |
| In my institution’s repository | 32.4%  (0.0) | 33.9%  (0.4) | 31.7%  (-0.3) | 32.4% | *X^2^* = .169  P= .919 |

Table shows percentages for each storage location (“yes” within dichotomized variable) within each age group. Chi-square tests for each location are reported. Adjusted standardized residuals are reported beneath each percentage, with those greater than 2.0 and less than -2.0 indicating significant deviation from expected cell values (the total).

** Significant difference from expected value*

Table W. Metadata standards used by age group

| **Age** | **22-39** | **40-49** | **50+** | **Total** | ***X^2^; p*** |
| --- | --- | --- | --- | --- | --- |
| metadata standardized within my institution | 14.1%  (0.2) | 16.4%  (1.2) | 12.0%  (-1.2) | 13.8% | *X^2^* = 1.95  P= .377 |
| metadata standardized within my lab | 19.2%  (1.4) | 16.9%  (-0.1) | 14.8%  (-1.4) | 17.1% | *X^2^* = 2.38  P= .305 |
| None | 43.1%  (-2.5)* | 50.0%  (0.6) | 52.7%  (2.1)* | 48.2% | *X^2^* = 6.77  P= .034 |

Table shows percentages within each age group for 3 metadata standard options (“yes”) that may limit accessibility of data for outside researchers. Probability values correspond to results of Chi-square tests for each location. Adjusted standardized residuals are reported beneath each percentage, with those greater than 2.0 and less than -2.0 indicating significant deviation from expected cell values (the total). Chi-square values reported in results.

** Significant difference from expected value*

Table X. Perceptions of organizational support for data management and policies by age group

|  | **22-39** | | **40-49** | | **50+** | | **ANOVA** |
| --- | --- | --- | --- | --- | --- | --- | --- |
|  | ***M*** | **SD** | ***M*** | **SD** | ***M*** | **SD** | **F*; p*** |
| Managing data during the life of the project (short term) | 2.73 | 1.42 | 2.67 | 1.51 | 2.85 | 1.63 | F= .488  P= .614 |
| Managing and storing data beyond life of project (long-term) | 2.73 | 1.51 | 2.71 | 1.48 | 2.73 | 1.59 | F= .007  P= .993 |
| Provides training on best practices for data management | 2.62 | 1.39 | 2.42 | 1.42 | 2.35 | 1.39 | F= 1.63  P= .196 |
| Provides training or assistance on creating data management plans | 2.54 | 1.46 | 2.33 | 1.36 | 2.38 | 1.46 | F= .754  P= .471 |
| Provides assistance on creating metadata to describe my data or datasets | 2.49 | 1.41 | 2.25 | 1.47 | 2.30 | 1.39 | F= 1.11  P= .331 |
| Provides training on how to cite datasets | 2.58 | 1.42 | 2.35 | 1.34 | 2.24 | 1.35 | F= 2.66  P= .071 |
| Provides the necessary funds to support data management during the life of a research project (short-term) | 2.98 | 1.40 | 2.92 | 1.55 | 2.66 | 1.59 | F= 2.11  P= .123 |
| Provides the necessary funds to support data management beyond the life of a research project (long-term) | 2.50 | 1.38 | 2.40 | 1.51 | 2.34 | 1.48 | F= .551  P= .577 |
| Provides the necessary tools and technical support for data management during the life of a research project (short-term) | 3.08 | 1.40 | 3.04 | 1.43 | 2.97 | 1.56 | F= .267  P= .766 |
| Provides the necessary tools and technical support for data management beyond the life of a research project (long-term) | 2.62 | 1.40 | 2.70 | 1.39 | 2.53 | 1.50 | F= .435  P= .647 |

Table shows mean agreement (1= Disagree strongly, 2= disagree somewhat, 3= neither agree nor disagree, 4= agree somewhat, 5= agree strongly) and standard deviation for each item. MANOVA: F(20, 848) = 1.15, *p* = .291, Wilks’ Lambda= .948, partial eta squared= .026.

Univariate ANOVAs for each item within omnibus MANOVA.

Table Y. Value of data sharing and reuse by geographic location

|  | **North America** | | **Asia** | | **Europe** | | **Africa** | | | **South America** | | **Aus./NZ** | | **ANOVA** |
| --- | --- | --- | --- | --- | --- | --- | --- | --- | --- | --- | --- | --- | --- | --- |
|  | ***M*** | **SD** | ***M*** | **SD** | ***M*** | **SD** | ***M*** | **SD** | | ***M*** | **SD** | ***M*** | **SD** | **F*; p*** |
| Lack of access to data generated by other researchers is a major impediment to progress in science. | 3.91 | 1.07 | 4.39 | 0.70 | 3.96 | 1.01 | 4.07 | 1.00 | | 4.23 | 1.00 | 4.23 | 0.83 | F= 2.27  P= .046 |
| Lack of access to data generated by other researchers has restricted my ability to answer scientific questions. | 3.28_a_ | 1.26 | 4.07 | 0.96 | 3.27_a_ | 1.25 | 3.52 | | 1.40 | 3.49 | 1.34 | 3.62 | 1.04 | F= 3.39  P= .005 |

Table shows mean agreement (1= Disagree strongly, 2= disagree somewhat, 3= neither agree nor disagree, 4= agree somewhat, 5= agree strongly) and standard deviation for each item. MANOVA: F(10, 1214) = 1.95, *p* = .035, Wilks’ Lambda= .969, partial eta squared= .016.

Univariate ANOVAs for each item within omnibus MANOVA.

_a_ *= Differs significantly from Asia*

Table Z. Willingness to engage in data sharing and reuse by geographic region

|  | **North America** | | **Asia** | | **Europe** | | **Africa** | | | **South America** | | **Aus./NZ** | | **ANOVA** |
| --- | --- | --- | --- | --- | --- | --- | --- | --- | --- | --- | --- | --- | --- | --- |
|  | ***M*** | **SD** | ***M*** | **SD** | ***M*** | **SD** | ***M*** | **SD** | | ***M*** | **SD** | ***M*** | **SD** | **F; *p*** |
| I would use other researchers’ datasets if their datasets were easily accessible. | 4.28 | 0.82 | 4.37 | 0.69 | 4.35 | 0.85 | 4.34 | 0.85 | | 4.43 | 0.77 | 4.60 | 0.70 | F= .546  P= .742 |
| I would be willing to place at least some of my data into a central repository with no restrictions. | 4.29 | 0.98 | 4.23 | 0.77 | 4.05 | 1.25 | 4.26 | | 1.06 | 4.53 | 0.73 | 4.90 | 0.32 | F= 2.03  P= .073 |
| I would be willing to place all of my data into a central repository with no restrictions. | 3.24 | 1.44 | 3.17 | 1.20 | 3.07 | 1.40 | 3.08 | | 1.48 | 3.40 | 1.45 | 3.50 | 1.35 | F= .442  P= .819 |
| I would be more likely to make my data available if I could place conditions on access. | 3.42 | 1.28 | 3.83 | 0.89 | 3.68 | 1.06 | 3.97 | | 1.00 | 3.63 | 1.22 | 3.70 | 1.16 | F= 2.39  P= .037 |
| I am satisfied with my ability to integrate data from disparate sources to address research questions. | 3.10_a_ | 1.22 | 3.77 | 1.06 | 3.30 | 1.08 | 3.34 | | 1.30 | 3.07 | 1.31 | 3.40 | 1.35 | F= 2.37  P= .039 |
| I would be willing to share data across a broad group of researchers. | 4.41 | 0.81 | 4.23 | 0.60 | 4.38 | 0.83 | 4.37 | | 0.71 | 4.27 | 0.94 | 4.70 | 0.48 | F= .758  P= .581 |
| It is important that my data are cited when used by other researchers. | 4.50 | 0.78 | 4.37 | 0.84 | 4.63 | 0.68 | 4.42 | | 1.06 | 4.53 | 0.68 | 4.30 | 0.95 | F= .839  P= .522 |
| It is appropriate to create new datasets from shared data. | 4.21 | 0.90 | 4.34 | 0.73 | 4.05 | 1.12 | 4.24 | | 0.91 | 4.43 | 0.77 | 4.60 | 0.70 | F= 1.36  P= .238 |

Table shows mean agreement (1= Disagree strongly, 2= disagree somewhat, 3= neither agree nor disagree, 4= agree somewhat, 5= agree strongly) and standard deviation for each item. MANOVA: F(40, 2230.19) = 1.42, *p* = .042, Wilks’ Lambda= .896, partial eta squared= .022.

Univariate ANOVAs for each item within omnibus MANOVA.

_a_ *= Differs significantly from Asia*

Table AA. Perceived risks of data sharing and reuse by geographic region

|  | **North America** | | **Asia** | | **Europe** | | **Africa** | | **South America** | | **Aus./NZ** | | **ANOVA** |
| --- | --- | --- | --- | --- | --- | --- | --- | --- | --- | --- | --- | --- | --- |
|  | ***M*** | **SD** | ***M*** | **SD** | ***M*** | **SD** | ***M*** | **SD** | ***M*** | **SD** | ***M*** | **SD** | **F; *p*** |
| Data may be misinterpreted due to complexity of the data | 4.20 | 0.86 | 3.93 | 0.88 | 3.91 | 1.13 | 4.12 | 1.00 | 4.15 | 1.12 | 4.08 | 0.86 | F= 1.90  P= .093 |
| Data may be misinterpreted due to poor quality of the data | 4.23 | 0.84 | 4.07 | 0.93 | 4.10 | 0.96 | 4.26 | 0.96 | 3.97 | 1.21 | 4.23 | 0.73 | F= .892  P= .486 |
| Data may be used in ways other than intended | 4.31 | 0.81 | 3.93 | 0.99 | 3.98_a_ | 1.05 | 4.14 | 0.95 | 4.09 | 0.81 | 4.38 | 0.65 | F= 3.38  P= .005 |

Table shows mean agreement (1= Disagree strongly, 2= disagree somewhat, 3= neither agree nor disagree, 4= agree somewhat, 5= agree strongly) and standard deviation for each item. MANOVA: F(15, 1640.17) = 1.80, *p* = .030, Wilks’ Lambda= .956, partial eta squared= .015.

Univariate ANOVAs for each item within omnibus MANOVA.

_a_ *= Differs significantly from North America*

Table BB. Barriers to sharing data by geographic region

| **Continent** | **North America** | **Asia** | **Europe** | **Africa** | **South America** | **Aus./NZ** | **Total** | ***X^2^; p***** |
| --- | --- | --- | --- | --- | --- | --- | --- | --- |
| Lack of funding | 25.8%  (1.0) | 16.7%  (-1.5) | 24.3%  (-0.1) | 28.8%  (0.7) | 19.0%  (-0.9) | 23.1%  (-0.1) | 24.6% | *X^2^=* 3.61  *P=* .608 |
| Lack of standards | 15.2%  (-2.2) | 15.0%  (-0.6) | 21.4%  (1.0) | 23.1%  (1.0) | 31.0%  (2.3) | 23.1%  (0.5) | 17.8% | *X^2^=* 9.29  P= .096 |
| People don’t need them | 27.1%  (1.6) | 20.0%  (-0.9) | 24.3%  (-0.1) | 19.2%  (-1.0) | 14.3%  (-1.6) | 38.5%  (1.1) | 24.9% | *X^2^=* 6.53  P= .259 |
| There is insufficient time to make them available | 40.8%  (1.4) | 23.3%  (-2.6) | 41.7%  (0.7) | 30.8%  (-1.2) | 42.9%  (0.6) | 38.5%  (0.0) | 38.7% | *X^2^=* 8.84  P= .116 |
| There is no place to put them | 17.6%  (-0.3) | 16.7%  (-0.3) | 15.5%  (-0.7) | 25.0%  (1.4) | 14.3%  (-0.6) | 38.5%  (1.9) | 18.0% | *X^2^=* 6.35  P= .273 |
| They shouldn't be available | 13.5%  (0.5) | 13.3%  (0.1) | 15.5%  (0.8) | 7.7%  (-1.2) | 9.5%  (-0.7) | 7.7%  (-0.6) | 13.0% | *X^2^=* 2.76  P= .741 |
| Sponsor doesn't require it | 14.5%  (-0.5) | 16.7%  (0.4) | 15.5%  (0.1) | 15.4%  (0.1) | 19.0%  (0.7) | 7.7%  (-0.7) | 15.1% | *X^2^=* 1.32  P= .940 |
| Don't have the rights to make the data public | 25.6%  (-0.2) | 40.0%  (2.6) | 22.3%  (-0.9) | 25.0%  (-0.2) | 16.7%  (-1.4) | 30.8%  (0.4) | 25.9% | *X^2^=* 8.97  P= .114 |
| I would lose control of the data | 9.4%  (-0.7) | 11.7%  (0.4) | 10.7%  (0.2) | 11.5%  (0.4) | 11.9%  (0.4) | 7.7%  (-0.3) | 10.1% | *X^2^=* .764  P= .981 |
| I need to publish first | 40.6%  (-2.0) | 53.3%  (1.6) | 51.5%  (1.7) | 42.3%  (-0.2) | 40.5%  (-0.4) | 53.8%  (0.7) | 43.7% | *X^2^=* 7.18  P= .211 |
| I have insufficient skills to make my data available | 12.3%  (-0.9) | 11.7%  (-0.4) | 7.8%  (-1.8) | 23.1%  (2.2)* | 23.8%  (2.1)* | 23.1%  (1.0) | 13.3% | *X^2^=* 12.63  P= .028 |

Table shows percentages within each geographic location for each barrier (“yes”). Chi-square values for each barrier are reported. Adjusted standardized residuals are reported beneath each percentage, with those greater than 2.0 and less than -2.0 indicating significant deviation from expected cell values (the total).

** Significant difference from expected value*

*** Monte Carlo’s test of significance used due to possible small cell counts.*

Table CC. Conditions for use of your data by geographic region (percentage of those who selected “yes” from options of “yes,” “no,” and “not sure”)

| **Continent** |  | **North America** | **Asia** | **Europe** | **Africa** | **South America** | **Aus./NZ** | **Total** | ***X^2^; p*** |
| --- | --- | --- | --- | --- | --- | --- | --- | --- | --- |
| Co-authorship on publications resulting from use of the data. | **yes** | 29.2%  (-5.1)* | 63.3%  (4.1)* | 43.4%  (1.6) | 58.8%  (3.5)* | 43.6%  (1.0) | 7.1%  (-2.3)* | 36.2% | *X^2^*=52.08  P<.001 |
|  | **no** | 39.8%  (4.5)* | 16.3%  (-2.7) | 26.3%  (-1.7) | 21.6%  (-1.9) | 23.1%  (-1.4) | 28.6%  (-0.4) | 33.7% |  |
|  | **n/s** | 31.0%  (0.7) | 20.4%  (-1.5) | 30.3%  (0.0) | 19.6%  (-1.7) | 33.3%  (0.5) | 64.3%  (2.8) | 30.1% |  |
| Acknowledgement of the data providers in all disseminated work making use of the data. | **yes** | 87.8%  (-0.1) | 90.2%  (0.5) | 88.7%  (0.3) | 88.0%  (0.0) | 81.1%  (-1.3) | 92.9%  (0.6) | 87.8% | *X^2^*=4.54  P=.928 |
|  | **no** | 5.9%  (0.6) | 2.0%  (-1.2) | 5.2%  (-0.2) | 4.0%  (-0.5) | 8.1%  (0.7) | 7.1%  (0.3) | 5.5% |  |
|  | **n/s** | 6.3%  (-0.5) | 7.8%  (0.4) | 6.2%  (-0.2) | 8.0%  (0.4) | 10.8%  (1.0) | 0.0%  (-1.0) | 6.7% |  |
| Citation of the data providers in all disseminated work making use of the data. | **yes** | 85.3%  (0.2) | 86.0%  (0.2) | 78.0%  (-2.1) | 82.7%  (-0.5) | 97.4%  (2.2) | 100.0%  (1.6) | 85.1% | *X^2^*=12.04  P=.284 |
|  | **no** | 6.8%  (0.5) | 6.0%  (-0.1) | 9.0%  (1.1) | 5.8%  (-0.2) | 0.0%  (-1.7) | 0.0%  (-1.0) | 6.5% |  |
|  | **n/s** | 7.9%  (-0.7) | 8.0%  (-0.1) | 13.0%  (1.8) | 11.5%  (0.8) | 2.6%  (-1.3) | 0.0%  (-1.2) | 2.0% |  |
| The opportunity to collaborate on a project using the data. | **yes** | 51.0%  (-5.4)* | 75.0%  (2.5)* | 64.0%  (1.2) | 80.8%  (3.4)* | 81.6%  (2.9)* | 57.1%  (-0.1) | 58.7% | *X^2^*=40.68  P<.001 |
|  | **no** | 24.8%  (4.4)* | 7.7%  (-2.3)* | 14.0%  (-1.6) | 11.5%  (-1.5) | 2.6%  (-2.7)* | 21.4%  (0.2) | 19.7% |  |
|  | **n/s** | 24.1%  (2.2)* | 17.3%  (-0.8) | 22.0%  (0.1) | 7.7%  (-2.5)* | 15.8%  (-0.9) | 21.4%  (0.0) | 21.6% |  |
| Results based (at least in part) on the data could not be disseminated in any format without the data provider’s approval. | **yes** | 24.1%  (-3.7)* | 58.0%  (4.7)* | 26.5%  (-0.6) | 47.1%  (3.0)* | 36.8%  (1.1) | 7.1%  (-1.8) | 28.9% | *X^2^*=47.98  P<.001 |
|  | **no** | 55.0%  (2.9)* | 22.0%  (-4.2)* | 52.0%  (0.3) | 41.2%  (-1.4) | 52.6%  (-0.2) | 42.9%  (-0.6) | 50.8% |  |
|  | **n/s** | 20.9%  (0.6) | 20.0%  (0.0) | 21.4%  (0.3) | 11.8%  (-1.6) | 10.5%  (-1.5) | 50.0%  (2.8)* | 20.3% |  |
| At least part of the costs of data acquisition, retrieval, or provision must be recovered. | **yes** | 12.5%  (-1.7) | 25.0%  (2.2)* | 12.3%  (-0.6) | 21.2%  (1.5) | 21.6%  (1.3) | 0.0%  (-1.5) | 14.2% | *X^2^*=21.47  P=.022 |
|  | **no** | 66.1%  (2.5)* | 43.8%  (-2.8)* | 63.3%  (0.1) | 48.1%  (-2.3)* | 64.9%  (0.1) | 64.3%  (0.1) | 62.7% |  |
|  | **n/s** | 21.4%  (-1.4) | 31.3%  (1.4) | 24.5%  (0.4) | 30.8%  (1.4) | 13.5%  (-1.4) | 35.7%  (1.1) | 23.1% |  |
| Results based (at least in part) on the data could not be disseminated without the data provider having the opportunity to review the results and make suggestions or comments, but approval not required. | **yes** | 35.7%  (-0.8) | 46.9%  (1.5) | 28.9%  (-1.8) | 46.2%  (1.4) | 39.5%  (0.3) | 50.0%  (1.0) | 36.8% | *X^2^*=17.69  P=.063 |
|  | **no** | 46.4%  (2.8) | 28.6%  (-2.0) | 41.2%  (-0.3) | 30.8%  (-1.8) | 36.8%  (-0.7) | 35.7%  (-0.5) | 42.5% |  |
|  | **n/s** | 17.9%  (-2.4) | 24.5%  (-0.7) | 29.9%  (2.4) | 23.1%  (0.4) | 23.7%  (-0.5) | 14.3%  (-0.6) | 20.7% |  |
| Reprints of articles that make use of the data must be provided to the data provider. | **yes** | 41.1%  (-3.9)* | 71.4%  (3.6)* | 47.4%  (0.1) | 57.7%  (1.6) | 62.2%  (1.9) | 50.0%  (0.2) | 46.7% | *X^2^*=33.27  P=.001 |
|  | **no** | 45.0%  (4.6)* | 16.3%  (-3.3)* | 30.9%  (-1.7) | 30.8%  (-1.2) | 21.6%  (-2.2)* | 42.9%  (0.3) | 38.6% |  |
|  | **n/s** | 13.9%  (-0.8) | 12.2%  (-0.5) | 21.6%  (2.1)* | 11.5%  (-0.7) | 16.2%  (0.3) | 7.1%  (-0.8) | 14.7% |  |
| The data provider is given a complete list of all products that make use of the data, including articles, presentations, educational materials, etc. | **yes** | 39.2%  (-3.1)* | 62.0%  (2.7)* | 39.4%  (-0.9) | 61.5%  (2.7)* | 57.9%  (1.8) | 42.9%  (-0.1) | 43.7% | *X^2^*=24.49  P=.007 |
|  | **no** | 41.7%  (3.0)* | 18.0%  (-3.0)* | 40.4%  (0.6) | 25.0%  (-2.0)* | 23.7%  (-1.8) | 42.9%  (0.4) | 37.6% |  |
|  | **n/s** | 19.0%  (0.3) | 20.0%  (0.2) | 20.2%  (0.4) | 13.5%  (-1.0) | 18.4%  (-0.1) | 14.3%  (-0.4) | 18.7% |  |
| Legal permission for data use is obtained. | **yes** | 28.9%  (-3.2)* | 55.1%  (3.4)* | 32.7%  (-0.1) | 52.9%  (3.1)* | 36.8%  (0.5) | 14.3%  (-1.5) | 33.2% | *X^2^*=34.50  P<.001 |
|  | **no** | 50.6%  (3.5)* | 24.5%  (-3.1)* | 37.8%  (-1.7) | 31.4%  (-2.1)* | 44.7%  (-0.1) | 71.4%  (2.0)* | 45.6% |  |
|  | **n/s** | 20.5%  (-0.6) | 20.4%  (-0.1) | 29.6%  (2.2)* | 15.7%  (-1.0) | 18.4%  (-0.4) | 14.3%  (-0.6) | 21.2% |  |
| Mutual agreement on reciprocal sharing of data. | **yes** | 37.6%  (-6.1)* | 67.3%  (3.1)* | 56.7%  (2.2)* | 69.2%  (3.4)* | 65.8%  (2.5)* | 35.7%  (-0.8) | 46.3% | *X^2^*=51.00  P<.001 |
|  | **no** | 41.7%  (5.6)* | 10.2%  (-3.7)* | 27.8%  (-1.4) | 15.4%  (-3.0)* | 18.4%  (-2.1)* | 35.7%  (0.1) | 34.1% |  |
|  | **n/s** | 20.7%  (1.0) | 22.4%  (0.5) | 15.5%  (-1.1) | 15.4%  (-0.8) | 15.8%  (-0.6) | 28.6%  (0.9) | 19.6% |  |
| The data provider is given and agrees to a statement of uses to which the data will be put. | **yes** | 38.5%  (-4.0)* | 66.7%  (3.2)* | 51.5%  (1.6) | 53.8%  (1.4) | 56.8%  (1.6) | 28.6%  (-1.2) | 44.3% | *X^2^*=41.64  P<.001 |
|  | **no** | 40.4%  (5.8)* | 8.3%  (-3.7)* | 22.7%  (-2.3)* | 19.2%  (-2.1)* | 18.9%  (-1.8) | 28.6%  (-0.3) | 32.7% |  |
|  | **n/s** | 21.1%  (-1.7) | 25.0%  (0.3) | 25.8%  (0.7) | 26.9%  (0.7) | 24.3%  (0.2) | 42.9%  (1.8) | 23.1% |  |

Table shows percentages within each geographic location for proposed condition (“yes,” “no,” and “not sure”). Chi-square tests for each condition are reported. Adjusted standardized residuals are reported beneath each percentage, with those greater than 2.0 and less than -2.0 indicating significant deviation from expected cell values (the total). Chi-square values reported in results.

** Significant difference from expected value*

*** Monte Carlo’s test of significance used due to possible small cell counts.*

Table DD. Amount of data made available to others by geographic region (1= None, 2= Some, 3= Most, 4= All)

|  | **North America** | | **Asia** | | **Europe** | | **Africa** | | **South America** | | **Aus./NZ** | |
| --- | --- | --- | --- | --- | --- | --- | --- | --- | --- | --- | --- | --- |
|  | ***M*** | **SD** | ***M*** | **SD** | ***M*** | **SD** | ***M*** | **SD** | ***M*** | **SD** | ***M*** | **SD** |
| How much of your data do you make available to others? | 2.57 | 0.90 | 2.30 | 0.77 | 2.52 | 0.84 | 2.57 | 0.72 | 2.53 | 0.82 | 2.59 | 0.94 |

Tables shows the means and standard deviations of each geographic region as they determine the results of a one-way analysis of variance (ANOVA) with amount of data made available to others as the dependent variable. F(5, 823) = 1.15, *p* = .331.

Table EE. Data accessibility by geographic region

|  | **North America** | | **Asia** | | **Europe** | | **Africa** | | | **South America** | | **Aus./NZ** | | **ANOVA** |
| --- | --- | --- | --- | --- | --- | --- | --- | --- | --- | --- | --- | --- | --- | --- |
|  | ***M*** | **SD** | ***M*** | **SD** | ***M*** | **SD** | ***M*** | **SD** | | ***M*** | **SD** | ***M*** | **SD** | **F; *p*** |
| I share my data with others. | 4.08 | 1.08 | 3.93 | 1.00 | 3.88 | 1.17 | 3.83 | 0.96 | | 4.30 | 0.78 | 4.20 | 1.08 | F= 1.52  P= .180 |
| Others need permission to access my data. | 3.44_a_ | 1.37 | 4.36_b_ | 0.93 | 3.79 | 1.24 | 4.13_b_ | | 1.04 | 3.57_a_ | 1.17 | 3.13_a_ | 1.30 | F= 7.68  P<.001 |
| Others can access my data easily. | 3.11 | 1.36 | 2.91 | 1.34 | 3.12 | 1.34 | 2.72 | | 1.25 | 3.24 | 1.40 | 2.93 | 1.39 | F= 1.01  P= .409 |

Table shows mean agreement (1= Disagree strongly, 2= disagree somewhat, 3= neither agree nor disagree, 4= agree somewhat, 5= agree strongly) and standard deviation for each item. MANOVA: F(15, 1921.75) = 3.13, *p* < .001, Wilks’ Lambda= .936, partial eta squared= .022.

Univariate ANOVAs for each item within omnibus MANOVA.

*_a_ = Tukey’s post-hoc analysis shows significant pairwise difference from Asia*

*_b_ = Tukey’s post-hoc analysis shows significant pairwise difference from North America*

Table FF. Data storage by geographic region (excludes Other, Dropbox/Google/Figshare/Cloud, External Hard Disk/Drive Storage, and Other Server because those answers were derived from text included with “other”).

| **Continent** | **North America** | **Asia** | **Europe** | **Africa** | **South America** | **Aus./NZ** | **Total** | ***X^2^; p***** |
| --- | --- | --- | --- | --- | --- | --- | --- | --- |
| On my institution’s server | 67.7%  (-0.3) | 66.1%  (-0.3) | 75.7%  (1.9) | 59.6%  (-1.3) | 60.5%  (-1.1) | 78.6%  (0.9) | 68.1% | *X^2^* = 6.50  P= .263 |
| On the principal investigator’s server | 54.2%  (0.8) | 48.8%  (-0.6) | 44.2%  (-1.8) | 55.0%  (0.3) | 61.5%  (1.1) | 60.0%  (0.4) | 53.0% | *X^2^* =4.60  P=.467 |
| On a departmental server | 48.2%  (-0.1) | 47.7%  (-0.1) | 51.8%  (0.7) | 46.5%  (-0.3) | 47.1%  (-0.2) | 44.4%  (-0.2) | 48.4% | *X^2^* =.541  P=.989 |
| On my personal computer | 92.4%  (-0.7) | 96.8%  (1.3) | 88.7%  (-1.9) | 98.2%  (1.6) | 95.3%  (0.6) | 100.0%  (1.1) | 93.0% | *X^2^* =8.64  P=.121 |
| On paper in my office | 62.4%  (-2.1)* | 76.0%  (1.6) | 61.7%  (-0.8) | 83.0%  (2.6)* | 73.0%  (1.0) | 58.3%  (-0.5) | 65.4% | *X^2^* =12.28  P=.030 |
| In a discipline-based repository (e.g., NEON or LTER) | 29.2%  (1.4) | 19.4%  (-1.1) | 28.9%  (0.3) | 10.3%  (-2.5) | 29.4%  (0.3) | 33.3%  (0.5) | 27.4% | *X^2^* =7.93  P=.159 |
| In a publisher or publisher-related repository (e.g., specific publisher or Dryad) | 17.7%  (-1.4) | 35.9%  (2.7) | 18.2%  (-0.3) | 18.4%  (-0.1) | 21.2%  (0.3) | 22.2%  (0.2) | 19.3% | *X^2^* =7.73  P=.165 |
| Other data repository or archive (e.g., national data center) | 29.5%  (-1.8) | 35.9%  (0.5) | 37.6%  (1.2) | 33.3%  (0.2) | 36.7%  (0.6) | 45.5%  (1.0) | 32.0% | *X^2^* =3.86  P=.572 |
| In my institution’s repository | 28.3%  (-3.0)* | 53.5%  (2.8)* | 31.1%  (-0.3) | 44.4%  (1.7) | 37.5%  (0.6) | 60.0%  (1.9) | 32.8% | *X^2^* =16.93  P=.005 |

Table shows percentage within each geographic location for each storage location (“yes” within dichotomized variable). Chi-square tests for each condition are reported. Adjusted standardized residuals are reported beneath each percentage, with those greater than 2.0 and less than -2.0 indicating significant deviation from expected cell values (the total). Chi-square values reported in results.

** Significant difference from expected value*

*** Monte Carlo’s test of significance used due to possible small cell counts.*

Table GG. Metadata standards used by geographic region

| **Continent** | **North America** | **Asia** | **Europe** | **Africa** | **South America** | **Aus./NZ** | **Total** | ***X^2^; p**** |
| --- | --- | --- | --- | --- | --- | --- | --- | --- |
| metadata standardized within my institution | 12.1%  (-2.2) | 19.5%  (1.5) | 13.3%  (-0.3) | 23.5%  (2.3) | 15.4%  (0.3) | 15.8%  (0.2) | 14.1% | *X^2^* =9.16  P=.104 |
| metadata standardized within my lab | 16.8%  (0.2) | 23.0%  (1.7) | 13.3%  (-1.1) | 13.2%  (-0.8) | 19.2%  (0.5) | 10.5%  (-0.7) | 16.6% | *X^2^* =4.94  P=.430 |
| None | 48.3%  (0.2) | 40.2%  (-1.5) | 50.7%  (0.7) | 50.0%  (0.3) | 46.2%  (-0.3) | 52.6%  (0.4) | 48.0% | *X^2^* =2.88  P=.722 |

Table shows percentages within each geographic region for 3 metadata standard options (“yes”) that may limit accessibility of data for outside researchers. -square tests for each location are reported. Adjusted standardized residuals are reported beneath each percentage, with those greater than 2.0 and less than -2.0 indicating significant deviation from expected cell values (the total).  ** Monte Carlo’s test of significance used due to possible small cell counts.*

Table HH. Perceptions of organizational support by geographic region

|  | **North America** | | **Asia** | | **Europe** | | **Africa** | | | **South America** | | **Aus./NZ** | | **ANOVA** |
| --- | --- | --- | --- | --- | --- | --- | --- | --- | --- | --- | --- | --- | --- | --- |
|  | ***M*** | **SD** | ***M*** | **SD** | ***M*** | **SD** | ***M*** | **SD** | | ***M*** | **SD** | ***M*** | **SD** | **F; *p*** |
| Managing data during the life of the project (short term) | 2.60 | 1.54 | 3.08 | 1.55 | 2.91 | 1.57 | 2.89 | 1.35 | | 2.55 | 1.45 | 3.09 | 1.51 | F= .834  P= .526 |
| Managing and storing data beyond life of project (long-term) | 2.74 | 1.52 | 2.78 | 1.57 | 2.80 | 1.65 | 2.74 | | 1.42 | 2.17 | 1.44 | 3.09 | 1.51 | F= .931  P= .461 |
| Provides training on best practices for data management | 2.49 | 1.37 | 2.78 | 1.62 | 2.24 | 1.29 | 2.49 | | 1.42 | 2.31 | 1.47 | 2.91 | 1.58 | F= 1.03  P= .398 |
| Provides training or assistance on creating data management plans | 2.52 | 1.46 | 2.89 | 1.58 | 2.00_a_ | 1.16 | 2.46 | | 1.36 | 2.14 | 1.36 | 2.73 | 1.56 | F= 2.53  P= .029 |
| Provides assistance on creating metadata to describe my data or datasets | 2.46 | 1.43 | 2.95 | 1.53 | 1.97_a_ | 1.22 | 2.09 | | 1.36 | 2.17 | 1.42 | 2.64 | 1.43 | F= 3.03  P= .011 |
| Provides training on how to cite datasets | 2.39 | 1.33 | 3.05 | 1.68 | 2.24 | 1.27 | 2.54 | | 1.54 | 2.31 | 1.44 | 2.27 | 1.56 | F= 1.92  P= .090 |
| Provides the necessary funds to support data management during the life of a research project (short-term) | 2.77 | 1.54 | 3.22 | 1.44 | 2.82 | 1.53 | 2.91 | | 1.36 | 2.90 | 1.50 | 3.36 | 1.63 | F= .875  P= .498 |
| Provides the necessary funds to support data management beyond the life of a research project (long-term) | 2.34 | 1.42 | 2.62 | 1.44 | 2.42 | 1.52 | 2.60 | | 1.48 | 2.28 | 1.49 | 3.27 | 1.49 | F= 1.23  P= .296 |
| Provides the necessary tools and technical support for data management during the life of a research project (short-term) | 3.03 | 1.45 | 3.22 | 1.44 | 3.03 | 1.52 | 2.91 | | 1.52 | 2.76 | 1.41 | 3.36 | 1.63 | F= .474  P= .796 |
| Provides the necessary tools and technical support for data management beyond the life of a research project (long-term) | 2.62 | 1.43 | 2.73 | 1.35 | 2.56 | 1.53 | 2.37 | | 1.40 | 2.52 | 1.43 | 3.09 | 1.51 | F= .533  P =.752 |

Table shows mean agreement (1= Disagree strongly, 2= disagree somewhat, 3= neither agree nor disagree, 4= agree somewhat, 5= agree strongly) and standard deviation for each item. MANOVA: F(50, 1973.59) = 1.43, *p* = .028, Wilks’ Lambda= .851, partial eta squared= .032.

Univariate ANOVAs for each item within omnibus MANOVA.

*_a_ = Tukey’s post-hoc analysis shows significant pairwise difference from Asia*

| Table II. Value of data sharing and reuse by subject discipline | | | | |
| --- | --- | --- | --- | --- |
|  | **Subject Discipline** | **Mean** | **SD** | **F; *p*** |
| Lack of access to data generated by other researchers or institutions is a major impediment to progress in science. | Agriculture and Natural Resources | 3.91 | 1.10 | F= 2.14  P= .004 |
|  | Atmospheric science | 3.97 | 0.95 |  |
|  | Biology | 3.89 | 0.99 |  |
|  | Business | 3.00 | 1.33 |  |
|  | Computer science | 4.32 | 1.16 |  |
|  | Ecology | 4.01 | 1.00 |  |
|  | Education | 3.50 | 1.00 |  |
|  | Engineering | 4.19 | 1.04 |  |
|  | Environmental science | 4.26 | 0.79 |  |
|  | Geology | 3.86 | 1.17 |  |
|  | Hydrology | 4.05 | 1.05 |  |
|  | Information science | 4.17 | 0.97 |  |
|  | Law | 4.00 | 0.00 |  |
|  | Medicine/Health Sciences | 3.36 | 1.33 |  |
|  | Physical sciences | 3.90 | 1.14 |  |
|  | Psychology | 3.62 | 1.33 |  |
|  | Social sciences | 4.27 | 0.69 |  |
|  | Other (please specify) | 3.75 | 1.14 |  |
|  | Humanities | 4.25 | 0.96 |  |
|  |  |  |  |  |
| Lack of access to data generated by other researchers or institutions has restricted my ability to answer scientific questions. | Agriculture and Natural Resources | 3.50 | 1.19 | F= 2.09  P= .005 |
|  | Atmospheric science | 3.67 | 1.11 |  |
|  | Biology | 3.27 | 1.19 |  |
|  | Business | 2.30_a_ | 1.25 |  |
|  | Computer science | 4.00 | 1.33 |  |
|  | Ecology | 3.25 | 1.25 |  |
|  | Education | 3.17 | 1.19 |  |
|  | Engineering | 3.59 | 1.25 |  |
|  | Environmental science | 3.69 | 1.02 |  |
|  | Geology | 3.36 | 1.22 |  |
|  | Hydrology | 3.41 | 1.53 |  |
|  | Information science | 3.46 | 1.33 |  |
|  | Law | 2.67 | 1.53 |  |
|  | Medicine/Health Sciences | 3.00_a_ | 1.41 |  |
|  | Physical sciences | 3.23 | 1.45 |  |
|  | Psychology | 2.77 | 1.36 |  |
|  | Social sciences | 3.47 | 1.25 |  |
|  | Other (please specify) | 2.81 | 1.28 |  |
|  | Humanities | 4.00 | 0.82 |  |
|  |  |  |  |  |

Table shows mean agreement (1= Disagree strongly, 2= disagree somewhat, 3= neither agree nor disagree, 4= agree somewhat, 5= agree strongly) and standard deviation for each item. MANOVA: F(36, 1192) = 1.76, *p* = .004, Wilks’ Lambda= .902, partial eta squared= .051

Univariate ANOVAs for each item within omnibus MANOVA also reported.

*_a_ = Tukey’s post-hoc analysis shows significant pairwise difference from Environmental Science*

| Table JJ. Willingness to engage in data sharing and reuse by subject discipline | | | | |
| --- | --- | --- | --- | --- |
|  | **Subject Discipline** | **Mean** | **Std. Deviation** | **F; *p*** |
| I would use other researchers' datasets if their datasets were easily accessible. | Agriculture and Natural Resources | 4.21 | 0.83 | F= 1.78  P= .025 |
|  | Atmospheric science | 4.33 | 0.73 |  |
|  | Biology | 4.36 | 0.74 |  |
|  | Business | 3.86 | 0.38 |  |
|  | Computer science | 4.56 | 0.63 |  |
|  | Ecology | 4.32 | 0.78 |  |
|  | Education | 4.09 | 0.70 |  |
|  | Engineering | 4.50 | 0.79 |  |
|  | Environmental science | 4.51 | 0.75 |  |
|  | Geology | 4.45 | 0.69 |  |
|  | Hydrology | 4.71 | 0.59 |  |
|  | Information science | 4.44 | 0.74 |  |
|  | Law | 4.00 | 1.00 |  |
|  | Medicine/Health Sciences | 3.81 | 0.83 |  |
|  | Physical sciences | 4.12 | 0.99 |  |
|  | Psychology | 4.17 | 0.72 |  |
|  | Social sciences | 4.22 | 0.89 |  |
|  | Other (please specify) | 3.96 | 1.17 |  |
|  | Humanities | 4.67 | 0.82 |  |
|  |  |  |  |  |
| I would be willing to place at least some of my data into a central data repository with no restrictions. | Agriculture and Natural Resources | 4.15_a_ | 0.96 | F= 3.95  P<.001 |
|  | Atmospheric science | 4.52_a_ | 0.75 |  |
|  | Biology | 4.49_a_ | 0.79 |  |
|  | Business | 3.57 | 1.27 |  |
|  | Computer science | 4.63_a_ | 0.62 |  |
|  | Ecology | 4.36_a_ | 0.90 |  |
|  | Education | 4.27_a_ | 0.65 |  |
|  | Engineering | 4.56_a_ | 0.62 |  |
|  | Environmental science | 4.42_a_ | 0.86 |  |
|  | Geology | 4.55_a_ | 0.52 |  |
|  | Hydrology | 4.59_a_ | 0.62 |  |
|  | Information science | 4.61_a_ | 0.69 |  |
|  | Law | 3.33 | 2.08 |  |
|  | Medicine/Health Sciences | 2.94 | 1.57 |  |
|  | Physical sciences | 4.00 | 1.38 |  |
|  | Psychology | 3.58 | 1.38 |  |
|  | Social sciences | 3.89 | 1.34 |  |
|  | Other (please specify) | 4.18_a_ | 1.06 |  |
|  | Humanities | 3.67 | 1.21 |  |
|  |  |  |  |  |
| I would be willing to place all of my data into a central data repository with no restrictions. | Agriculture and Natural Resources | 2.79 | 1.35 | F= 2.99  P<.001 |
|  | Atmospheric science | 3.37 | 1.47 |  |
|  | Biology | 3.54_a_ | 1.47 |  |
|  | Business | 2.43 | 1.40 |  |
|  | Computer science | 3.81_a_ | 1.42 |  |
|  | Ecology | 3.32_a_ | 1.30 |  |
|  | Education | 3.27 | 1.01 |  |
|  | Engineering | 3.61_a_ | 1.34 |  |
|  | Environmental science | 3.46_a_ | 1.38 |  |
|  | Geology | 3.36 | 1.50 |  |
|  | Hydrology | 3.65_a_ | 1.17 |  |
|  | Information science | 3.75_a_ | 1.38 |  |
|  | Law | 2.00 | 1.00 |  |
|  | Medicine/Health Sciences | 1.88 | 1.15 |  |
|  | Physical sciences | 2.63 | 1.50 |  |
|  | Psychology | 2.75 | 1.66 |  |
|  | Social sciences | 2.63 | 1.47 |  |
|  | Other (please specify) | 3.14 | 1.35 |  |
|  | Humanities | 2.67 | 1.37 |  |
|  |  |  |  |  |
| I would be more likely to make my data available if I could place conditions on access. | Agriculture and Natural Resources | 3.85 | 0.93 | F= 1.72  P= .033 |
|  | Atmospheric science | 3.37 | 1.25 |  |
|  | Biology | 3.62 | 1.27 |  |
|  | Business | 3.57 | 0.54 |  |
|  | Computer science | 4.00 | 1.21 |  |
|  | Ecology | 3.46 | 1.25 |  |
|  | Education | 3.82 | 0.87 |  |
|  | Engineering | 2.78 | 1.11 |  |
|  | Environmental science | 3.54 | 1.18 |  |
|  | Geology | 3.82 | 0.98 |  |
|  | Hydrology | 3.65 | 1.62 |  |
|  | Information science | 3.25 | 1.16 |  |
|  | Law | 3.33 | 2.08 |  |
|  | Medicine/Health Sciences | 4.00 | 0.82 |  |
|  | Physical sciences | 3.08 | 1.53 |  |
|  | Psychology | 4.17 | 0.94 |  |
|  | Social sciences | 3.22 | 1.25 |  |
|  | Other (please specify) | 3.75 | 1.27 |  |
|  | Humanities | 4.17 | 0.98 |  |
|  |  |  |  |  |
| I am satisfied with my ability to integrate data from disparate sources to address research questions. | Agriculture and Natural Resources | 3.32 | 1.16 | F= 1.79  P= .023 |
|  | Atmospheric science | 3.70_b_ | 1.17 |  |
|  | Biology | 3.13 | 1.17 |  |
|  | Business | 3.57 | 0.98 |  |
|  | Computer science | 2.88 | 1.50 |  |
|  | Ecology | 3.19 | 1.10 |  |
|  | Education | 3.36 | 1.29 |  |
|  | Engineering | 2.94 | 1.35 |  |
|  | Environmental science | 3.36 | 1.15 |  |
|  | Geology | 3.09 | 1.45 |  |
|  | Hydrology | 3.82_b_ | 1.24 |  |
|  | Information science | 2.89 | 1.45 |  |
|  | Law | 2.33 | 0.58 |  |
|  | Medicine/Health Sciences | 3.06 | 1.18 |  |
|  | Physical sciences | 2.96 | 1.55 |  |
|  | Psychology | 3.33 | 0.78 |  |
|  | Social sciences | 2.44 | 1.01 |  |
|  | Other (please specify) | 3.46 | 0.96 |  |
|  | Humanities | 2.83 | 1.47 |  |
|  |  |  |  |  |
| I would be willing to share data across a broad group of researchers. | Agriculture and Natural Resources | 4.15 | 0.96 | F= 3.79  P< .001 |
|  | Atmospheric science | 4.48_a_ | 0.64 |  |
|  | Biology | 4.44_a_ | 0.64 |  |
|  | Business | 3.43 | 1.51 |  |
|  | Computer science | 4.56 | 0.63 |  |
|  | Ecology | 4.54_a,c_ | 0.64 |  |
|  | Education | 3.91 | 0.70 |  |
|  | Engineering | 4.44 | 0.78 |  |
|  | Environmental science | 4.56_a,c_ | 0.70 |  |
|  | Geology | 4.36 | 0.51 |  |
|  | Hydrology | 4.71_a,c_ | 0.59 |  |
|  | Information science | 4.58_a,c,d_ | 0.55 |  |
|  | Law | 3.00 | 1.73 |  |
|  | Medicine/Health Sciences | 3.63 | 0.96 |  |
|  | Physical sciences | 4.50_a_ | 0.93 |  |
|  | Psychology | 4.25 | 0.87 |  |
|  | Social sciences | 4.07 | 1.04 |  |
|  | Other (please specify) | 4.36 | 0.73 |  |
|  | Humanities | 4.00 | 0.89 |  |
|  |  |  |  |  |
| It is important that my data are cited when used by other researchers. | Agriculture and Natural Resources | 4.51 | 0.66 | F= 2.57  P<.001 |
|  | Atmospheric science | 4.59 | 0.64 |  |
|  | Biology | 4.64_d_ | 0.54 |  |
|  | Business | 4.14 | 0.90 |  |
|  | Computer science | 4.63 | 0.81 |  |
|  | Ecology | 4.62_d,e_ | 0.61 |  |
|  | Education | 3.73 | 1.27 |  |
|  | Engineering | 3.94 | 1.11 |  |
|  | Environmental science | 4.54 | 0.80 |  |
|  | Geology | 4.45 | 1.04 |  |
|  | Hydrology | 4.76_d_ | 0.44 |  |
|  | Information science | 4.39 | 0.99 |  |
|  | Law | 3.00 | 1.00 |  |
|  | Medicine/Health Sciences | 4.56 | 0.73 |  |
|  | Physical sciences | 4.46 | 0.78 |  |
|  | Psychology | 4.67 | 0.65 |  |
|  | Social sciences | 4.26 | 1.02 |  |
|  | Other (please specify) | 4.68 | 0.61 |  |
|  | Humanities | 4.50 | 0.84 |  |
|  |  |  |  |  |
| It is appropriate to create new datasets from shared data. | Agriculture and Natural Resources | 4.02 | 0.92 | F= 1.64  P= .047 |
|  | Atmospheric science | 4.15 | 0.95 |  |
|  | Biology | 4.46 | 0.72 |  |
|  | Business | 3.86 | 0.69 |  |
|  | Computer science | 4.63 | 0.89 |  |
|  | Ecology | 4.23 | 0.88 |  |
|  | Education | 4.00 | 0.63 |  |
|  | Engineering | 4.17 | 0.99 |  |
|  | Environmental science | 4.32 | 1.00 |  |
|  | Geology | 4.73 | 0.47 |  |
|  | Hydrology | 4.53 | 0.87 |  |
|  | Information science | 4.22 | 0.83 |  |
|  | Law | 4.00 | 1.00 |  |
|  | Medicine/Health Sciences | 4.19 | 0.83 |  |
|  | Physical sciences | 4.21 | 1.10 |  |
|  | Psychology | 3.50 | 1.17 |  |
|  | Social sciences | 4.22 | 1.01 |  |
|  | Other (please specify) | 3.86 | 1.01 |  |
|  | Humanities | 3.83 | 0.75 |  |
|  |  |  |  |  |

Table shows mean agreement (1= Disagree strongly, 2= disagree somewhat, 3= neither agree nor disagree, 4= agree somewhat, 5= agree strongly) and standard deviation for each item. MANOVA: [F(144, 3692.28) = 1.84, *p* < .001, Wilks’ Lambda= .601, partial eta squared= .062.

Univariate ANOVAs for each item within omnibus MANOVA also reported.

*_a_ = Tukey’s post-hoc analysis shows significant pairwise difference from Medicine/Health Sciences*

*_b_ = Tukey’s post-hoc analysis shows significant pairwise difference from Social Sciences*

*_c_ = Tukey’s post-hoc analysis shows significant pairwise difference from Business*

*_d_ = Tukey’s post-hoc analysis shows significant pairwise difference from Law*

*_e_ = Tukey’s post-hoc analysis shows significant pairwise difference from Education*

| Table KK. Perceived risks of data sharing by subject discipline | | | | |
| --- | --- | --- | --- | --- |
|  | **Subject Discipline** | **Mean** | **Std. Deviation** | **F; *p*** |
| Data may be misinterpreted due to complexity of the data. | Agriculture and Natural Resources | 4.33 | 0.93 | F= 1.02  P= .434 |
|  | Atmospheric science | 4.03 | 0.90 |  |
|  | Biology | 3.80 | 1.10 |  |
|  | Business | 3.82 | 0.98 |  |
|  | Computer science | 4.47 | 0.61 |  |
|  | Ecology | 4.05 | 0.96 |  |
|  | Education | 4.23 | 0.83 |  |
|  | Engineering | 4.28 | 0.89 |  |
|  | Environmental science | 4.09 | 0.97 |  |
|  | Geology | 3.71 | 0.61 |  |
|  | Hydrology | 4.17 | 0.65 |  |
|  | Information science | 4.17 | 0.96 |  |
|  | Law | 4.67 | 0.58 |  |
|  | Medicine/Health Sciences | 4.19 | 0.75 |  |
|  | Physical sciences | 4.24 | 1.06 |  |
|  | Psychology | 4.17 | 0.94 |  |
|  | Social sciences | 4.19 | 1.06 |  |
|  | Other (please specify) | 4.13 | 0.90 |  |
|  | Humanities | 4.40 | 0.89 |  |
|  |  |  |  |  |
| Data may be misinterpreted due to poor quality of the data. | Agriculture and Natural Resources | 4.33 | 0.82 | F= 1.98  P= .009 |
|  | Atmospheric science | 4.09 | 0.93 |  |
|  | Biology | 4.20 | 0.78 |  |
|  | Business | 3.55 | 1.13 |  |
|  | Computer science | 4.42 | 0.69 |  |
|  | Ecology | 4.11 | 0.82 |  |
|  | Education | 4.23 | 0.73 |  |
|  | Engineering | 4.44 | 0.92 |  |
|  | Environmental science | 4.23 | 0.88 |  |
|  | Geology | 3.43 | 1.02 |  |
|  | Hydrology | 4.43 | 0.79 |  |
|  | Information science | 4.35 | 0.83 |  |
|  | Law | 4.67 | 0.58 |  |
|  | Medicine/Health Sciences | 4.05 | 0.92 |  |
|  | Physical sciences | 4.38 | 0.90 |  |
|  | Psychology | 3.92 | 1.16 |  |
|  | Social sciences | 4.25 | 1.05 |  |
|  | Other (please specify) | 3.80 | 1.10 |  |
|  | Humanities | 4.40 | 0.89 |  |
|  |  |  |  |  |
| Data may be used in other ways than intended. | Agriculture and Natural Resources | 4.24 | 0.88 | F= .924  P= .527 |
|  | Atmospheric science | 4.13 | 0.91 |  |
|  | Biology | 4.02 | 0.88 |  |
|  | Business | 3.64 | 1.03 |  |
|  | Computer science | 4.26 | 0.81 |  |
|  | Ecology | 4.26 | 0.74 |  |
|  | Education | 4.38 | 0.65 |  |
|  | Engineering | 4.32 | 0.69 |  |
|  | Environmental science | 4.22 | 0.90 |  |
|  | Geology | 3.79 | 0.89 |  |
|  | Hydrology | 4.04 | 1.07 |  |
|  | Information science | 4.38 | 0.84 |  |
|  | Law | 4.33 | 0.58 |  |
|  | Medicine/Health Sciences | 4.19 | 0.87 |  |
|  | Physical sciences | 4.45 | 0.91 |  |
|  | Psychology | 4.00 | 1.04 |  |
|  | Social sciences | 4.22 | 1.10 |  |
|  | Other (please specify) | 4.17 | 1.02 |  |
|  | Humanities | 4.40 | 0.89 |  |
|  |  |  |  |  |

Table shows mean agreement (1= Disagree strongly, 2= disagree somewhat, 3= neither agree nor disagree, 4= agree somewhat, 5= agree strongly) and standard deviation for each item.

Probability value corresponds to univariate ANOVAs for each item within omnibus MANOVA (non-significant). All other statistics are reported in the results.

Table LL. Barriers to data sharing by subject discipline

|  | Lack of funding | Lack of standards | People don’t need them | Insufficient time | No place to put them | Should not be available | Sponsor does not require | Don’t have rights | I’d lose control of data | Need to publish first | Insufficient skills |
| --- | --- | --- | --- | --- | --- | --- | --- | --- | --- | --- | --- |
| Agriculture and Natural Resources | 26.7%  (0.4) | 13.3%  (-1.0) | 25.0%  (0.0) | 36.7%  (-0.3) | 6.7%  (-2.4) | 10.0%  (-0.7) | 13.3%  (-0.4) | 25.0%  (-0.2) | 13.3%  (0.9) | 45.0%  (0.2) | 10.0%  (-0.8) |
| Atmospheric science | 28.2%  (0.5) | 12.8%  (-0.9) | 25.6%  (0.1) | 35.9%  (-0.4) | 17.9%  (0.0) | 12.8%  (-0.1) | 7.7%  (-1.3) | 25.6%  (-0.1) | 5.1%  (-1.1) | 38.5%  (-0.7) | 7.7%  (-1.1) |
| Biology | 21.7%  (-0.5) | 23.9%  (1.1) | 30.4%  (0.9) | 37.0%  (-0.2) | 30.4%  (2.3) | 8.7%  (-0.9) | 21.7%  (1.3) | 15.2%  (-1.7) | 13.0%  (0.7) | 65.2%  3(.1)* | 19.6%  (1.3) |
| Business | 16.7%  (-0.6) | 0.0%  (-1.6) | 25.0%  (0.0) | 0.0%  (-2.8)* | 0.0%  (-1.6) | 8.3%  (-0.5) | 8.3%  (-0.7) | 16.7%  (-0.8) | 8.3%  (-0.2) | 66.7%  (1.6) | 0.0%  (-1.4) |
| Computer science | 45.5%  (2.3) | 13.6%  (-0.5) | 27.3%  (0.3) | 36.4%  (-0.2) | 27.3%  (1.1) | 18.2%  (0.7) | 13.6%  (-0.2) | 36.4%  (1.1) | 0.0%  (-1.6) | 18.2%  (-2.4)* | 0.0%  (-1.9) |
| Ecology | 28.6%  (1.1) | 23.5%  (1.8) | 16.8%  (-2.2) | 48.7%  (2.5)* | 15.1%  (-0.9) | 5.0%  (-2.9)* | 10.1%  (-1.7) | 18.5%  (-2.1)* | 10.1%  (0.0) | 48.7%  (1.2) | 11.8%  (-0.5) |
| Education | 26.7%  (0.2) | 13.3%  (-0.5) | 26.7%  (0.2) | 20.0%  (-1.5) | 13.3%  (-0.5) | 46.7%  (3.9)* | 33.3%  (2.0) | 73.3%  (4.2)* | 13.3%  (0.4) | 13.3%  (-2.4)* | 20.0%  (0.8) |
| Engineering | 17.6%  (-1.0) | 20.6%  (0.4) | 41.2%  (2.3) | 29.4%  (-1.1) | 20.6%  (0.4) | 17.6%  (0.8) | 20.6%  (0.9) | 29.4%  (0.5) | 14.7%  (0.9) | 35.3%  (-1.0) | 8.8%  (-0.8) |
| Environmental science | 26.9%  (0.5) | 24.7%  (1.8) | 18.3%  (-1.6) | 50.5%  (2.5)* | 15.1%  -0.8 | 12.9%  (-0.1) | 15.1%  (0.0) | 24.7%  (-0.3) | 6.5%  (-1.2) | 44.1%  (0.1) | 23.7%  (3.2) |
| Geology | 20.0%  (-0.4) | 6.7%  (-1.1) | 20.0%  (-0.4) | 20.0%  (-1.5) | 26.7%  (0.9) | 0.0%  (-1.5) | 0.0%  (-1.7) | 33.3%  (0.6) | 13.3%  (0.4) | 46.7%  (0.2) | 13.3%  (0.0) |
| Hydrology | 21.4%  (-0.4) | 14.3%  (-0.5) | 7.1%  (-2.2) | 42.9%  (0.5) | 25.0%  (1.0) | 10.7%  (-0.4) | 14.3%  (-0.1) | 25.0%  (-0.1) | 10.7%  (0.1) | 53.6%  (1.1) | 10.7%  (-0.4) |
| Information science | 17.6%  (-1.2) | 15.7%  (-0.4) | 29.4%  (0.8) | 43.1%  (0.7) | 23.5%  (1.1) | 15.7%  (0.6) | 13.7%  (-0.3) | 27.5%  (0.2) | 2.0%  (-2.0) | 23.5%  (-3.0)* | 7.8%  (-1.2) |
| Law | 0.0%  (-1.0) | 0.0%  (-0.8) | 33.3%  (0.3) | 0.0%  (-1.4) | 0.0%  (-0.8) | 0.0%  (-0.7) | 0.0%  (-0.7) | 0.0%  (-1.0) | 0.0%  (-0.6) | 0.0%  (-1.5) | 33.3%  (1.0) |
| Medicine/Health Sciences | 11.1%  (-1.7) | 7.4%  (-1.5) | 29.6%  (0.6) | 22.2%  (-1.8) | 33.3%  (2.1) | 29.6%  (2.6)* | 22.2%  (1.0) | 59.3%  (4.0)* | 25.9%  (2.8) | 51.9%  (0.9) | 18.5%  (0.8) |
| Physical sciences | 29.0%  (0.6) | 22.6%  (0.7) | 41.9%  (2.3) | 41.9%  (0.4) | 16.1%  (-0.3) | 16.1%  (0.5) | 22.6%  (1.2) | 16.1%  (-1.3) | 6.5%  (-0.7) | 61.3%  (2.0)* | 16.1%  (0.5) |
| Psychology | 12.5%  (-1.1) | 12.5%  (-0.6) | 18.8%  -0.6 | 6.3%  (-2.7)* | 25.0%  (0.7) | 31.3%  (2.2)* | 0.0%  (-1.7) | 25.0%  (-0.1) | 18.8%  (1.2) | 37.5%  (-0.5) | 12.5%  (-0.1) |
| Social sciences | 25.0%  (0.0) | 21.9%  (0.6) | 28.1%  (0.4) | 43.8%  (0.6) | 18.8%  (0.1) | 6.3%  (-1.2) | 25.0%  (1.6) | 40.6%  (1.9) | 9.4%  (-0.1) | 46.9%  (0.4) | 3.1%  (-1.7) |
| Other (please specify) | 27.8%  (0.4) | 13.9%  (-0.6) | 33.3%  (1.2) | 36.1%  (-0.3) | 13.9%  (-0.7) | 19.4%  (1.2) | 22.2%  (1.2) | 16.7%  (-1.3) | 8.3%  (-0.4) | 33.3%  (-1.3) | 22.2%  (1.6) |
| Humanities | 14.3%  (-0.6) | 0.0%  (-1.2) | 14.3%  (-0.6) | 28.6%  (-0.5) | 0.0%  (-1.2) | 14.3%  (0.1) | 14.3%  (-0.1) | 14.3%  (-0.7) | 42.9%  (2.9) | 28.6%  (-0.8) | 0.0%  (-1.0) |
| Total | 24.6% | 17.9% | 24.8% | 38.6% | 18.1% | 13.1% | 15.2% | 26.1% | 10.1% | 43.6% | 13.3% |
| ***X^2^; p***** | *X^2^=*15.83  P= .602 | *X^2^*=18.86  P= .401 | *X^2^*=25.11  P=.115 | *X^2^*=37.75  P=.004 | *X^2^*=25.73  P=.105 | *X^2^*=41.35  P=.002 | *X^2^*=23.24  P=.178 | *X^2^*=50.04  P<.001 | *X^2^*=29.02  P=.052 | *X^2^*=44.54  P<.001 | *X^2^*=28.36  P=.060 |

Table shows percentages for each chosen barrier (“yes”) within each subject discipline. Chi-square tests for each barrier are reported. Adjusted standardized residuals are reported beneath each percentage, with those greater than 2.0 and less than -2.0 indicating significant deviation from expected cell values (the total).

** Significant difference from expected value*

** Monte Carlo’s test of significance used due to possible small cell sizes*

Table MM. Conditions for use of data by subject discipline

|  | Co-authorship on publications resulting from use of the data | | | Acknowledgment of the data providers in all disseminated work | | | Citation of data providers on all disseminated work making use of data | | |
| --- | --- | --- | --- | --- | --- | --- | --- | --- | --- |
|  | yes | no | not sure | yes | no | not sure | yes | no | not sure |
| Agriculture  and Natural  Resources | 45.9%  (1.6) | 24.6%  (-1.6) | 29.5%  (-0.1) | 87.1%  -(0.2) | 4.8%  (-0.2) | 8.1%  (0.4) | 83.9%  (-0.3) | 9.7%  (0.0) | 9.7%  (0.4) |
| Atmospheric science | 50.0%  (1.9) | 25.0%  (-1.2) | 25.0%  (-0.7) | 94.9%  (1.4) | 2.6%  (-0.8) | 2.6%  (-1.1) | 100.0%  (2.7)* | 0.0%  (-1.7) | 0.0%  (-2.0)* |
| Biology | 37.5%  (0.2) | 43.8%  (1.5) | 18.8%  (-1.8) | 89.6%  (0.4) | 2.1%  (-1.1) | 8.3%  (0.4) | 93.8%  (1.7) | 2.1%  (-0.7) | 2.1%  (-1.6) |
| Business | 41.7%  (0.4) | 33.3%  (0.0) | 25.0%  (-0.4) | 66.7%  (-2.2)* | 25.0%  (3.0)* | 8.3%  (0.2) | 66.7%  (-1.8) | 8.3%  (2.6)* | 8.3%  (0.0) |
| Computer science | 38.1%  (0.2) | 42.9%  (0.9) | 19.0%  (-1.1) | 90.9%  (0.5) | 0.0%  (-1.1) | 9.1%  (0.4) | 90.9%  (0.8) | 4.5%  (-0.4) | 4.5%  (-0.7) |
| Ecology | 30.3%  (-1.6) | 29.5%  (-1.1) | 40.2%  (2.8)* | 91.4%  (1.4) | 4.7%  (-0.4) | 3.9%  (-1.4) | 86.0%  (0.3) | 8.5%  (-0.5) | 8.5%  (0.0) |
| Education | 30.8%  (-0.4) | 46.2%  (1.0) | 23.1%  (-0.6) | 84.6%  (-0.3) | 7.7%  (0.4) | 7.7%  (0.1) | 69.2%  (-1.6) | 15.4%  (1.3) | 15.4%  (0.9) |
| Engineering | 38.7%  (0.3) | 38.7%  (0.6) | 22.6%  (-0.9) | 83.3%  (-0.8) | 13.3%  (1.9) | 3.3%  (-0.8) | 74.2%  (-1.7) | 9.7%  (2.2)* | 9.7%  (0.3) |
| Environmental science | 37.0%  (0.2) | 28.0%  (-1.3) | 35.0%  (1.2) | 95.0%  (2.4)* | 2.0%  (-1.7) | 3.0%  (-1.6) | 86.1%  (0.3) | 8.9%  (-0.7) | 8.9%  (0.2) |
| Geology | 60.0%  (1.9) | 20.0%  (-1.1) | 20.0%  (-0.9) | 93.3%  (0.7) | 0.0%  (-0.9) | 6.7%  (0.0) | 93.3%  (0.9) | 6.7%  (-1.0) | 6.7%  (-0.3) |
| Hydrology | 51.7%  (1.8) | 31.0%  (-0.3) | 17.2%  (-1.5) | 93.1%  (0.9) | 3.4%  (-0.5) | 3.4%  (-0.7) | 86.2%  (0.2) | 10.3%  (-0.7) | 10.3%  (0.4) |
| Information science | 22.2%  (-2.0)* | 55.6%  (3.2)* | 22.2%  (-1.2) | 83.7%  (-0.8) | 14.0%  (2.5)* | 2.3%  (-1.2) | 86.4%  (0.2) | 9.1%  (-0.5) | 9.1%  (0.2) |
| Law | 33.3%  (-0.1) | 66.7%  (1.2) | 0.0%  (-1.1) | 33.3%  (-2.9)* | 33.3%  (2.1)* | 33.3%  (1.8) | 33.3%  (-2.5)* | 0.0%  (4.3)* | 0.0%  (-0.5) |
| Medicine/Health Sciences | 38.5%  (0.2) | 11.5%  (-2.4)* | 50.0%  (2.3)* | 76.9%  (-1.7) | 0.0%  (-1.3) | 23.1%  (3.4)* | 73.1%  (-1.8) | 26.9%  (-1.4) | 26.9%  (3.5)* |
| Physical sciences | 32.3%  (-0.5) | 38.7%  (0.6) | 29.0%  (-0.1) | 81.3%  (-1.1) | 6.3%  (0.2) | 12.5%  (1.3) | 90.6%  (0.9) | 6.3%  (-0.8) | 6.3%  (-0.5) |
| Psychology | 53.8%  (1.3) | 46.2%  (1.0) | 0.0%  (-2.4)* | 84.6%  (-0.3) | 7.7%  (0.4) | 7.7%  (0.1) | 76.9%  (-0.8) | 7.7%  (1.3) | 7.7%  (-0.1) |
| Social sciences | 17.1%  (-2.4)* | 54.3%  (2.6)* | 28.6%  (-0.2) | 77.8%  (-1.9) | 13.9%  (2.3)* | 8.3%  (0.4) | 77.8%  (-1.3) | 8.3%  (1.9) | 8.3%  (0.0) |
| Other (please specify) | 28.6%  (-1.0) | 28.6%  (-0.7) | 42.9%  (1.7) | 83.3%  (-0.8) | 2.8%  (-0.7) | 13.9%  (1.7) | 83.8%  (-0.2) | 8.1%  (0.4) | 8.1%  (-0.1) |
| Humanities | 0.8%  (-0.1) | 0.9%  (0.0) | 1.0%  (0.2) | 83.3%  (-0.3) | 0.0%  -0.6 | 16.7%  (1.0) | 83.3%  (-0.1) | 16.7%  (-0.6) | 16.7%  (0.7) |
| Total | 36.2% | 33.8% | 30.0% | 87.7% | 5.5% | 6.8% | 85.1% | 8.5% | 8.5% |
| ***X^2^; p***** | *X^2^* =64.34  *p=*.002 | | | *X^2^* = 65.04  P= .006 | | | *X^2^* = 65.46  P= .005 | | |

Table MM., continued

|  | Opportunity to collaborate on a project using the data | | | Results based on data not disseminated without data provider’s approval | | | At least part of costs of data acquisition, retrieval, or provision must be recovered | | |
| --- | --- | --- | --- | --- | --- | --- | --- | --- | --- |
|  | yes | no | not sure | yes | no | not sure | yes | no | not sure |
| Agriculture  and Natural  Resources | 67.2%  (1.4) | 13.1%  (-1.3) | 19.7%  (-0.4) | 42.6%  (2.5)* | 39.3%  (-1.9) | 18.0%  (-0.4) | 14.8%  (0.1) | 59.0%  (-0.6) | 26.2%  (0.6) |
| Atmospheric science | 69.2%  (1.4) | 15.4%  (-0.7) | 15.4%  (-1.0) | 33.3%  (0.6) | 38.5%  (-1.6) | 28.2%  (1.3) | 17.9%  (0.7) | 64.1%  (0.2) | 17.9%  (-0.8) |
| Biology | 52.1%  (-1.0) | 18.8%  (-0.2) | 29.2%  (1.3) | 21.3%  (-1.2) | 61.7%  (1.5) | 17.0%  (-0.6) | 14.6%  (0.1) | 62.5%  (0.0) | 22.9%  (0.0) |
| Business | 58.3%  (0.0) | 25.0%  (0.5) | 16.7%  (-0.4) | 41.7%  (1.0) | 41.7%  (-0.6) | 16.7%  (-0.3) | 58.3%  (4.4)* | 41.7%  (-1.5) | 0.0%  (-1.9) |
| Computer science | 68.2%  (0.9) | 18.2%  (-0.2) | 13.6%  (-0.9) | 31.8%  (0.3) | 54.5%  (0.4) | 13.6%  (-0.8) | 19.0%  (0.6) | 52.4%  (-1.0) | 28.6%  (0.6) |
| Ecology | 63.1%  (1.1) | 17.7%  (-0.6) | 19.2%  (-0.7) | 17.8%  (-3.1)* | 58.9%  (2.0) | 23.3%  (1.0) | 5.4%  (-3.2)* | 73.6%  (2.9)* | 20.9%  (-0.7) |
| Education | 15.4%  (-3.2)* | 30.8%  (1.0) | 53.8%  (2.8)* | 30.8%  (0.1) | 38.5%  (-0.9) | 30.8%  (1.0) | 18.2%  (0.4) | 63.6%  (0.1) | 18.2%  (-0.4) |
| Engineering | 61.3%  (0.3) | 19.4%  (0.0) | 19.4%  (-0.3) | 35.5%  (0.8) | 41.9%  (-1.0) | 22.6%  (0.3) | 29.0%  (2.4)* | 48.4%  (-1.7) | 22.6%  (-0.1) |
| Environmental science | 67.3%  (1.9) | 11.9%  (-2.1)* | 20.8%  (-0.2) | 27.7%  (-0.3) | 53.5%  (0.6) | 18.8%  (-0.4) | 14.0%  (-0.1) | 66.0%  (0.7) | 20.0%  (-0.8) |
| Geology | 80.0%  (1.7) | 13.3%  (-0.6) | 6.7%  (-1.4) | 40.0%  (0.9) | 53.3%  (0.2) | 6.7%  (-1.3) | 21.4%  (0.8) | 64.3%  (0.1) | 14.3%  (-0.8) |
| Hydrology | 58.6%  (0.0) | 17.2%  (-0.3) | 24.1%  (0.3) | 37.9%  (1.1) | 34.5%  (-1.8) | 27.6%  (1.0) | 10.3%  (-0.6) | 72.4%  (1.1) | 17.2%  (-0.8) |
| Information science | 45.5%  (-1.8) | 38.6%  (3.3)* | 15.9%  (-1.0) | 15.9%  (-2.0) | 65.9%  (2.1) | 18.2%  (-0.3) | 11.4%  (-0.6) | 75.0%  (1.7) | 13.6%  (-1.5) |
| Law | 33.3%  (-0.9) | 66.7%  (2.1) | 0.0%  -0.9 | 0.0%  (-1.1) | 66.7%  (0.6) | 33.3%  (0.6) | 0.0%  (-0.7) | 66.7%  (0.1) | 33.3%  (0.4) |
| Medicine/Health Sciences | 53.8%  (-0.5) | 7.7%  (-1.6) | 38.5%  (2.1)* | 48.0%  (2.1)* | 24.0%  (-2.7)* | 28.0%  (1.0) | 30.8%  (2.5)* | 23.1%  (-4.3)* | 46.2%  (2.8)* |
| Physical sciences | 34.4%  (-2.9)* | 37.5%  (2.6)* | 28.1%  (0.9) | 21.9%  (-0.9) | 62.5%  (1.4) | 15.6%  (-0.7) | 15.6%  (0.2) | 65.6%  (0.4) | 18.8%  (-0.6) |
| Psychology | 69.2%  (0.8) | 7.7%  (-1.1) | 23.1%  (0.1) | 61.5%  (2.6)* | 38.5%  (-0.9) | 0.0%  (-1.8) | 15.4%  (0.1) | 30.8%  (-2.4)* | 53.8%  (2.6) |
| Social sciences | 37.1%  (-2.7)* | 42.9%  (3.5)* | 20.0%  (-0.2) | 20.0%  (-1.2) | 62.9%  (1.5) | 17.1%  (-0.5) | 8.6%  (-1.0) | 71.4%  (1.1) | 20.0%  (-0.5) |
| Other (please specify) | 64.9%  (0.8) | 10.8%  (-1.4) | 24.3%  (0.4) | 38.9%  (1.3) | 41.7%  (-1.1) | 19.4%  (-0.1) | 5.4%  (-1.6) | 54.1%  (-1.1) | 40.5%  (2.6)* |
| Humanities | 33.3%  (-1.3) | 33.3%  (0.8) | 33.3%  (0.7) | 33.3%  (0.2) | 33.3%  (-0.9) | 33.3%  (0.8) | 16.7%  (0.2) | 33.3%  (-1.5) | 50.0%  (1.6) |
| Total | 58.7% | 19.7% | 21.7% | 29.0% | 50.8% | 20.2% | 14.2% | 62.7% | 23.2% |
| ***X^2^; p***** | *X^2^* = 72.77  P<.001 | | | *X^2^* = 55.19  P= .021 | | | *X^2^* = 81.16  P< .001 | | |

Table MM., continued

|  | Data provider has opportunity to review results and make suggestions and comments | | | Reprints of articles that make use of data must be provided to data provider | | | Data provider is given a complete list of all products that make use of the data | | |
| --- | --- | --- | --- | --- | --- | --- | --- | --- | --- |
|  | yes | no | not sure | yes | no | not sure | yes | no | not sure |
| Agriculture  and Natural  Resources | 49.2%  (2.1)* | 32.8%  (-1.6) | 18.0%  (-0.5) | 50.0%  (0.5) | 30.0%  (-1.4) | 20.0%  (1.2) | 50.8%  (1.2) | 34.4%  (-0.5) | 14.8%  (-0.8) |
| Atmospheric science | 41.0%  (0.6) | 43.6%  (0.1) | 15.4%  (-0.8) | 52.6%  (0.7) | 34.2%  (-0.6) | 13.2%  (-0.3) | 41.0%  (-0.4) | 30.8%  (-0.9) | 28.2%  (1.6) |
| Biology | 33.3%  (-0.5) | 39.6%  (-0.4) | 27.1%  (1.2) | 43.8%  (-0.5) | 52.1%  (2.0)* | 4.2%  (-2.1)* | 37.5%  (-0.9) | 43.8%  (0.9) | 18.8%  (0.0) |
| Business | 33.3%  (-0.3) | 50.0%  (0.5) | 16.7%  (-0.3) | 41.7%  (-0.4) | 58.3%  (1.4) | 0.0%  (-1.4) | 41.7%  (-0.2) | 50.0%  (0.9) | 8.3%  (-0.9) |
| Computer science | 13.6%  (-2.3)* | 63.6%  (2.0)* | 22.7%  (0.3) | 27.3%  (-1.9) | 54.5%  (1.6) | 18.2%  (0.5) | 31.8%  (-1.2) | 40.9%  (0.3) | 27.3%  (1.1) |
| Ecology | 45.0%  (2.1)* | 36.4%  (-1.5) | 18.6%  (-0.6) | 55.5%  (2.2)* | 32.8%  (-1.5) | 11.7%  (-1.0) | 48.9%  (1.3) | 37.4%  (0.0) | 13.7%  (-1.6) |
| Education | 7.7%  (-2.2)* | 46.2%  (0.3) | 46.2%  (2.3)* | 23.1%  (-1.7) | 46.2%  (0.6) | 30.8%  (1.7) | 53.8%  (0.7) | 23.1%  (-1.1) | 23.1%  (0.4) |
| Engineering | 25.8%  (-1.3) | 51.6%  (1.0) | 22.6%  (0.3) | 58.1%  (1.3) | 32.3%  (-0.7) | 9.7%  (-0.8) | 33.3%  (-1.2) | 33.3%  (-0.5) | 33.3%  (2.1) |
| Environmental science | 47.5%  (2.4)* | 36.6%  (-1.3) | 15.8%  (-1.3) | 52.0%  (1.1) | 29.0%  (-2.1)* | 19.0%  (1.3) | 47.5%  (0.8) | 33.7%  (-0.9) | 18.8%  (0.0) |
| Geology | 57.1%  (1.6) | 42.9%  (0.0) | 0.0%  (-1.9) | 50.0%  (0.2) | 42.9%  (0.3) | 7.1%  (-0.8) | 50.0%  (0.5) | 35.7%  (-0.1) | 14.3%  (-0.4) |
| Hydrology | 34.5%  (-0.3) | 34.5%  (-0.9) | 31.0%  (1.4) | 62.1%  (1.7) | 24.1%  (-1.6) | 13.8%  (-0.1) | 55.2%  (1.3) | 27.6%  (-1.1) | 17.2%  (-0.2) |
| Information science | 20.5%  (-2.3)* | 61.4%  (2.6)* | 18.2%  (-0.4) | 36.4%  (-1.4) | 47.7%  (1.3) | 15.9%  (0.3) | 43.2%  (-0.1) | 40.9%  (0.5) | 15.9%  (-0.5) |
| Law | 33.3%  (-0.1) | 66.7%  (0.8) | 0.0%  (-0.9) | 0.0%  (-1.6) | 100.0%  (2.2)* | 0.0%  (-0.7) | 33.3%  (-0.4) | 66.7%  (1.0) | 0.0%  (-0.8) |
| Medicine/Health Sciences | 34.6%  (-0.2) | 34.6%  (-0.8) | 30.8%  (1.3) | 40.7%  (-0.7 | 25.9%  (-1.4) | 33.3%  (2.8)* | 46.2%  (0.2) | 23.1%  (-1.5) | 30.8%  (1.6) |
| Physical sciences | 31.3%  (-0.7) | 62.5%  (2.3)* | 6.3%  (-2.1)* | 32.3%  (-1.7) | 64.5%  (3.0)* | 3.2%  (-1.8) | 12.5%  (-3.7) | 59.4%  (2.6) | 28.1%  (1.4) |
| Psychology | 30.8%  (-0.5) | 46.2%  (0.3) | 23.1%  (0.2) | 53.8%  (0.5) | 38.5%  (0.0) | 7.7%  (-0.7) | 61.5%  (1.3) | 30.8%  (-0.5) | 7.7%  (-1.0) |
| Social sciences | 26.5%  (-1.3) | 52.9%  (1.3) | 20.6%  (0.0) | 25.7%  (-2.6)* | 54.3%  (2.0)* | 20.0%  (0.9) | 28.6%  (-1.9) | 51.4%  (1.7) | 20.0%  (0.2) |
| Other (please specify) | 29.7%  (-0.9) | 35.1%  (-0.9) | 35.1%  (2.2)* | 45.9%  (-0.1) | 37.8%  (-0.1) | 16.2%  (0.3) | 51.4%  (0.9) | 37.8%  (0.0) | 10.8%  (-1.3) |
| Humanities | 16.7%  (-1.0) | 33.3%  (-0.5) | 50.0%  (1.8) | 50.0%  (0.2) | 33.3%  (-0.3) | 16.7%  (0.1) | 50.0%  (0.3) | 33.3%  (-0.2) | 16.7%  (-0.1) |
| Total | 36.9% | 42.5% | 20.6% | 46.9% | 38.5% | 14.6% | 43.8% | 37.5% | 18.7% |
| ***X^2^; p***** | *X^2^* = 62.30  P= .004 | | | *X^2^* = 64.15  P= .003 | | | *X^2^* = 43.53  P= .177 | | |

Table MM., continued

|  | Legal permission for use of data is obtained | | | Mutual agreement on reciprocal sharing of data | | | Data provider is given a statement of uses | | |
| --- | --- | --- | --- | --- | --- | --- | --- | --- | --- |
|  | yes | no | not sure | yes | no | not sure | yes | no | not sure |
| Agriculture  and Natural  Resources | 31.1%  (-0.4) | 41.0%  (-0.7) | 27.9%  (1.4) | 50.8%  (0.8) | 29.5%  (-0.8) | 19.7%  (0.0) | 50.0%  (0.9) | 23.3%  (-1.6) | 26.7%  (0.7) |
| Atmospheric science | 30.8%  (-0.4) | 51.3%  (0.8) | 17.9%  (-0.5) | 46.2%  (0.0) | 38.5%  (0.6) | 15.4%  (-0.7) | 53.8%  (1.2) | 30.8%  (-0.2) | 15.4%  (-1.2) |
| Biology | 22.9%  (-1.6) | 56.3%  (1.6) | 20.8%  (-0.1) | 36.2%  (-1.4) | 44.7%  (1.6) | 19.1%  (-0.1) | 31.9%  (-1.8) | 40.4%  (1.2) | 27.7%  (0.8) |
| Business | 41.7%  (0.6) | 33.3%  (-0.8) | 25.0%  (0.3) | 66.7%  (1.4) | 25.0%  (-0.7) | 8.3%  (-1.0) | 66.7%  (1.6) | 25.0%  (-0.6) | 8.3%  (-1.2) |
| Computer science | 52.4%  (1.9) | 23.8%  (-2.0)* | 23.8%  (0.3) | 45.5%  (-0.1) | 31.8%  (-0.2) | 22.7%  (0.4) | 36.4%  (-0.8) | 40.9%  (0.8) | 22.7%  (0.0) |
| Ecology | 22.0%  (-3.0)* | 59.1%  (3.4)* | 18.9%  (-0.7) | 43.4%  (-0.7) | 38.8%  (1.2) | 17.8%  (-0.6) | 37.5%  (-1.7) | 36.7%  (1.1) | 25.8%  (0.8) |
| Education | 53.8%  (1.6) | 30.8%  (-1.1) | 15.4%  (-0.5) | 30.8%  (-1.1) | 23.1%  (-0.9) | 46.2%  (2.4)* | 38.5%  (-0.4) | 23.1%  (-0.7) | 38.5%  (1.3) |
| Engineering | 35.5%  (0.2) | 38.7%  (-0.8) | 25.8%  (0.7) | 53.3%  (0.8) | 26.7%  (-0.9) | 20.0%  (0.0) | 46.7%  (0.3) | 30.0%  (-0.3) | 23.3%  (0.0) |
| Environmental science | 31.7%  (-0.4( | 45.5%  (0.0) | 22.8%  (0.4) | 52.5%  (1.4) | 22.8%  (-2.6)* | 24.8%  (1.4) | 49.5%  (1.1) | 24.8%  (-1.8) | 25.7%  (0.7) |
| Geology | 28.6%  (-0.4) | 71.4%  (2.0)* | 0.0%  (-2.0)* | 78.6%  (2.5)* | 7.1%  (-2.2)* | 14.3%  (-0.5) | 57.1%  (1.0) | 28.6%  (-0.3) | 14.3%  (-0.8) |
| Hydrology | 44.8%  (1.3) | 37.9%  (-0.8) | 17.2%  (-0.5) | 55.2%  (1.0) | 17.2%  (-2.0)* | 27.6%  (1.1) | 55.2%  (1.2) | 20.7%  (-1.4) | 24.1%  (0.1) |
| Information science | 38.6%  (0.8) | 40.9%  (-0.6) | 20.5%  (-0.1) | 34.9%  (-1.5) | 46.5%  (1.8) | 18.6%  (-0.2) | 47.7%  (0.5) | 34.1%  (0.2) | 18.2%  (-0.8) |
| Law | 33.3%  (0.0) | 33.3%  (-0.4) | 33.3%  (0.5) | 33.3%  (-0.4) | 66.7%  (1.2) | 0.0%  (-0.9) | 33.3%  (-0.4) | 66.7%  (1.3) | 0.0%  (-1.0) |
| Medicine/Health Sciences | 50.0%  (1.8) | 19.2%  (-2.7)* | 30.8%  (1.2) | 53.8%  (0.8) | 11.5%  (-2.5)* | 34.6%  (2.0)* | 65.4%  (2.2) | 7.7%  (-2.8) | 26.9%  (0.5) |
| Physical sciences | 34.4%  (0.1) | 50.0%  (0.5) | 15.6%  (-0.8) | 38.7%  (-0.9) | 51.6%  (2.1)* | 9.7%  (-1.4) | 28.1%  (-1.9) | 53.1%  (2.5) | 18.8%  (-0.6) |
| Psychology | 76.9%  (3.4)* | 7.7%  (-2.8)* | 15.4%  (-0.5) | 46.2%  (0.0) | 46.2%  (0.9) | 7.7%  (-1.1) | 61.5%  (1.3) | 30.8%  (-0.1) | 7.7%  (-1.3) |
| Social sciences | 40.0%  (0.8) | 51.4%  (0.7) | 8.6%  (-1.9) | 28.6%  (-2.1)* | 54.3%  (2.6)* | 17.1%  (-0.4) | 34.3%  (-1.2) | 48.6%  (2.1) | 17.1%  (-0.9) |
| Other (please specify) | 27.0%  (-0.8) | 37.8%  (-1.0) | 35.1%  (2.1)* | 51.4%  (0.7) | 35.1%  (0.1) | 13.5%  (-1.0) | 35.1%  (-1.2) | 37.8%  (0.7) | 27.0%  (0.6) |
| Humanities | 40.0%  (0.3) | 40.0%  (-0.2) | 20.0%  (-0.1) | 33.3%  (-0.6) | 50.0%  (0.8) | 16.7%  (-0.2) | 33.3%  (-0.5) | 50.0%  (0.9) | 16.7%  (-0.4) |
| Total | 33.4% | 45.4% | 21.1% | 46.2% | 34.2% | 19.7% | 44.3% | 32.6% | 23.2% |
| ***X^2^; p***** | *X^2^* = 58.96  P= .009 | | | *X^2^* = 56.59  P= .015 | | | *X^2^* = 45.69  P= .126 | | |

Table shows percentages within each subject disciplines for proposed condition (“yes,” “no,” and “not sure”). Probability values correspond to results of Chi-square tests for each condition. Adjusted standardized residuals are reported beneath each percentage, with those greater than 2.0 and less than -2.0 indicating significant deviation from expected cell values (the total). Chi-square values reported in results.

** Significant difference from expected value*

*** Monte Carlo’s test of significance used due to possible small cell counts.*

Table NN. Amount of data made available to others by subject discipline

|  | **Mean** | **SD** |
| --- | --- | --- |
| Agriculture  and Natural  Resources | 2.55 | 0.82 |
| Atmospheric science | 2.82 | 0.77 |
| Biology | 2.59 | 0.95 |
| Business | 2.00 | 0.91 |
| Computer science | 2.42 | 0.81 |
| Ecology | 2.69 | 0.84 |
| Education | 2.31 | 0.70 |
| Engineering | 2.28 | 0.70 |
| Environmental science | 2.67 | 0.84 |
| Geology | 2.53 | 0.84 |
| Hydrology | 2.46 | 0.89 |
| Information science | 2.32 | 0.90 |
| Law | 2.67 | 0.58 |
| Medicine/Health Sciences | 2.24 | 0.74 |
| Physical sciences | 2.39 | 0.84 |
| Psychology | 2.16 | 0.96 |
| Social sciences | 2.49 | 1.03 |
| Other (please specify) | 2.63 | 0.87 |
| Humanities | 2.50 | 1.27 |

Table shows the means and standard deviations for each subject discipline as they determine the results of a one-way analysis of variance (ANOVA) with amount of data made available to others as the dependent variable, F(18) = 1.99, *p* = .008. There were no significant pairwise differences.

Table OO. Data accessibility by subject discipline

|  |  | **Mean** | **SD** | ***F; p*** |
| --- | --- | --- | --- | --- |
| I share my data with others. | Agriculture and Natural Resources | 4.02 | 0.87 | F= 2.55  P<.000 |
|  | Atmospheric science | 4.41_a_ | 0.55 |  |
|  | Biology | 4.16 | 1.11 |  |
|  | Business | 3.33 | 1.67 |  |
|  | Computer science | 4.04 | 1.19 |  |
|  | Ecology | 4.29_a_ | 0.94 |  |
|  | Education | 3.60 | 1.12 |  |
|  | Engineering | 3.86 | 1.15 |  |
|  | Environmental science | 4.18 | 0.89 |  |
|  | Geology | 3.88 | 1.03 |  |
|  | Hydrology | 4.07 | 1.02 |  |
|  | Information science | 3.85 | 1.37 |  |
|  | Law | 2.50 | 2.12 |  |
|  | Medicine/Health Sciences | 3.55 | 0.91 |  |
|  | Physical sciences | 3.97 | 1.20 |  |
|  | Psychology | 3.25 | 1.53 |  |
|  | Social sciences | 3.83 | 1.16 |  |
|  | Other (please specify) | 3.98 | 1.12 |  |
|  | Humanities | 4.00 | 1.00 |  |
|  |  |  |  |  |
| Others need my permission to access my data. | Agriculture and Natural Resources | 3.69 | 1.31 | F= 1.73  P= .031 |
|  | Atmospheric science | 3.56 | 1.48 |  |
|  | Biology | 3.20 | 1.44 |  |
|  | Business | 4.33 | 1.23 |  |
|  | Computer science | 3.30 | 1.26 |  |
|  | Ecology | 3.55 | 1.24 |  |
|  | Education | 4.07 | 1.03 |  |
|  | Engineering | 3.43 | 1.45 |  |
|  | Environmental science | 3.74 | 1.21 |  |
|  | Geology | 3.88 | 1.36 |  |
|  | Hydrology | 3.60 | 1.13 |  |
|  | Information science | 3.24 | 1.43 |  |
|  | Law | 3.50 | 2.12 |  |
|  | Medicine/Health Sciences | 4.23 | 1.11 |  |
|  | Physical sciences | 3.50 | 1.41 |  |
|  | Psychology | 4.25 | 1.24 |  |
|  | Social sciences | 3.86 | 1.20 |  |
|  | Other (please specify) | 3.45 | 1.41 |  |
|  | Humanities | 2.86 | 1.68 |  |
|  |  |  |  |  |
| Others can access my data easily. | Agriculture and Natural Resources | 3.34 | 1.18 | F= 2.78  P<.001 |
|  | Atmospheric science | 3.41 | 1.33 |  |
|  | Biology | 3.10 | 1.46 |  |
|  | Business | 2.25 | 1.60 |  |
|  | Computer science | 3.30 | 1.36 |  |
|  | Ecology | 3.29 | 1.26 |  |
|  | Education | 2.47 | 1.25 |  |
|  | Engineering | 2.57 | 1.40 |  |
|  | Environmental science | 3.29 | 1.39 |  |
|  | Geology | 2.81 | 1.28 |  |
|  | Hydrology | 3.07 | 0.98 |  |
|  | Information science | 3.28 | 1.41 |  |
|  | Law | 2.50 | 2.12 |  |
|  | Medicine/Health Sciences | 2.23 | 1.19 |  |
|  | Physical sciences | 2.72 | 1.49 |  |
|  | Psychology | 2.06 | 1.18 |  |
|  | Social sciences | 2.72 | 1.37 |  |
|  | Other (please specify) | 2.95 | 1.22 |  |
|  | Humanities | 3.43 | 1.51 |  |
|  |  |  |  |  |

Table shows mean agreement (1= Disagree strongly, 2= disagree somewhat, 3= neither agree nor disagree, 4= agree somewhat, 5= agree strongly) and standard deviation for each item. MANOVA: F(54, 2041.85) = 1.86, *p* < .001, Wilks’ Lambda= .867, partial eta squared= .047.

Univariate ANOVAs for each item within omnibus MANOVA are reported.

*_a_ = Tukey’s post-hoc analysis shows significant pairwise difference from Psychology*

Table PP. Data storage by subject discipline (excludes Other, Dropbox/Google/Figshare/Cloud, External Hard Disk/Drive Storage, and Other Server because those answers were derived from text included with “other”).

|  | Institutional server | PI’s Server | Dept. Server | Personal Computer | On paper | Discipline-based repos. | Publisher repos. | Other repos. or archive | Institution’s repos. |
| --- | --- | --- | --- | --- | --- | --- | --- | --- | --- |
| Agriculture  and Natural  Resources | 78.7%  (1.8) | 47.9%  (-0.8) | 45.8%  (-0.4) | 95.3%  (0.8) | 74.0%  (1.3) | 31.1%  (0.6) | 26.8%  (1.3) | 23.3%  (-1.3) | 40.5%  (1.1) |
| Atmospheric science | 70.2%  (0.3) | 66.7%  (1.8) | 42.9%  (-0.7) | 89.4%  (-1.0) | 41.7%  (-3.1)* | 40.0%  (1.7) | 12.1%  (-1.1) | 59.0%  (3.7)* | 39.5%  (0.9) |
| Biology | 59.3%  (-1.5) | 55.1%  (0.3) | 37.5%  (-1.6) | 98.2%  (1.6) | 81.1%  (2.5)* | 20.0%  (-1.1) | 18.8%  (-0.1) | 38.3%  (1.0) | 21.7%  (-1.7) |
| Business | 77.8%  (0.6) | 55.6%  (0.1) | 42.9%  (-0.3) | 83.3%  (-1.3) | 44.4%  (-1.3) | 12.5%  (-0.9) | 25.0%  (0.4) | 22.2%  (-0.6) | 25.0%  (-0.5) |
| Computer science | 63.6%  (-0.5) | 59.1%  (0.6) | 65.2%  (1.6) | 76.2%  (-3.0)* | 42.9%  (-2.2)* | 33.3%  (0.6) | 33.3%  (1.7) | 27.3%  (-0.5) | 47.8%  (1.6) |
| Ecology | 61.2%  (-1.8) | 51.4%  (-0.4) | 48.3%  (0.0) | 93.7%  (0.4) | 71.7%  (1.5) | 44.6%  (4.8)* | 22.6%  (1.0) | 28.7%  (-0.8) | 25.2%  (-1.8) |
| Education | 92.3%  (1.9) | 70.0%  (1.1) | 83.3%  (2.4)* | 78.6%  (-2.1)* | 69.2%  (0.3) | 18.2%  (-0.7) | 20.0%  (0.1) | 45.5%  (1.0) | 54.5%  (1.6) |
| Engineering | 71.0%  (0.3) | 66.7%  (1.4) | 70.4%  (2.3)* | 96.8%  (0.9) | 75.0%  (1.0) | 21.1%  (-0.6) | 5.0%  (-1.6) | 31.8%  (0.0) | 31.6%  (-0.1) |
| Environmental science | 67.3%  (-0.2) | 55.6%  (0.5) | 50.5%  (0.4) | 95.4%  (1.1) | 66.3%  (0.2) | 28.7%  (0.3) | 21.7%  (0.6) | 41.9%  (2.1)* | 41.1%  (1.8) |
| Geology | 42.9%  (-2.1) | 54.5%  (0.1) | 44.4%  (-0.2) | 100.0%  (1.2) | 64.3%  (-0.1) | 20.0%  (-0.5) | 11.1%  (-0.6) | 25.0%  (-0.5) | 27.3%  (-0.4) |
| Hydrology | 84.4%  (2.0) | 60.0%  (0.7) | 36.4%  (-1.2) | 96.7%  (0.8) | 50.0%  (-1.6) | 22.7%  (-0.5) | 9.5%  (-1.2) | 23.8%  (-0.8) | 45.5%  (1.3) |
| Information science | 72.0%  (0.6) | 36.8%  (-2.1) | 60.5%  (1.6) | 87.2%  (-1.5) | 50.0%  (-2.2)* | 21.6%  (-0.8) | 20.5%  (0.2) | 26.2%  (-0.8) | 33.3%  (0.1) |
| Law | 100.0%  (1.2) | 0.0%  (-1.5) | 0.0%  (-1.4) | 66.7%  (-1.8) | 50.0%  (-0.5) | 0.0%  (-0.9) | 0.0%  (-0.7) | 0.0%  (-1.0) | 33.3%  (0.0) |
| Medicine/Health Sciences | 83.3%  (1.6) | 66.7%  (1.2) | 53.3%  (0.4) | 90.5%  (-0.4) | 82.4%  (1.5) | 7.7%  (-1.6) | 26.7%  (0.7) | 31.3%  (-0.1) | 41.2%  (0.7) |
| Physical sciences | 63.3%  (-0.6) | 40.0%  (-1.3) | 40.7%  (-0.8) | 97.1%  (1.0) | 60.0%  (-0.7) | 7.1%  (-2.5)* | 18.5%  (-0.1) | 33.3%  (0.2) | 28.0%  (-0.5) |
| Psychology | 60.0%  (-0.7) | 73.3%  (1.6) | 25.0%  (-1.6) | 88.2%  (-0.7) | 68.8%  (0.3) | 7.7%  (-1.6) | 7.7%  (-1.1) | 7.7%  (-1.9) | 8.3%  (-1.8) |
| Social sciences | 64.9%  (-0.4) | 38.9%  (-1.8) | 40.0%  (-1.0) | 87.5%  (-1.3) | 61.5%  (-0.5) | 10.8%  (-2.3)* | 8.1%  (-1.8) | 20.5%  (-1.6) | 29.7%  (-0.4) |
| Other (please specify) | 68.4%  (0.0) | 50.0%  (-0.4) | 51.6%  (0.4) | 95.0%  (0.5) | 68.8%  (0.4) | 27.6%  (0.0) | 22.2%  (0.4) | 33.3%  (0.2) | 14.3%  (-1.8) |
| Humanities | 50.0%  (-1.1) | 33.3%  (-1.0) | 16.7%  (-1.6) | 100.0%  (0.8) | 100.0%  (1.8) | 0.0%  (-1.5) | 16.7%  (-0.2) | 0.0%  (-1.7) | 33.3%  (0.0) |
| Total | 68.2% | 53.1% | 48.4% | 92.8% | 65.5% | 27.3% | 19.3% | 31.9% | 32.8% |
| ***X^2^; p***** | X^2^=26.62  P=.081 | X^2^=23.38  P=.172 | X^2^=29.44  P=.038 | X^2^=32.05  P=.029 | X^2^=39.54  P=.002 | X^2^=44.79  P<.001 | X^2^=16.13  P=.593 | X^2^=32.64  P=.015 | X^2^=24.12  P=.148 |

Table shows percentage within each subject discipline for each storage location (“yes” within dichotomized variable). Chi-square tests for each location are reported. Adjusted standardized residuals are reported beneath each percentage, with those greater than 2.0 and less than -2.0 indicating significant deviation from expected cell values (the total).

** Significant difference from expected value*

*** Monte Carlo’s test of significance used due to possible small cell counts.*

Table QQ. Metadata standards used by subject discipline

|  | Metadata standardized within my institution | Metadata standardized within my lab | None |
| --- | --- | --- | --- |
| Agriculture  and Natural  Resources | 18.3%  (1.2) | 15.9%  (-0.2) | 50.0%  (0.4) |
| Atmospheric science | 17.9%  (0.8) | 19.6%  (0.6) | 28.6%  (-3.0)* |
| Biology | 7.6%  (-1.6) | 21.2%  (1.0) | 54.5%  (1.1) |
| Business | 0.0%  (-1.5) | 15.4%  (-0.1) | 71.4%  (1.8) |
| Computer science | 17.2%  (0.5) | 27.6%  (1.6) | 48.3%  (0.0) |
| Ecology | 9.4%  (-1.9) | 24.4%  (2.9) | 40.6%  (-2.1)* |
| Education | 25.0%  (1.4) | 10.0%  (-0.8) | 55.0%  (0.6) |
| Engineering | 8.9%  (-1.0) | 20.0%  (0.6) | 60.0%  (1.6) |
| Environmental science | 16.4%  (0.8) | 13.3%  (-1.1) | 36.7%  (-2.8)* |
| Geology | 19.0%  (0.7) | 19.0%  (0.3) | 47.6%  (0.0) |
| Hydrology | 18.4%  (0.8) | 15.8%  (-0.1) | 36.8%  (-1.4) |
| Information science | 22.2%  (2.1) | 7.0%  (-2.3) | 37.0%  (-2.0)* |
| Law | 33.3%  (1.0) | 0.0%  (-0.8) | 66.7%  (0.6) |
| Medicine/Health Sciences | 8.1%  (-1.1) | 10.8%  (-1.0) | 64.9%  (2.1)* |
| Physical sciences | 9.5%  (-0.9) | 19.0%  (0.4) | 64.3%  (2.2)* |
| Psychology | 4.8%  (-1.2) | 14.3%  (-0.3) | 76.2%  (2.6)* |
| Social sciences | 16.7%  (0.5) | 2.4%  (-2.5) | 65.9%  (2.4)* |
| Other (please specify) | 10.9%  (-0.6) | 15.2%  (-0.3) | 55.1%  (1.0) |
| Humanities | 1.5%  (1.7) | 20.0%  (0.2) | 87.5%  (2.2)* |
| Total | 14.0% | 16.6% | 48.1% |
| ***X^2^; p***** | X^2^= 25.68  P=.104 | X^2^=25.70  P= .102 | X^2^= 58.68  P<.001 |

Table shows percentages within each subject discipline for 3 metadata standard options (“yes”) that may limit accessibility of data for outside researchers. Chi-square tests for each metadata standard are reported. Adjusted standardized residuals are reported beneath each percentage, with those greater than 2.0 and less than -2.0 indicating significant deviation from expected cell values (the total).

** Significant difference from expected value*

***Monte Carlo’s test of significance used to account for potentially small cell sizes.*

| Table RR. Perceptions of organizational support by subject discipline | | | | |
| --- | --- | --- | --- | --- |
|  |  | **Mean** | **SD** | **F*; p*** |
| managing data during the life of the project (short term) | Agriculture and Natural Resources | 3.22 | 1.544 | F= 1.58  P= .061 |
|  | Atmospheric science | 3.14 | 1.627 |  |
|  | Biology | 2.35 | 1.434 |  |
|  | Business | 2.40 | 1.673 |  |
|  | Computer science | 2.23 | 1.166 |  |
|  | Ecology | 2.87 | 1.542 |  |
|  | Education | 3.33 | 1.118 |  |
|  | Engineering | 2.45 | 1.276 |  |
|  | Environmental science | 2.75 | 1.581 |  |
|  | Geology | 3.40 | 1.776 |  |
|  | Hydrology | 3.06 | 1.552 |  |
|  | Information science | 3.06 | 1.533 |  |
|  | Law | 2.50 | 2.121 |  |
|  | Medicine/Health Sciences | 2.25 | 1.422 |  |
|  | Physical sciences | 2.83 | 1.586 |  |
|  | Psychology | 2.13 | 1.356 |  |
|  | Social sciences | 2.22 | 1.313 |  |
|  | Other (please specify) | 2.09 | 1.276 |  |
|  | Humanities | 5.00 | . |  |
|  |  |  |  |  |
| storing data beyond the life of the project (long term) | Agriculture and Natural Resources | 2.95 | 1.584 | F= 1.23  P= .234 |
|  | Atmospheric science | 3.11 | 1.499 |  |
|  | Biology | 2.30 | 1.396 |  |
|  | Business | 2.40 | 1.673 |  |
|  | Computer science | 2.46 | 1.450 |  |
|  | Ecology | 2.82 | 1.528 |  |
|  | Education | 2.78 | .972 |  |
|  | Engineering | 2.40 | 1.429 |  |
|  | Environmental science | 2.78 | 1.654 |  |
|  | Geology | 3.60 | 1.506 |  |
|  | Hydrology | 3.11 | 1.451 |  |
|  | Information science | 2.89 | 1.549 |  |
|  | Law | 2.50 | 2.121 |  |
|  | Medicine/Health Sciences | 1.92 | 1.505 |  |
|  | Physical sciences | 2.43 | 1.376 |  |
|  | Psychology | 2.00 | 1.414 |  |
|  | Social sciences | 2.65 | 1.668 |  |
|  | Other (please specify) | 2.22 | 1.313 |  |
|  | Humanities | 5.00 | . |  |
|  |  |  |  |  |
| training on best practices for data management. | Agriculture and Natural Resources | 2.40 | 1.277 | F= 1.02  P= .435 |
|  | Atmospheric science | 2.64 | 1.471 |  |
|  | Biology | 2.04 | 1.107 |  |
|  | Business | 2.80 | 1.643 |  |
|  | Computer science | 3.00 | 1.581 |  |
|  | Ecology | 2.43 | 1.473 |  |
|  | Education | 2.56 | .882 |  |
|  | Engineering | 2.50 | 1.504 |  |
|  | Environmental science | 2.49 | 1.422 |  |
|  | Geology | 2.50 | 1.354 |  |
|  | Hydrology | 2.56 | 1.423 |  |
|  | Information science | 3.00 | 1.455 |  |
|  | Law | 3.50 | .707 |  |
|  | Medicine/Health Sciences | 2.42 | 1.621 |  |
|  | Physical sciences | 1.87 | 1.100 |  |
|  | Psychology | 2.00 | 1.414 |  |
|  | Social sciences | 2.52 | 1.377 |  |
|  | Other (please specify) | 2.22 | 1.347 |  |
|  | Humanities | 4.00 | . |  |
|  |  |  |  |  |
| assistance on creating data management plans. | Agriculture and Natural Resources | 2.53 | 1.261 | F= 1.27  P= .206 |
|  | Atmospheric science | 2.54 | 1.527 |  |
|  | Biology | 2.04 | 1.186 |  |
|  | Business | 2.40 | 1.673 |  |
|  | Computer science | 2.85 | 1.345 |  |
|  | Ecology | 2.52 | 1.544 |  |
|  | Education | 2.56 | .882 |  |
|  | Engineering | 2.75 | 1.650 |  |
|  | Environmental science | 2.32 | 1.490 |  |
|  | Geology | 2.80 | 1.549 |  |
|  | Hydrology | 2.78 | 1.555 |  |
|  | Information science | 2.94 | 1.533 |  |
|  | Law | 3.50 | .707 |  |
|  | Medicine/Health Sciences | 1.92 | 1.165 |  |
|  | Physical sciences | 1.78 | 1.204 |  |
|  | Psychology | 2.00 | 1.414 |  |
|  | Social sciences | 2.43 | 1.199 |  |
|  | Other (please specify) | 2.00 | 1.243 |  |
|  | Humanities | 4.00 | . |  |
|  |  |  |  |  |
| assistance on creating metadata to describe my data or datasets. | Agriculture and Natural Resources | 2.22 | 1.209 | F= 1.30  P= .182 |
|  | Atmospheric science | 2.32 | 1.442 |  |
|  | Biology | 1.87 | 1.014 |  |
|  | Business | 2.60 | 1.517 |  |
|  | Computer science | 2.85 | 1.281 |  |
|  | Ecology | 2.51 | 1.544 |  |
|  | Education | 2.11 | .928 |  |
|  | Engineering | 2.55 | 1.317 |  |
|  | Environmental science | 2.46 | 1.500 |  |
|  | Geology | 3.00 | 1.563 |  |
|  | Hydrology | 2.78 | 1.478 |  |
|  | Information science | 2.89 | 1.510 |  |
|  | Law | 3.00 | 1.414 |  |
|  | Medicine/Health Sciences | 1.92 | 1.165 |  |
|  | Physical sciences | 1.83 | 1.435 |  |
|  | Psychology | 1.75 | 1.488 |  |
|  | Social sciences | 2.13 | 1.359 |  |
|  | Other (please specify) | 2.04 | 1.364 |  |
|  | Humanities | 3.00 | . |  |
|  |  |  |  |  |
| training on how to cite datasets. | Agriculture and Natural Resources | 2.25 | 1.214 | F= 1.24  P= .225 |
|  | Atmospheric science | 2.61 | 1.370 |  |
|  | Biology | 2.00 | 1.087 |  |
|  | Business | 2.80 | 1.643 |  |
|  | Computer science | 2.92 | 1.605 |  |
|  | Ecology | 2.39 | 1.329 |  |
|  | Education | 1.89 | .601 |  |
|  | Engineering | 2.55 | 1.504 |  |
|  | Environmental science | 2.54 | 1.483 |  |
|  | Geology | 3.00 | 1.633 |  |
|  | Hydrology | 2.56 | 1.464 |  |
|  | Information science | 2.94 | 1.494 |  |
|  | Law | 2.50 | 2.121 |  |
|  | Medicine/Health Sciences | 2.17 | 1.267 |  |
|  | Physical sciences | 1.91 | 1.276 |  |
|  | Psychology | 2.00 | 1.512 |  |
|  | Social sciences | 2.30 | 1.521 |  |
|  | Other (please specify) | 2.00 | 1.206 |  |
|  | Humanities | 4.00 | . |  |
|  |  |  |  |  |
| ... during the life of a research project (short term) | Agriculture and Natural Resources | 3.33 | 1.309 | F= 1.54  P= .073 |
|  | Atmospheric science | 3.18 | 1.416 |  |
|  | Biology | 2.22 | 1.536 |  |
|  | Business | 3.00 | 1.581 |  |
|  | Computer science | 2.85 | 1.405 |  |
|  | Ecology | 3.10 | 1.544 |  |
|  | Education | 2.33 | 1.225 |  |
|  | Engineering | 2.70 | 1.490 |  |
|  | Environmental science | 2.96 | 1.497 |  |
|  | Geology | 3.30 | 1.767 |  |
|  | Hydrology | 2.50 | 1.581 |  |
|  | Information science | 2.51 | 1.541 |  |
|  | Law | 4.00 | .000 |  |
|  | Medicine/Health Sciences | 2.33 | 1.371 |  |
|  | Physical sciences | 3.09 | 1.756 |  |
|  | Psychology | 2.38 | 1.685 |  |
|  | Social sciences | 2.65 | 1.402 |  |
|  | Other (please specify) | 2.17 | 1.435 |  |
|  | Humanities | 1.00 | . |  |
|  |  |  |  |  |
| ... beyond the life of a research project (long term) | Agriculture and Natural Resources | 2.75 | 1.498 | F= .546  P= .935 |
|  | Atmospheric science | 2.64 | 1.367 |  |
|  | Biology | 2.22 | 1.313 |  |
|  | Business | 2.40 | 1.673 |  |
|  | Computer science | 2.23 | 1.301 |  |
|  | Ecology | 2.42 | 1.525 |  |
|  | Education | 2.22 | 1.093 |  |
|  | Engineering | 2.50 | 1.469 |  |
|  | Environmental science | 2.47 | 1.475 |  |
|  | Geology | 2.90 | 1.792 |  |
|  | Hydrology | 2.44 | 1.504 |  |
|  | Information science | 2.43 | 1.577 |  |
|  | Law | 2.50 | .707 |  |
|  | Medicine/Health Sciences | 1.75 | .965 |  |
|  | Physical sciences | 2.26 | 1.453 |  |
|  | Psychology | 2.13 | 1.642 |  |
|  | Social sciences | 2.43 | 1.409 |  |
|  | Other (please specify) | 2.00 | 1.414 |  |
|  | Humanities | 2.00 | . |  |
|  |  |  |  |  |
| ...during the life of a research project (short term) | Agriculture and Natural Resources | 3.30 | 1.363 | F= 1.05  P= .401 |
|  | Atmospheric science | 3.39 | 1.286 |  |
|  | Biology | 2.43 | 1.562 |  |
|  | Business | 3.20 | 1.483 |  |
|  | Computer science | 3.54 | 1.330 |  |
|  | Ecology | 3.03 | 1.478 |  |
|  | Education | 3.56 | 1.509 |  |
|  | Engineering | 3.15 | 1.599 |  |
|  | Environmental science | 2.75 | 1.480 |  |
|  | Geology | 3.60 | 1.506 |  |
|  | Hydrology | 3.00 | 1.188 |  |
|  | Information science | 3.11 | 1.471 |  |
|  | Law | 3.00 | 1.414 |  |
|  | Medicine/Health Sciences | 2.58 | 1.505 |  |
|  | Physical sciences | 2.96 | 1.665 |  |
|  | Psychology | 2.75 | 1.909 |  |
|  | Social sciences | 3.35 | 1.301 |  |
|  | Other (please specify) | 2.65 | 1.496 |  |
|  | Humanities | 4.00 | . |  |
|  |  |  |  |  |
| ...beyond the life of a research project (long term) | Agriculture and Natural Resources | 2.85 | 1.424 | F= .972  P= .491 |
|  | Atmospheric science | 2.96 | 1.401 |  |
|  | Biology | 2.26 | 1.322 |  |
|  | Business | 2.60 | 1.817 |  |
|  | Computer science | 2.69 | 1.109 |  |
|  | Ecology | 2.57 | 1.455 |  |
|  | Education | 2.67 | 1.581 |  |
|  | Engineering | 2.85 | 1.424 |  |
|  | Environmental science | 2.43 | 1.384 |  |
|  | Geology | 2.90 | 1.729 |  |
|  | Hydrology | 2.72 | 1.406 |  |
|  | Information science | 2.89 | 1.510 |  |
|  | Law | 2.00 | .000 |  |
|  | Medicine/Health Sciences | 1.58 | .900 |  |
|  | Physical sciences | 2.43 | 1.674 |  |
|  | Psychology | 2.75 | 1.909 |  |
|  | Social sciences | 2.87 | 1.392 |  |
|  | Other (please specify) | 2.30 | 1.428 |  |
|  | Humanities | 4.00 | . |  |
|  |  |  |  |  |

Table shows mean agreement (1= Disagree strongly, 2= disagree somewhat, 3= neither agree nor disagree, 4= agree somewhat, 5= agree strongly) and standard deviation for each item. MANOVA: F(180, 3722.78) = 1.03, *p* = .364, Wilks’ Lambda= .651, partial eta squared= .042.

Univariate ANOVAs for each item within omnibus MANOVA are reported. Only one participant from Humanities answered each item within the MANOVA, so there is no SD to report. All other statistics are reported in the results.
