# Supplementary material for: Changes in Data Sharing and Data Reuse Practices and Perceptions among Scientists Worldwide
Source: PLoS One. 2015 Aug 26;10(8):e0134826. doi: 10.1371/journal.pone.0134826 (PMC4550246; doi:10.1371/journal.pone.0134826)
Supplement: S2 Appendix — (DOCX) [file pone.0134826.s002.docx]

**S2 Appendix: Survey Questionnaire**

**Scientists and research data: Continuing to build an understanding of your data needs**

You are invited to participate in an NSF-sponsored research study, in which the DataONE (Data Observation Network for Earth, [www.dataone.org](http://www.dataone.org)) organization is investigating how scientists work. Your responses will help us better understand how scientists manage their data, which will then allow DataONE to better serve their data management needs.

The questionnaire should take about 20 minutes to complete. In addition to demographic information, other questions relate to the data management practices of scientists, the data education practices of scientists who are also educators, and finally how your organization and how designated data managers are involved with your research data. As such, no sensitive items are included in our survey, and therefore we do not anticipate that your participation poses any more than minimal risk. Also, your responses will be recorded anonymously so that no one can link your responses to you personally.

Your participation in this research is voluntary, and you may decline to participate without risk. While it is useful to be complete in your responses to the survey, you may skip any questions, and you are free to withdraw from the study at any time.

If you have any questions about the study or procedures, please contact Dr. Carol Tenopir (ctenopir@utk.edu) or Dr. Suzie Allard ([sallard@utk.edu](mailto:sallard@utk.edu)) of the University of Tennessee.  If you have questions about your rights as a participant, contact the Office of the Research Compliance Officer ([blawson@utk.edu](mailto:blawson@utk.edu)) or (865) 974-7697.

If you would like to keep a copy of this consent statement, you can save or print this page.

**By proceeding to the survey I acknowledge that I have read the above statements, I am 18 years old or older, and I agree to participate.**

**<Core Survey>**

First, we would like to ask you a few questions about yourself.

**1) Which one of the following best describes your primary work sector?**

 🔾 Academic
 🔾 Government
 🔾 Commercial
 🔾 Non-profit
 🔾 Other (please specify)

**2) What percentage of your work time is allocated to the following activities? (total to equal 100%)**

| Administration  |  |
| --- | --- |
| Outreach  |  |
| Policy support  |  |
| Research  |  |
| Teaching  |  |
| Data Management  |  |
| Other  |  |
|  |  |

If you selected other, please specify

**3) Which one of the following best describes your primary subject discipline?**

 🔾 Agriculture and Natural Resources

🔾 Atmospheric science
 🔾 Biology
 🔾 Business
 🔾 Computer science
 🔾 Ecology
 🔾 Education
 🔾 Engineering
 🔾 Environmental science
 🔾 Geology
 🔾 Hydrology
 🔾 Information science
 🔾 Law
 🔾 Medicine
 🔾 Physical sciences
 🔾 Psychology
 🔾 Social sciences
 🔾 Other (please specify)

**4) My current position is:**
 🔾 Administrator
 🔾 Assistant Professor
 🔾 Associate Professor
 🔾 Professor
 🔾 Graduate student
 🔾 Undergraduate
 🔾 Lecturer
 🔾 Post-doctoral fellow
 🔾 Researcher
 🔾 Other (please specify)

**5) Which of the following countries is your primary place of employment?**

**[To be displayed as a drop-down menu]**

**[If not United States of America, go to question 6]**

## 5b) Please also indicate your primary state (or US territory) of employment.

**[To be displayed as a drop-down menu]**

**6) Year of birth – Please select from the list below.**

**[To be displayed as a drop-down menu]**

**The next group of questions refers to your research and research data.**

**7) Which terms best describe the type of data you use? (Choose all that apply.)**

 ❑ Abiotic surveys (soils, microclimate, hydrology, etc.)
 ❑ Biotic surveys
 ❑ Data models
 ❑ Experimental (involving some degree of manipulation)
 ❑ Interviews
 ❑ Observational (no manipulation involved)
 ❑ Remote-sensed abiotic data (including meteorological data)
 ❑ Remote-sensed biotic data
 ❑ Social Science Survey
 ❑ Other (please specify)

**8) What metadata standards do you currently use to describe your data, if any? (Choose all that apply.)**

 ❑ DC (Dublin Core)
 ❑ DwC (Darwin Core)
 ❑ DIF (Directory Interchange Format)
 ❑ EML (Ecological Metadata Language)
 ❑ FGDC (Federal Geographic Data Committee)
 ❑ ISO 19115 (Geographic Information – Metadata)

❑ Other ISO metadata standard

❑ OGIS (Open GIS)

❑ ANZLIC metadata profile

❑ metadata standardized within my institution

❑ metadata standardized within my lab

❑ none

❑ Other (please specify)

**9) Which of the following best describes the primary funding agency for your research?**
 🔾 Federal/national government
 🔾 State/regional government
 🔾 Local government
 🔾 Corporation
 🔾 Private foundation

🔾 Internal/my institution
 🔾 Other (please specify)

**10) Does your primary funding agency require you to provide a data management plan?**
 🔾 Yes
 🔾 No
 🔾 Don't know

**11) Which of the following do you use to access or submit data? (Choose all that apply.)**
 ❑ DAAC (A Distributed Active-Archive Center)

❑ DataONE (Data Observation Network for Earth)

❑ Dryad

❑ ESA (Ecological Society of America)

❑ GBIF (A Global Biodiversity Information Facility)

❑ ILTER (International Long-term Ecological Research Network)

❑ KNB (Knowledge Network for Biocomplexity)

❑ LTER (Long-term Ecological Research Network)
 ❑ NEON (National Ecological Observatory Network)

❑ ORNL DAAC (Oak Ridge National Laboratory Distributed Active Archive Center)

❑ PISCO (Partnership for Interdisciplinary Study of Coastal Oceans)
 ❑ SAEON (South African Environmental Observation Network)

❑ SanParks (South Africa National Parks)
 ❑ TERN (Taiwan Ecological Research Network)

❑ UC3 (University of California Curation Center)

❑ USGS CSC (USGS Core Sciences Clearinghouse)

❑ Another organization-specific system (please specify)

❑ Other (please specify)

❑ None

**12) How much of your data do you currently store in the following locations? (For each location, choose only the one best answer.)**

|  | None of my data | Some of my data | Most of my data | All of my data | Not sure |
| --- | --- | --- | --- | --- | --- |
| on my institution’s server | 🔾 | 🔾 | 🔾 | 🔾 | 🔾 |
| on the principal investigator’s server | 🔾 | 🔾 | 🔾 | 🔾 | 🔾 |
| on a departmental server | 🔾 | 🔾 | 🔾 | 🔾 | 🔾 |
| on my personal computer | 🔾 | 🔾 | 🔾 | 🔾 | 🔾 |
| on paper in my office | 🔾 | 🔾 | 🔾 | 🔾 | 🔾 |
| in a discipline-based repository, (e.g. NEON or LTER) | 🔾 | 🔾 | 🔾 | 🔾 | 🔾 |
| in a publisher or publisher-related repository (e.g., specific publisher or Dryad) | 🔾 | 🔾 | 🔾 | 🔾 | 🔾 |
| other data repository or archive (e.g., national data center) | 🔾 | 🔾 | 🔾 | 🔾 | 🔾 |
| in my institution’s repository | 🔾 | 🔾 | 🔾 | 🔾 | 🔾 |
| other | 🔾 | 🔾 | 🔾 | 🔾 | 🔾 |

If you selected other, please specify

**13) How much of your data do you make available to others?**

🔾 none
 🔾 some
 🔾 most
 🔾 all

**[If all, go to question 14]**

**13b) If all or part of your data are not available to others, why not? (Choose all that apply.)**

 ❑ Lack of funding
 ❑ Lack of standards
 ❑ People don’t need them
 ❑ There is insufficient time to make them available
 ❑ There is no place to put them
 ❑ They shouldn't be available
 ❑ Sponsor doesn't require it
 ❑ Don't have the rights to make the data public

❑ I would lose control of the data

❑ I need to publish first
 ❑ I have insufficient skills to make my data available
 ❑ Other (please specify)

**14) The following statements relate to how you collect and use research data. Tell us how much you agree with the following ways to complete this sentence:**

**I am satisfied with the…….**

|  | agree strongly | agree somewhat | neither agree nor disagree | disagree somewhat | disagree strongly | not sure |
| --- | --- | --- | --- | --- | --- | --- |
| …….process for collecting my research data. | 🔾 | 🔾 | 🔾 | 🔾 | 🔾 | 🔾 |
| …….process for cataloging / describing my data. | 🔾 | 🔾 | 🔾 | 🔾 | 🔾 | 🔾 |
| …….process for storing my data during the life of the project (short-term). | 🔾 | 🔾 | 🔾 | 🔾 | 🔾 | 🔾 |
| …….process for storing my data beyond the life of the project (long-term). | 🔾 | 🔾 | 🔾 | 🔾 | 🔾 | 🔾 |
| …….process for searching for my own data. | 🔾 | 🔾 | 🔾 | 🔾 | 🔾 | 🔾 |
| …….process for analyzing my data. | 🔾 | 🔾 | 🔾 | 🔾 | 🔾 | 🔾 |
| …….tools for preparing metadata. | 🔾 | 🔾 | 🔾 | 🔾 | 🔾 | 🔾 |
| …….tools for preparing my documentation. | 🔾 | 🔾 | 🔾 | 🔾 | 🔾 | 🔾 |

**15) The following statements relate to data sharing and access. Tell us how much you agree with each statement.**

|  | agree strongly | agree somewhat | neither agree nor disagree | disagree somewhat | disagree strongly | not sure |
| --- | --- | --- | --- | --- | --- | --- |
| I share my data with others. | 🔾 | 🔾 | 🔾 | 🔾 | 🔾 | 🔾 |
| Others need my permission to access my data. | 🔾 | 🔾 | 🔾 | 🔾 | 🔾 | 🔾 |
| Others can access my data easily. | 🔾 | 🔾 | 🔾 | 🔾 | 🔾 | 🔾 |

**16) The following statements relate to conditions for use of your data. Indicate whether you agree or disagree with each condition.**

|  | For others to use my data, I would expect the following in exchange: | | |
| --- | --- | --- | --- |
|  | yes | no | not sure |
| co-authorship on publications resulting from use of the data. | 🔾 | 🔾 | 🔾 |
| acknowledgement of the data providers in all disseminated work making use of the data. | 🔾 | 🔾 | 🔾 |
| citation of the data providers in all disseminated work making use of the data. | 🔾 | 🔾 | 🔾 |
| the opportunity to collaborate on a project using the data. | 🔾 | 🔾 | 🔾 |
| results based (at least in part) on the data could not be disseminated in any format without the data provider's approval. | 🔾 | 🔾 | 🔾 |
| at least part of the costs of data acquisition, retrieval or provision must be recovered. | 🔾 | 🔾 | 🔾 |
| results based (at least in part) on the data could not be disseminated without the data provider having the opportunity to review the results and make suggestions or comments, but approval not required. | 🔾 | 🔾 | 🔾 |
| reprints of articles that make use of the data must be provided to the data provider. | 🔾 | 🔾 | 🔾 |
| the data provider is given a complete list of all products that make use of the data, including articles, presentations, educational materials, etc. | 🔾 | 🔾 | 🔾 |
| legal permission for data use is obtained. | 🔾 | 🔾 | 🔾 |
| mutual agreement on reciprocal sharing of data. | 🔾 | 🔾 | 🔾 |
| the data provider is given and agrees to a statement of uses to which the data will be put. | 🔾 | 🔾 | 🔾 |

We are also interested in how your organization is involved with research data.

**17) The following statements relate to how your organization is involved with managing and storing data. Tell us how much you agree with the following ways to complete this sentence:**

**My organization has a formal process for…….**

|  | agree strongly | agree somewhat | neither agree nor disagree | disagree somewhat | disagree strongly | not sure |
| --- | --- | --- | --- | --- | --- | --- |
| …….managing data during the life of the project (short-term). | 🔾 | 🔾 | 🔾 | 🔾 | 🔾 | 🔾 |
| …….storing data beyond the life of the project (long-term). | 🔾 | 🔾 | 🔾 | 🔾 | 🔾 | 🔾 |

**17A) [If no agreement with Q17 (strongly or somewhat, short-term or long-term), go to Q18]**

You have expressed agreement (strongly or somewhat) that your organization or project has a formal process for managing or storing data during or beyond the life of the project (short-term or long-term). Which of the following are involved with this process? (Choose all that apply.)

❑ Research support unit(s) (e.g. Office of Research, Office of Sponsored Programs and Contracts)

❑ The library

❑ Information technology support unit(s) (e.g. Office of Information Technology, IT Support Center)

❑ Administrative office(s) (e.g. Department Heads, Deans, Provosts, Program Offices, Research Offices, Divisions, Directorates / Directors, Managers)

❑ Designated data manager(s)

❑ Colleagues in my own unit / department

❑ Other (please specify)

**18) The following statements relate to how your organization is involved with training. Tell us how much you agree with the following ways to complete this sentence:**

**My organization or project provides training or assistance on…….**

|  | agree strongly | agree somewhat | neither agree nor disagree | disagree somewhat | disagree strongly | not sure |
| --- | --- | --- | --- | --- | --- | --- |
| …….training on best practices for data management. | 🔾 | 🔾 | 🔾 | 🔾 | 🔾 | 🔾 |
| …….assistance on creating data management plans. | 🔾 | 🔾 | 🔾 | 🔾 | 🔾 | 🔾 |
| …….assistance on creating metadata to describe my data or datasets. | 🔾 | 🔾 | 🔾 | 🔾 | 🔾 | 🔾 |
| …….training on how to cite datasets. | 🔾 | 🔾 | 🔾 | 🔾 | 🔾 | 🔾 |

**18A) [If no agreement (strongly or somewhat) with Q18.1, go to Q18B]**

You have expressed agreement (strongly or somewhat) that your organization or project provides training on best practices for data management. Which of the following provides this training? (Choose all that apply.)

❑ Research support unit(s) (e.g. Office of Research, Office of Sponsored Programs and Contracts)

❑ The library

❑ Information technology support unit(s) (e.g. Office of Information Technology, IT Support Center)

❑ Administrative office(s) (e.g. Department Heads, Deans, Provosts, Program Offices, Research Offices, Divisions, Directorates / Directors, Managers)

❑ Designated data manager(s)

❑ Colleagues in my own unit/department

❑ Other (please specify)

**18B) [If no agreement (strongly or somewhat) with Q18.2, go to Q18C]**

You have expressed agreement (strongly or somewhat) that your organization provides assistance on creating data management plans. Which of the following provides this assistance? (Choose all that apply.)

❑ Research support unit(s) (e.g. Office of Research, Office of Sponsored Programs and Contracts)

❑ The library

❑ Information technology support unit(s) (e.g. Office of Information Technology, IT Support Center)

❑ Administrative office(s) (e.g. Department Heads, Deans, Provosts, Program Offices, Research Offices, Divisions, Directorates / Directors, Managers)

❑ Designated data manager(s)

❑ Colleagues in my own unit/department

❑ Other (please specify)

**18C) [If no agreement (strongly or somewhat) with Q18.3, go to Q18D]**

You have expressed agreement (strongly or somewhat) that your organization provides assistance on creating metadata to describe your data or datasets. Which of the following provides this assistance? (Choose all that apply.)

❑ Research support unit(s) (e.g. Office of Research, Office of Sponsored Programs and Contracts)

❑ The library

❑ Information technology support unit(s) (e.g. Office of Information Technology, IT Support Center)

❑ Administrative office(s) (e.g. Department Heads, Deans, Provosts, Program Offices, Research Offices, Divisions, Directorates / Directors, Managers)

❑ Designated data manager(s)

❑ Colleagues in my own unit/department

❑ Other (please specify)

**18D) [If no agreement (strongly or somewhat) with Q18.4, go to Q19-1]**

You have expressed agreement (strongly or somewhat) that your organization provides training on how to cite datasets. Which of the following provides this training? (Choose all that apply.)

❑ Research support unit(s) (e.g. Office of Research, Office of Sponsored Programs and Contracts)

❑ The library

❑ Information technology support unit(s) (e.g. Office of Information Technology, IT Support Center)

❑ Administrative office(s) (e.g. Department Heads, Deans, Provosts, Program Offices, Research Offices, Divisions, Directorates / Directors, Managers)

❑ Designated data manager(s)

❑ Colleagues in my own unit/department

❑ Other (please specify)

**19-1) The following statements relate to how your organization is involved with funding for data management. Tell us how much you agree with the following ways to complete this sentence:**

**My organization or project provides the funds to support data management…….**

|  | agree strongly | agree somewhat | neither agree nor disagree | disagree somewhat | disagree strongly | not sure |
| --- | --- | --- | --- | --- | --- | --- |
| …….during the life of a research project (short-term). | 🔾 | 🔾 | 🔾 | 🔾 | 🔾 | 🔾 |
| …….beyond the life of a research project (long-term). | 🔾 | 🔾 | 🔾 | 🔾 | 🔾 | 🔾 |

**19-1A) [If no agreement with Q19-1 (strongly or somewhat, short-term or long-term), go to Q19-2]**

You have expressed agreement (strongly or somewhat) that your organization or project provides the necessary funds to support data management during or beyond the life of the project (short-term or long-term). Which of the following are involved with this process? (Choose all that apply.)

❑ Research support unit(s) (e.g. Office of Research, Office of Sponsored Programs and Contracts)

❑ The library

❑ Information technology support unit(s) (e.g. Office of Information Technology, IT Support Center)

❑ Administrative office(s) (e.g. Department Heads, Deans, Provosts, Program Offices, Research Offices, Divisions, Directorates / Directors, Managers)

❑ Designated data manager(s)

❑ Colleagues in my own unit / department

❑ Other (please specify)

**19-2) The following statements relate to how your organization is involved with software tools and technical support. Tell us how much you agree with the following ways to complete this sentence:**

**My organization or project provides the software tools and technical support for data management…….**

|  | agree strongly | agree somewhat | neither agree nor disagree | disagree somewhat | disagree strongly | not sure |
| --- | --- | --- | --- | --- | --- | --- |
| …….during the life of the project (short-term). | 🔾 | 🔾 | 🔾 | 🔾 | 🔾 | 🔾 |
| …….beyond the life of the project (long-term). | 🔾 | 🔾 | 🔾 | 🔾 | 🔾 | 🔾 |

**19-2A) [If no agreement (strongly or somewhat, short-term or long-term) with Q19-2, go to Q20]**

You have expressed agreement (strongly or somewhat) that your organization or project provides the necessary tools and technical support for data management during or beyond the life of the project (short-term or long-term). Which of the following provide this service? (Choose all that apply.)

❑ Research support unit(s) (e.g. Office of Research, Office of Sponsored Programs and Contracts)

❑ The library

❑ Information technology support unit(s) (e.g. Office of Information Technology, IT Support Center)

❑ Administrative office(s) (e.g. Department Heads, Deans, Provosts, Program Offices, Research Offices, Divisions, Directorates / Directors, Managers)

❑ Designated data manager(s)

❑ Colleagues in my own unit/department

❑ Other (please specify)

**Next we would like to ask you about your views on data use and reuse in science.**

**20) The following statements relate to your views on the use of scientific research data. Tell us how much you agree with each statement.**

|  | agree strongly | agree somewhat | neither agree nor disagree | disagree somewhat | disagree strongly | not sure |
| --- | --- | --- | --- | --- | --- | --- |
| Lack of access to data generated by other researchers or institutions is a major impediment to progress in science. | 🔾 | 🔾 | 🔾 | 🔾 | 🔾 | 🔾 |
| Lack of access to data generated by other researchers or institutions has restricted my ability to answer scientific questions. | 🔾 | 🔾 | 🔾 | 🔾 | 🔾 | 🔾 |
| Data may be misinterpreted due to complexity of the data. | 🔾 | 🔾 | 🔾 | 🔾 | 🔾 | 🔾 |
| Data may be misinterpreted due to poor quality of the data. | 🔾 | 🔾 | 🔾 | 🔾 | 🔾 | 🔾 |
| Data may be used in other ways than intended. | 🔾 | 🔾 | 🔾 | 🔾 | 🔾 | 🔾 |

**21) The following statements relate to sharing scientific data. Tell us how much you agree with each statement.**

|  | agree strongly | agree somewhat | neither agree nor disagree | disagree somewhat | disagree strongly | not sure |
| --- | --- | --- | --- | --- | --- | --- |
| I would use other researchers' datasets if their datasets were easily accessible. | 🔾 | 🔾 | 🔾 | 🔾 | 🔾 | 🔾 |
| I would be willing to place at least some of my data into a central data repository with no restrictions. | 🔾 | 🔾 | 🔾 | 🔾 | 🔾 | 🔾 |
| I would be willing to place all of my data into a central data repository with no restrictions. | 🔾 | 🔾 | 🔾 | 🔾 | 🔾 | 🔾 |
| I would be more likely to make my data available if I could place conditions on access. | 🔾 | 🔾 | 🔾 | 🔾 | 🔾 | 🔾 |
| I am satisfied with my ability to integrate data from disparate sources to address research questions. | 🔾 | 🔾 | 🔾 | 🔾 | 🔾 | 🔾 |
| I would be willing to share data across a broad group of researchers. | 🔾 | 🔾 | 🔾 | 🔾 | 🔾 | 🔾 |
| It is important that my data are cited when used by other researchers. | 🔾 | 🔾 | 🔾 | 🔾 | 🔾 | 🔾 |
| It is appropriate to create new datasets from shared data. | 🔾 | 🔾 | 🔾 | 🔾 | 🔾 | 🔾 |

*Additional sections were included in data collection, but not analyzed in the current study. These questions pertained specifically to DataONE familiarity and use; Data management education; and an optional module about sociocultural norms in data sharing and reuse.*

*Separate analyses for these sections are forthcoming.*

**Your survey is now complete.**

**Thank you very much!**
